# Classification of Intraoral Photographs with Deep Learning Algorithms Trained According to Cephalometric Measurements

**DOI:** 10.3390/diagnostics15091059

**Published:** 2025-04-22

**Authors:** Sultan Büşra Ay Kartbak, Mehmet Birol Özel, Duygu Nur Cesur Kocakaya, Muhammet Çakmak, Enver Alper Sinanoğlu

**Affiliations:** 1 Department of Orthodontics, Faculty of Dentistry, Kocaeli University, Kocaeli 41190, Türkiye; sultanbusraay@gmail.com; 2 Private Practice, Gölcük 41650, Türkiye; duyguucesur@gmail.com; 3 Department of Computer Engineering, Faculty of Engineering and Architecture, Sinop University, Sinop 57000, Türkiye; mcakmak@sinop.edu.tr; 4 Department of Oral and Maxillofacial Radiology, Faculty of Dentistry, Kocaeli University, Kocaeli 41190, Türkiye; alper.sinanoglu@kocaeli.edu.tr

**Keywords:** deep learning, artificial intelligence, intraoral photograph, cephalometry

## Abstract

**Background/Objectives**: Clinical intraoral photographs are important for orthodontic diagnosis, treatment planning, and documentation. This study aimed to evaluate deep learning algorithms trained utilizing actual cephalometric measurements for the classification of intraoral clinical photographs. **Methods**: This study was executed on lateral cephalograms and intraoral right-side images of 990 patients. IMPA, interincisal angle, U1–palatal plane angle, and Wits appraisal values were measured utilizing WebCeph. Intraoral photographs were divided into three groups based on cephalometric measurements. A total of 14 deep learning models (DenseNet 121, DenseNet 169, DenseNet 201, EfficientNet B0, EfficientNet V2, Inception V3, MobileNet V2, NasNetMobile, ResNet101, ResNet152, ResNet50, VGG16, VGG19, and Xception) were employed to classify the intraoral photographs. Performance metrics (F1 scores, accuracy, precision, and recall) were calculated and confusion matrices were formed. **Results**: The highest accuracy rates were 98.33% for IMPA groups, 99.00% for interincisal angle groups, 96.67% for U1–palatal plane angle groups, and 98.33% for Wits measurement groups. Lowest accuracy rates were 59% for IMPA groups, 53% for interincisal angle groups, 33.33% for U1–palatal plane angle groups, and 83.67% for Wits measurement groups. **Conclusions**: Although accuracy rates varied among classifications and DL algorithms, successful classification could be achieved in the majority of cases. Our results may be promising for case classification and analysis without the need for lateral cephalometric radiographs.

## 1. Introduction

Artificial intelligence (AI) is defined as the ability of computers to present intelligent behavior with minimal human guidance [1,2]. Increasing data availability, computing power, and advances in analytical methods have increased the use of AI [3]. Machine learning (ML) is the primary foundation of AI. It aims to make it easier for machines to learn from data in order to solve problems without the need for human intervention [4]. Deep learning (DL) is a subclass of machine learning. The predominant algorithm in AI image analysis is DL, that runs convolutional neural networks (CNNs) [5]. The potential of deep learning for automating the interpretation of medical images, improving clinical decision-making, and determining optimal treatment paths for intricate diseases is continuously growing [6].

In every facet of orthodontics, including patient communication, diagnostic, and treatment procedures, AI-based software solutions are used [7]. Numerous studies have been published evaluating the potential of artificial intelligence for automated anatomic landmark detection, diagnosis and treatment planning, orthodontic extraction decisions, evaluation of growth and development, and determination of treatment need [8]. DL-based cephalometric software (e.g., CellmatIQ, Hamburg, Germany; ORCA AI, Herzliya, Israel; WebCeph, Hwaseong-si, Republic of Korea) is available to orthodontists [9].

Intraoral photographs are standard images required for orthodontic diagnosis and provide various information about tooth shape, alignment, and periodontal status [10]. With the advent of digital technologies, imaging has become easier and more accessible [11]. A full set of photographs is an invaluable record detailing the original clinical situation. Intraoral photographs are mandatory records that should be taken before diagnosis and treatment planning. They also allow the observation of specific changes that occur throughout the treatment [12].

Cephalometric analysis is primarily used to distinguish whether the anomaly is of dental or skeletal origin [5]. Evaluation of maxillary and mandibular incisor inclination using lateral cephalograms is a routine procedure in orthodontic diagnosis. Incisor inclination is an important factor for planning anteroposterior incisor movements within the jaws [13].

To date, the performance of DL models has not been tested on intraoral clinical photographs classified according to cephalometric measurements. Therefore, in this study, the aim was to evaluate the classification performance of different DL models using intraoral clinical photographs that had been preclassified based on actual cephalometric measurements.

## 2. Materials and Methods

The Ethics Committee of Kocaeli University (GOKAEK-2023/06.22) approved this study. Pretreatment intraoral right-side photos and cephalometric radiographic records of patients that had been incorporated into the Kocaeli University Faculty of Dentistry, Department of Orthodontics database, between 2014 and 2018 were randomly selected.

### 2.1. Study Group Selection

This study was carried out on 990 randomly selected cephalometric radiographs and 990 intraoral side photographs of patients, taken at the same session. Patients did not receive any prior orthodontic treatment. The exclusion criteria were a history of orthodontic or orthognathic treatment, cleft lip and palate, and/or syndromic craniofacial anomalies. Lateral cephalometric radiographs containing artefacts or images that exhibited poor image quality were also excluded.

Lateral cephalograms were taken with the same X-ray device (J. Morita MFG. Corp. Veraviewepocs 2D, Kyoto, Japan). Magnification difference was 1.1 mm, as determined by the manufacturer. Lateral cephalometric radiographs were made while the patients were in the neutral head position, with maximum intercuspal occlusion and relaxed lips.

A Nikon D700 digital camera and a Nikon AF-S VR Micro Nikkor 105 mm f/2.8 G IF ED lens (Nikon Corporation, Tokyo, Japan) were used for taking intraoral right-side photographs with maximum lip retraction and the occlusal plane parallel to the ground.

### 2.2. Cephalometric Measurements

Cephalometric measurements were made with the WebCeph^TM^ digital cephalometric measurement program (AssembleCircle Corp., Hwaseong-si, Republic of Korea). Image calibration was performed by marking two points known to be 10 mm in length on the cephalostat rod on the radiograph with an application ruler. After automatic digitization with WebCeph 2.0.0, anatomical landmarks were manually repositioned.

**Figure 1:**
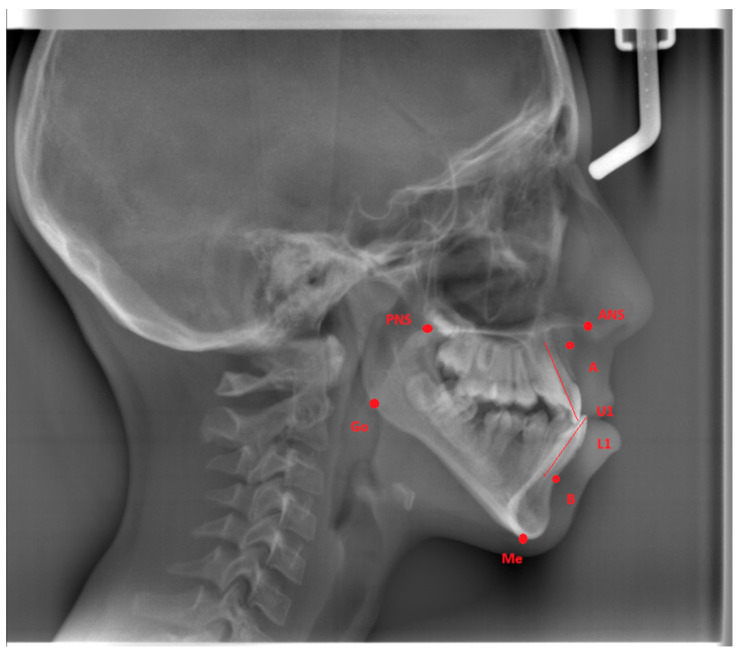
Cephalometric reference points. (ANS: The most anterior point of the maxilla at the nasal floor. PNS: The most posterior point of the maxilla at the nasal floor. A point: The deepest point of the anterior maxilla under ANS. B point: The deepest point of the anterior alveolus of the mandible. U1: The line between the incisal tip and the root apex of the upper central incisors. L1: The line between the incisal tip and the root apex of the lower central incisors. Gonion (Go): The meeting point of the posterior border of the ramus and the inferior border of the mandibular corpus. Menton (Me): The most inferior point on the mandibular symphysis.

**Figure 2:**
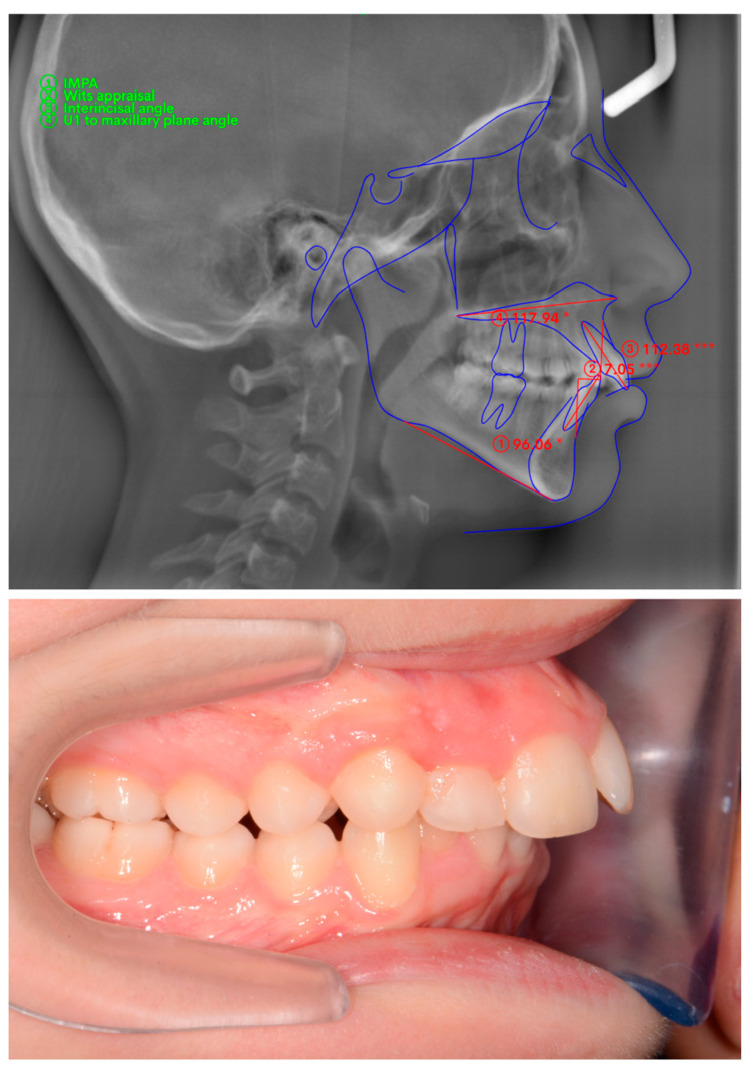
Cephalometric measurements on WebCeph and intraoral photographs. (Performed cephalometric measurements are demonstrated on the actual screenshot of WebCephTM digital cephalometric measurement program (AssembleCircle Corp., Hwaseong-si, Republic of Korea) interface. The “*” and “***” are automatically generated markers in order to point to the deviation of the measurement from norm values).

The A point, B point, Anterior Nasal Spine, Posterior Nasal Spine, Gonion, Menton, U1, and L1 were marked (Figure 1) and four measurements were made (Figure 2) on lateral cephalometric radiographs.

IMPA is the angle between the long axis of the lower incisor and the mandibular plane (Go-Me). It is used for determining the anteroposterior inclination of the lower incisors.

The U1–palatal plane (U1-PP) is the angle between the long axis of the upper central incisor and the line formed by the ANS-PNS points. The inclination of the upper incisors is determined by this angle.

The interincisal angle is the angle formed between the long axes of the lower and upper incisors. It enables the evaluation of the protrusion and retrusion relationship between the lower and upper incisors.

The Wits appraisal is the distance between the perpendicular projection points (AO and BO) lowered to the occlusal plane from points A and B. In Class II cases, the BO point is located posterior to the AO point and positive values are measured. In Class III cases, the Wits value is negative, and the BO point is located anterior to the AO point.

A total of 50 randomly selected lateral cephalometric radiographs were re-evaluated two weeks later by the same researcher to determine intra-observer agreement. Intraclass correlation coefficients were calculated using R (version 4.0.2, R Foundation for Statistical Computing, Vienna, Austria) and found to be between 0.89–0.98, presenting high reproducibility.

### 2.3. Intraoral Side Photograph Group Formation for CNN Algorithms

The cases were divided into three subgroups of equal number according to the order of the cephalometric measurements (Figure 2). The groups were created according to numerical equality between the groups instead of the cephalometric norm values.

IMPA values were 70.69°–91.84° in the first group (*n* = 330), 91.85°–98.08° in the second group (*n* = 330), and 98.15°–121.48° in the third group (*n* = 330).

U1-PP values were 78.31°–110.67° in the first group (*n* = 330), 110.69°–116.08° in the second group (*n* = 330) and 116.09°–131.71° in the third group (*n* = 330).

Interincisal Angle values were 95.70°–122.45° in the first group (*n* = 330), 122.47°–131.28° in the second group (*n* = 330), and 131.31°–169.49° in the third group (*n* = 330).

Wits values were between −19.04 mm and −0.34 mm in the first group (*n* = 330), −0.32 mm and 3.89 mm in the second group (*n* = 330), and 3.91 mm and 16.96 mm in the third group (*n* = 330).

### 2.4. Preparation of CNN Algorithms

Image classification techniques were put into practice at the Sinop University Faculty of Engineering and Architecture Software and Artificial Intelligence Laboratory. A total of 14 DL models (DenseNet 121, DenseNet 169, DenseNet 201, EfficientNet B0, EfficientNet V2, Inception V3, MobileNet V2, NasNetMobile, ResNet101, ResNet152, ResNet50, VGG16, VGG19, and Xception) were employed.

#### State of Art Models

DenseNet121, DenseNet169, and DenseNet201 are parameter-efficient networks with densely connected features that propagate the output of each layer to all subsequent layers. EfficientNetB0 and EfficientNetV2 achieve accuracy and efficiency by maximizing the depth, width, and resolution of the model with a scaling approach. InceptionV3 offers feature diversity by applying filters of different sizes simultaneously. MobileNetV2 is a lightweight model optimized for mobile and embedded devices. NasNetMobile has the ability to automatically discover the structure of the neural network. ResNet50, ResNet101, and ResNet152 are Residual Networks, with the vanishing gradient problem of deep networks addressed through skip connections, and show strong performance at depth. VGG16 and VGG19 offer a simple yet effective structure, maximizing the depth by applying small-size fixed filters. Xception offers an improved efficiency variation on the Inception structure by applying deep discrete convolutions.

### 2.5. Dataset

The dataset contained 330 original images of each class. Then, each group in the dataset was increased to 1000 images using data augmentation methods. The first step after the data augmentation process was creating training, validation, and test datasets for all deep learning models. Labeled samples formed these datasets. Of the entire dataset, 80% was used for training, 10% was used for validation, and the remaining 10% was the test dataset.

Data augmentation was applied to the grouped profile photos on all deep learning models in order to achieve better accuracy and lower loss values. Data augmentation is a technique used to diversify and expand the dataset by applying various transformations and changes to the existing dataset to create new samples. It is an effective method, especially when working with limited data, of increasing the model’s generalization ability and reducing overfitting. For this purpose, the original dataset was augmented using a series of image-processing techniques such as horizontal flipping, rotation, width shifting, height shifting, and zooming. The number of photographs in each group was increased from 330 to 1000 photographs.

Following dataset organization, the input data for the first convolutional layer in deep learning was subjected to several filters, or kernels. Every filter performs a convolution operation on the input data, multiplying and adding up the values of the pixels in the area where the filter will be applied. This sum generates a pixel of the feature map. Various features (edges, corners, patterns, etc.) can be extracted with the use of various filters. After each convolution operation, an activation function was applied to the obtained feature maps. The activation function limits the pixel values in the feature map and helps learn more nonlinear features. In the pooling layer, the dimensions of the feature maps were decreased, enhancing their robustness to variations in scale and position. Average pooling was used for better results. Pooling maintains the essence of important features in each feature map while reducing their size. The feature maps were flattened and sent to dense layers after the pooling layers. These layers are used to learn higher-level features. Each fully connected layer can interpret the features in the input data for classification. Following the last dense layer, the classification procedure was carried out in the output layer. The softmax activation function was employed for classification.

### 2.6. Performance Evaluation Methods

A GPU-supported system in the Google cloud environment was used in this experimental study to train deep learning models. An Intel Xeon CPU, operating at 2.20 GHz with 16 GB of RAM and a Tesla T4 GPU (NVIDIA Corporation, Santa Clara, CA, USA), was used for training. All the programs for the transfer learning design were written using the Python 3 and Keras 2.3.1 training framework.

All photographic images classified according to cephalometric measurement groups (*n* = 100) were tested for accuracy in predicting cephalometric classification via 14 DL algorithms (DenseNet 121, DenseNet 169, DenseNet 201, EfficientNet B0, EfficientNet V2, Inception V3, MobileNet V2, NasNetMobile, ResNet101, ResNet152, ResNet50, VGG16, VGG19, and Xception) following learning, training, and validation processes.

The formulas used, where the abbreviation “TP” is used for “True Positive”, “TN” for “True Negative”, “FP” for “False Positive”, and “FN” for “False Negative”, are as follows:

Accuracy = (TP + TN)/(TP + TN + FP + FN),

Precision = TP/(TP + FP),

Recall = TP/(TP + FP),

F1 score = 2 × (Recall × Precision)/(Recall + Precision).

Confusion matrices showing the number of correct and incorrect predictions of classification models with the existing situation in the dataset were also formed.

The analyses were conducted using Scikit-learn and Seaborn/Matplotlib libraries in Python 3.10.

## 3. Results

The accuracy values of 14 DL algorithms for four parameters are given in Table 1. Except for six DL algorithms for U1-PP (Inception V3, Xception, VGG16, VGG19, ResNet101, and ResNet152), which demonstrated the poorest accuracy values of 33.33–36.67%, precision, recall, and F1 scores were calculated and confusion matrices for every parameter and DL algorithm were plotted. This yielded 50 3 × 3 confusion matrices and 150 precision, 150 recall, and 150 F1 score data, which is beyond the presentable scope of one article. Therefore, this part of the data is provided as Supplementary Materials.

The Xception algorithm (98.33%) yielded the highest accuracy rate for IMPA, with 295 correct classifications out of 300 images, and VGG16 displayed the lowest accuracy rate, at 59.00%, with correct classifications for 177 images out of 300.

The highest accuracy rate for the Interincisal angle was 99.00% from EfficientNet V2, with 297 correct classifications out of 300 images. VGG19 was the algorithm with the lowest accuracy rate, at 51.67% and with correct classifications of 155 images out of 300.

The highest accuracy rate for the U1-PP angle was obtained using the EfficientNet B0 algorithm (96.67%), with 290 correct classifications out of 300 images. Aside from the excluded Inception V3, Xception, VGG16, VGG19, ResNet101, and ResNet152 algorithms, NasNetMobile presented the lowest accuracy rate at 81.00%, with correct classifications for 243 images out of 300.

The EfficientNet B0 algorithm demonstrated 98.33% accuracy, with the highest rate for the Wits value and 295 correct classifications out of 300 images. NasNetMobile exhibited the lowest accuracy rate at 83.67%, with 251 correct classifications out of 300 images.

### Grad-CAM Application

**Figure 3:**
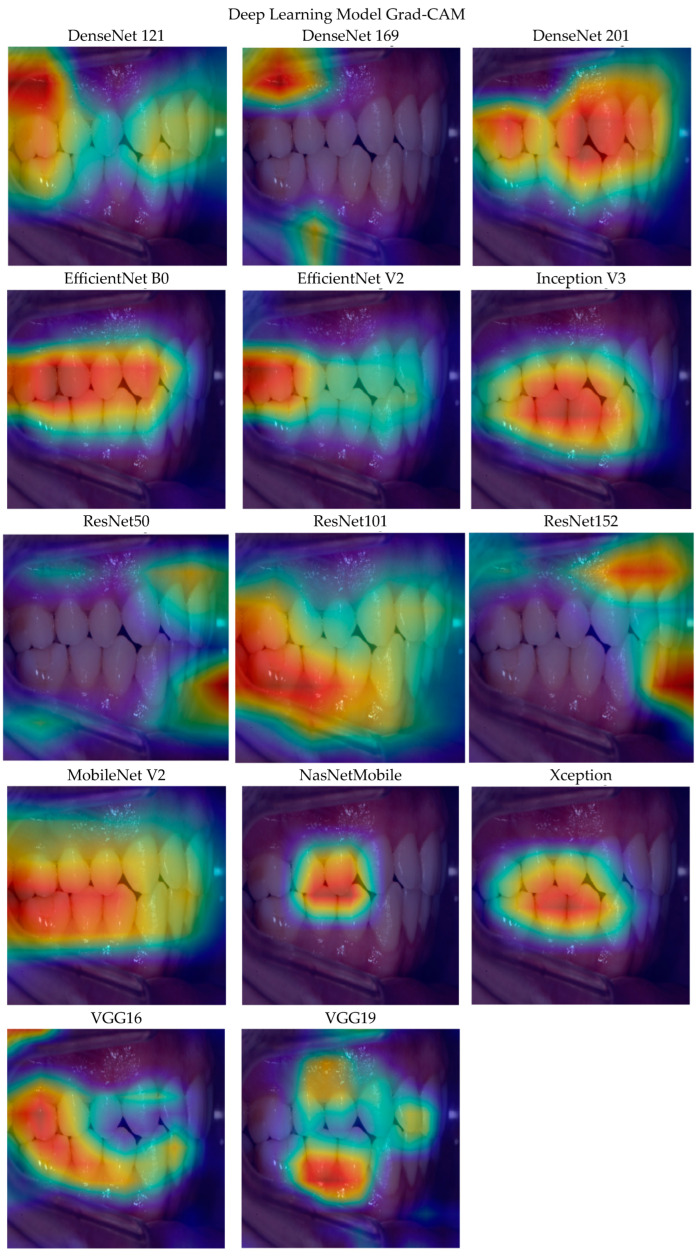
Grad-CAM analysis images of 14 DL algorithms. Red/Yellow colors denote high importance which strongly influence the prediction of the model. Orange color denotes less contribution to the prediction and blue/dark blue colors denote little to no effect and if present; black color denotes no contribution to the result.

In this section, Grad-CAM analysis images of DenseNet 121, DenseNet 169, DenseNet 201, EfficientNet B0, EfficientNet V2, Inception V3, MobileNet V2, NasNetMobile, ResNet101, ResNet152, ResNet50, VGG16, VGG19, and the Xception models are evaluated (Figure 3).

Grad-CAM analyses of 14 different deep learning models performed on intraoral images reveal that each model shows significant differences in terms of the regions it focuses its attention on. The DenseNet121 and DenseNet169 models produced more widespread and scattered attention maps. The focuses of both models are between the soft tissues surrounding the teeth. The DenseNet201 model is more dense, and was focused especially on the incisor and chewing surfaces.

EfficientNetB0 and EfficientNetV2 obtained a spatially consistent result by creating a symmetric and dense attention area along the dental arch. The heat maps obtained are consistent with the designs of the models aimed at balancing accuracy and processing efficiency. The InceptionV3 model also focused on the teeth in the middle region and clearly showed the chewing surfaces. Inception V3 is effective in model inference.

While ResNet50 and ResNet152 produced more scattered and shifted attention regions to the edges, the ResNet101 model focused especially on the interdental surfaces and chewing areas of the teeth. The scatteredness observed in ResNet152 suggests that deeper architectures may be prone to over-learning without regularization.

It is noteworthy that MobilNetV2 produces a clear Grad-CAM map concentrated on the midline. MobileNetV2 demonstrates that it can capture diagnostically meaningful regions despite its limited number of parameters. NasNetMobile, on the other hand, produced a very narrow attention field focused only on the incisors. Although NasNetMobile limits the overall image understanding, it can provide high sensitivity in small regions. The Xception model, on the other hand, produced a wider oval-shaped attention map, demonstrating that it can capture both global and local features together.

VGG16 and VGG19 produced more scattered attention maps spread over the upper and lower teeth and surrounding tissues. While both models are good in terms of context-sensitive information extraction, they can also cause problems in distracting attention from the diagnostic focus.

In conclusion, the DenseNet201, EfficientNetV2, ResNet101, and MobileNetV2 models provide the clearest, most focused, and most clinically meaningful visualizations in Grad-CAM analysis.

## 4. Discussion

Lateral cephalometric radiographs are frequently implemented for orthodontic assessment and treatment planning [14]. Despite their utility, radiographs carry a risk of ionizing radiation exposure, potentially leading to somatic stochastic effects and increased cancer risk over a patient’s lifetime. Therefore, clinicians must judiciously limit patients’ exposure to diagnostic radiation, considering its cumulative effects [14]. Bruks et al. stated that clinical examination supported by diagnostic models and photographs provides sufficient information for orthodontic treatment planning, and that cephalometric radiograph is not mandatory for every patient [15].

Intraoral photographs are highly beneficial due to their non-invasive and non-radioactive nature, allowing clinicians to examine a patient’s oral problems in detail for an extended period [16]. In the study by Jackson et al., comparing intraoral photographs with diagnostic models for evaluating diagnostic accuracy, angle classification accuracy from photographs, when taken at an ideal camera angle, was found to be 79.86% for molar classification and 51.29% for canine classification, suggesting that even ideal photographs may lead to false evaluations compared to diagnostic models in up to 20% of cases [17]. In another study, Ryu et al. investigated DL algorithms to evaluate the crowding and extraction decisions of four trained DL algorithms using occlusal photographs [18]. Recent advances, including teledentistry techniques, increasingly rely on photographs and video-based interactions for diagnosis rather than face-to-face examinations [17]. This study was an attempt to address these developments by testing the ability of artificial intelligence algorithms to carry out diagnostic procedures on photographs. The high success rates of the DL algorithms in this study are promising in this regard.

In order to offer a broader perspective on the potential of existing DL algorithms, in this study, we analyzed cephalometric radiographs and intraoral right-side buccal photographs from 990 patients using 14 DL models. Although an established sample size calculation method is not currently available for training artificial neural networks, the sample size in our present study was derived based on methodologies from similar research. Seo et al. utilized 100 cephalometric radiographs per group, totaling 600 images, in their study which assessed cervical vertebra maturation degrees using deep learning techniques applied to cephalometric radiographs [19]. Mohammad-Rahimi et al. [6] used 890 cephalograms and tested 10 DL algorithms on classifying cervical vertebra maturation degrees. Unlike in that study, we formed three groups containing an equal number of cases in order to avoid imbalance between the groups.

In our study, classifications of cases were made using three angular and one linear cephalometric measurement, and the performance of deep learning algorithms in classifying intraoral photographs without a cephalometric radiograph was evaluated. The best success rates were reached by Xception for the IMPA group which showed a 98.33% classification success, EfficientNet V2 for the U1-PP group with a 99% classification success, EfficientNet B0 for the interincisal angle group with a 96.67% classification success, and EfficientNet B0 for the Wits appraisal group with 98.33% accuracy. The high rates of classification accuracy observed in our study are promising, as this may eliminate the need for cephalometric analysis in most cases.

Incisor inclinations constitute one of the six keys to ideal occlusion. Incisors with ideal buccolingual inclinations not only provide good aesthetics and contribute to facial attractiveness, but also have an important functional effect [20]. In orthodontics, the position and inclination of incisors is important for a proper diagnosis and a treatment plan [21]. To our knowledge, there is no published study evaluating incisor inclinations from intraoral photographs using artificial intelligence.

Wits appraisal is a linear measurement used for evaluating the anteroposterior relationship of the maxilla and mandible. Although the intraoral photographs used in this study did not have a millimetric standardization, classification success was found to be 83.67–98.33% for this parameter. Cejudo Grano de Oro et al. evaluated angle classifications from intraoral photographs utilizing Resnet architecture and reported an accuracy of 0.63–0.64 [22].

Artificial intelligence-based software systems are significantly transforming the orthodontic field, and they constitute the cornerstone of dental applications. Artificial intelligence is applied across all aspects of orthodontics, ranging from patient interactions to diagnostic and treatment procedures [7]. Similar to this study, there are many studies that make evaluations from intraoral photographs using the deep learning method [10,23,24,25,26,27,28,29,30].

Ryu et al. evaluated crowding and extraction decisions from occlusal photographs using ResNet50, ResNet101, VGG16, and VGG19 architectures and, contrary to the results of our study, they reported VGG19 to be more successful [18]. Yu et al. evaluated the performance of the DenseNet 121 architecture for classifying cephalometric X-rays in terms of sagittal and vertical malocclusions and they found the accuracy of the Class III group to be higher than that of the Class II group [31]. Similarly, in our study, the DenseNet 121 architecture was also found to be successful in the sagittal direction evaluation made according to the Wits measurement.

ResNet is a frequently used model for intraoral photographic data in DL studies [10,27,30]. ResNet-50 is a deep convolutional neural network (CNN) model with a “Residual Network” architecture that consists of 50 layers. It uses residual connections to solve network learning and gradient disappearing problems during backpropagation. ResNet-50 learns a residual function instead of directly approximating the underlying mapping; namely, learning the difference of the output from the input [32]. Despite ResNet 101 and ResNet 152 having to be excluded from evaluation in the U1–palatal plane classifications; our study results demonstrate mostly high accuracy with the ResNet architecture. Even though the ResNet 50 model shows an 87.67% success rate in the U1–palatal plane group, it is the lowest value among the successful groups.

EfficientNet B0 and EfficientNet V2 proved to be the most successful models in our study. They have neither been used in prior studies involving intraoral photograph data nor for any dental applications.

A similar study conducted by Kocakaya et al. [33] assessed the deep learning-based grouping performance of orthodontic profile photographs and identified DenseNet 201 and EfficientNet V2 as the most successful models. Consistent with our findings, the classification performances of the VGG16 and VGG19 models were found to be the lowest. A comparison of the classification performances of the deep learning models in our study reveals that they generally exceed the classification performance of profile photographs reported in the study by Kocakaya et al. [33].

There are some limitations to our study. Successful DL models require large datasets and, for accurate classification, the dataset must be evenly distributed. Therefore, an increase in data volume may increase reliability and generalizability of results. In our study, data augmentation was performed to expand the dataset. However, a dataset containing only raw photographs without data augmentation may have prevented the attribution of success rates to enriched data. Another limitation is that the data were obtained from a single institution. Prospective approaches like federated learning, which allows model training across decentralized devices, and Zero-Shot Learning, which may serve especially in data-scarce environments, might have been employed to provide an increase in data volume [34]. Our study results should be compared to those of studies conducted with photographs in different formats, taken by different cameras, and with photographs from various institutions.

From the perspective of future studies, Vision Transformer-based models have a high potential for deep learning-based image processing. It has been reported that a Vision Transformer-based model demonstrated higher accuracy than the clinician’s judgment in diagnosing maturation of the mid-palatal suture in CBCT images [35]. Based on the attention mechanism they offer, especially the ability to overcome the limitations of CNNs, Vision Transformer-based models can learn longer-range dependencies and achieve high accuracy with fewer data. In addition, due to their scalable structure, they can perform better when trained with large datasets. In this context, it is recommended that Vision Transformer-based approaches be tested further in future studies in medical imaging.

## 5. Conclusions

This study presents promising results for the accurate estimation of cephalometric classifications using intraoral photographs, potentially eliminating the need for lateral cephalometric radiographs.

The EfficientNet B0 and EfficientNet V2 models presented the highest accuracy rates, and the VGG16 and VGG19 models presented the lowest accuracy rates.

Classifications according to the U1–palatal plane angle exhibit the lowest accuracy rates, and classifications according to the Wits appraisal exhibit the highest accuracy rates.

The utilization of different cephalometric measurements as classification parameters may yield improved accuracy rates.

## Figures and Tables

**Table 1:** Accuracy of prediction of the DL algorithm with cephalometric measurement from the intraoral photograph.

DL Model	IMPA (%)	U1-L1 (%)	U1-PP (%)	WITS (%)
DenseNet 121	95.00	95.00	96.00	97.00
DenseNet 169	96.67	96.33	96.33	96.00
DenseNet 201	97.67	95.67	96.00	98.00
EfficientNet B0	98.00	97.67	96.67 #	98.33 #
EfficientNet V2	97.67	99.00 #	94.67	97.67
Inception V3	89.00	57.00	33.67 xxx	96.67
MobileNet V2	91.33	93.67	91.67	94.67
NasNetMobile	83.67	87.00	81.00 *	83.67 *
ResNet101	90.33	92.67	36.67 xxx	93.67
ResNet152	91.00	71.00	34.33 xxx	93.67
ResNet50	92.00	89.67	87.67	95.00
VGG16	59.00 *	53.00	35.00 xxx	88.33
VGG19	60.67	51.67 *	34.67 xxx	86.67
Xception	98.33 #	97.00	33.33 xxx	91.33

# highest accuracy; * lowest accuracy; xxx not evaluated.

## Data Availability

The raw data supporting the conclusions of this article will be made available by the authors on request.

## Supplementary Materials

The following supporting information can be downloaded at: https://www.mdpi.com/article/10.3390/diagnostics15091059/s1, Figure S1. Training and Validation Loss and Training and Accuracy Graphs for MOBILENET V2; Figure S2. Confusion Matrix for Actual and Predicted IMPA values by MOBILENET V2; Figure S3. Training and Validation Loss and Training and Accuracy Graphs for INCEPTION V3; Figure S4. Confusion Matrix for Actual and Predicted IMPA values by INCEPTION V3; Figure S5. Training and Validation Loss and Training and Accuracy Graphs for DENSENET 121; Figure S6. Confusion Matrix for Actual and Predicted IMPA values by DENSENET 121; Figure S7. Training and Validation Loss and Training and Accuracy Graphs for DENSENET 169; Figure S8. Confusion Matrix for Actual and Predicted IMPA values by DENSENET 169; Figure S9. Training and Validation Loss and Training and Accuracy Graphs for DENSENET 201; Figure S10. Confusion Matrix for Actual and Predicted IMPA values by DENSENET 201; Figure S11. Training and Validation Loss and Training and Accuracy Graphs for EFFICIENTNET B0; Figure S12. Confusion Matrix for Actual and Predicted IMPA values by EFFICIENTNET B0; Figure S13. Training and Validation Loss and Training and Accuracy Graphs for XCEPTION; Figure S14. Confusion Matrix for Actual and Predicted IMPA values by XCEPTION; Figure S15. Training and Validation Loss and Training and Accuracy Graphs for VGG16; Figure S16. Confusion Matrix for Actual and Predicted IMPA values by VGG16; Figure S17. Training and Validation Loss and Training and Accuracy Graphs for VGG19; Figure S18. Confusion Matrix for Actual and Predicted IMPA values by VGG19; Figure S19. Training and Validation Loss and Training and Accuracy Graphs for NASNETMOBILE; Figure S20. Confusion Matrix for Actual and Predicted IMPA values by NASNETMOBILE; Figure S21. Training and Validation Loss and Training and Accuracy Graphs for RESNET101; Figure S22. Confusion Matrix for Actual and Predicted IMPA values by RESNET101; Figure S23. Training and Validation Loss and Training and Accuracy Graphs for RESNET152; Figure S24. Confusion Matrix for Actual and Predicted IMPA values by RESNET152; Figure S25. Training and Validation Loss and Training and Accuracy Graphs for RESNET50; Figure S26. Confusion Matrix for Actual and Predicted IMPA values by RESNET50; Figure S27. Training and Validation Loss and Training and Accuracy Graphs for EFFICIENTNET V2; Figure S28. Confusion Matrix for Actual and Predicted IMPA values by EFFICIENTNET V2; Figure S29. Training and Validation Loss and Training and Accuracy Graphs for MOBILENET V2; Figure S30. Confusion Matrix for Actual and Predicted U1-L1 values by MOBILENET V2; Figure S31. Training and Validation Loss and Training and Accuracy Graphs for INCEPTION V3; Figure S32. Confusion Matrix for Actual and Predicted U1-L1 values by INCEPTION V3; Figure S33. Training and Validation Loss and Training and Accuracy Graphs for DENSENET 121; Figure S34. Confusion Matrix for Actual and Predicted U1-L1 values by DENSENET 121; Figure S35. Training and Validation Loss and Training and Accuracy Graphs for DENSENET 169; Figure S36. Confusion Matrix for Actual and Predicted U1-L1 values by DENSENET 169; Figure S37. Training and Validation Loss and Training and Accuracy Graphs for DENSENET 201; Figure S38. Confusion Matrix for Actual and Predicted U1-L1 values by DENSENET 201; Figure S39. Training and Validation Loss and Training and Accuracy Graphs for EFFICIENTNET B0; Figure S40. Confusion Matrix for Actual and Predicted U1-L1 values by EFFICIENTNET B0; Figure S41. Training and Validation Loss and Training and Accuracy Graphs for XCEPTION; Figure S42. Confusion Matrix for Actual and Predicted U1-L1 values by XCEPTION; Figure S43. Training and Validation Loss and Training and Accuracy Graphs for VGG16; Figure S44. Confusion Matrix for Actual and Predicted U1-L1 values by VGG16; Figure S45. Training and Validation Loss and Training and Accuracy Graphs for VGG19; Figure S46. Confusion Matrix for Actual and Predicted U1-L1 values by VGG19; Figure S47. Training and Validation Loss and Training and Accuracy Graphs for NASNETMOBILE; Figure S48. Confusion Matrix for Actual and Predicted U1-L1 values by NASNETMOBILE; Figure S49. Training and Validation Loss and Training and Accuracy Graphs for RESNET101; Figure S50. Confusion Matrix for Actual and Predicted U1-L1 values by RESNET101; Figure S51. Training and Validation Loss and Training and Accuracy Graphs for RESNET152; Figure S52. Confusion Matrix for Actual and Predicted U1-L1 values by RESNET152; Figure S53. Training and Validation Loss and Training and Accuracy Graphs for RESNET50; Figure S54. Confusion Matrix for Actual and Predicted U1-L1 values by RESNET50; Figure S55. Training and Validation Loss and Training and Accuracy Graphs for EFFICIENTNET V2; Figure S56. Confusion Matrix for Actual and Predicted U1-L1 values by EFFICIENTNET V2; Figure S57. Training and Validation Loss and Training and Accuracy Graphs for MOBILENET V2; Figure S58. Confusion Matrix for Actual and Predicted U1-PP values by MOBILENET V2; Figure S59. Training and Validation Loss and Training and Accuracy Graphs for DENSENET 121; Figure S60. Confusion Matrix for Actual and Predicted U1-PP values by DENSENET 121; Figure S61. Training and Validation Loss and Training and Accuracy Graphs for DENSENET 169; Figure S62. Confusion Matrix for Actual and Predicted U1-PP values by DENSENET 169; Figure S63. Training and Validation Loss and Training and Accuracy Graphs for DENSENET 201; Figure S64. Confusion Matrix for Actual and Predicted U1-PP values by DENSENET 201; Figure S65. Training and Validation Loss and Training and Accuracy Graphs for EFFICIENTNET B0; Figure S66. Confusion Matrix for Actual and Predicted U1-PP values by EFFICIENTNET B0; Figure S67. Training and Validation Loss and Training and Accuracy Graphs for NASNETMOBILE; Figure S68. Confusion Matrix for Actual and Predicted U1-PP values by NASNETMOBILE; Figure S69. Training and Validation Loss and Training and Accuracy Graphs for RESNET50; Figure S70. Confusion Matrix for Actual and Predicted U1-PP values by RESNET50; Figure S71. Training and Validation Loss and Training and Accuracy Graphs for EFFICIENTNET V2; Figure S72. Confusion Matrix for Actual and Predicted U1-PP values by EFFICIENTNET V2; Figure S73. Training and Validation Loss and Training and Accuracy Graphs for MOBILENET V2; Figure S74. Confusion Matrix for Actual and Predicted WITS values by MOBILENET V2; Figure S75. Training and Validation Loss and Training and Accuracy Graphs for INCEPTION V3; Figure S76. Confusion Matrix for Actual and Predicted WITS values by INCEPTION V3; Figure S77. Training and Validation Loss and Training and Accuracy Graphs for DENSENET 121; Figure S78. Confusion Matrix for Actual and Predicted WITS values by DENSENET 121; Figure S79. Training and Validation Loss and Training and Accuracy Graphs for DENSENET 169; Figure S80. Confusion Matrix for Actual and Predicted WITS values by DENSENET 169; Figure S81. Training and Validation Loss and Training and Accuracy Graphs for DENSENET 201; Figure S82. Confusion Matrix for Actual and Predicted WITS values by DENSENET 201; Figure S83. Training and Validation Loss and Training and Accuracy Graphs for EFFICIENTNET B0; Figure S84. Confusion Matrix for Actual and Predicted WITS values by EFFICIENTNET B0; Figure S85. Training and Validation Loss and Training and Accuracy Graphs for XCEPTION; Figure S86. Confusion Matrix for Actual and Predicted WITS values by XCEPTION; Figure S87. Training and Validation Loss and Training and Accuracy Graphs for VGG16; Figure S88. Confusion Matrix for Actual and Predicted WITS values by VGG16; Figure S89. Training and Validation Loss and Training and Accuracy Graphs for VGG19; Figure S90. Confusion Matrix for Actual and Predicted WITS values by VGG19; Figure S91. Training and Validation Loss and Training and Accuracy Graphs for NASNETMOBILE; Figure S92. Confusion Matrix for Actual and Predicted WITS values by NASNETMOBILE; Figure S93. Training and Validation Loss and Training and Accuracy Graphs for RESNET101; Figure S94. Confusion Matrix for Actual and Predicted WITS values by RESNET101; Figure S95. Training and Validation Loss and Training and Accuracy Graphs for RESNET152; Figure S96. Confusion Matrix for Actual and Predicted WITS values by RESNET152; Figure S97. Training and Validation Loss and Training and Accuracy Graphs for RESNET50; Figure S98. Confusion Matrix for Actual and Predicted WITS values by RESNET50; Figure S99. Training and Validation Loss and Training and Accuracy Graphs for EFFICIENTNET V2; Figure S100. Confusion Matrix for Actual and Predicted WITS values by EFFICIENTNET V2; Table S1. Summary table for accuracy values of IMPA Classification; Table S2. Classification Report for IMPA by MOBILENET V2; Table S3. Classification Report for IMPA by INCEPTION V3; Table S4. Classification Report for IMPA by DENSENET 121; Table S5. Classification Report for IMPA by DENSENET 169; Table S6. Classification Report for IMPA by DENSENET 201; Table S7. Classification Report for IMPA by EFFICIENTNET B0; Table S8. Classification Report for IMPA by XCEPTION; Table S9. Classification Report for IMPA by VGG16; Table S10. Classification Report for IMPA by VGG19; Table S11. Classification Report for IMPA by NASNETMOBILE; Table S12. Classification Report for IMPA by RESNET101; Table S13. Classification Report for IMPA by RESNET152; Table S14. Classification Report for IMPA by RESNET50; Table S15. Classification Report for IMPA by EFFICIENTNET V2; Table S16. Summary table for accuracy values of U1-L1 Classification; Table S17. Classification Report for U1-L1 by MOBILENET V2; Table S18. Classification Report for U1-L1 by INCEPTION V3; Table S19. Classification Report for U1-L1 by DENSENET 121; Table S20. Classification Report for U1-L1 by DENSENET 169; Table S21. Classification Report for U1-L1 by DENSENET 201; Table S22. Classification Report for U1-L1 by EFFICIENTNET B0; Table S23. Classification Report for U1-L1 by XCEPTION; Table S24. Classification Report for U1-L1 by VGG16; Table S25. Classification Report for U1-L1 by VGG19; Table S26. Classification Report for U1-L1 by NASNETMOBILE; Table S27. Classification Report for U1-L1 by RESNET101; Table S28. Classification Report for U1-L1 by RESNET152; Table S29. Classification Report for U1-L1 by RESNET50; Table S30. Classification Report for U1-L1 by EFFICIENTNET V2; Table S31. Summary table for accuracy values of U1-PP Classification; Table S32. Classification Report for U1-PP by MOBILENET V2; Table S33. Classification Report for U1-PP by DENSENET 121; Table S34. Classification Report for U1-PP by DENSENET 169; Table S35. Classification Report for U1-PP by DENSENET 201; Table S36. Classification Report for U1-PP by EFFICIENTNET B0; Table S37. Classification Report for U1-PP by NASNETMOBILE; Table S38. Classification Report for U1-PP by RESNET50; Table S39. Classification Report for U1-PP by EFFICIENTNET V2; Table S40. Summary table for accuracy values of WITS Classification; Table S41. Classification Report for WITS by MOBILENET V2; Table S42. Classification Report for WITS by INCEPTION V3; Table S43. Classification Report for WITS by DENSENET 121; Table S44. Classification Report for WITS by DENSENET 169; Table S45. Classification Report for WITS by DENSENET 201; Table S46. Classification Report for WITS by EFFICIENTNET B0; Table S47. Classification Report for WITS by XCEPTION; Table S48. Classification Report for WITS by VGG16; Table S49. Classification Report for WITS by VGG19; Table S50. Classification Report for WITS by NASNETMOBILE; Table S51. Classification Report for WITS by RESNET101; Table S52. Classification Report for WITS by RESNET152; Table S53. Classification Report for WITS by RESNET50; Table S54. Classification Report for WITS by EFFICIENTNET V2.

## Abbreviations

The following abbreviations are used in this manuscript:

AI	Artificial intelligence
DL	Deep learning
CNN	Convolutional neural network
ML	Machine learning
