# Supplementary material for: Classification of Intraoral Photographs with Deep Learning Algorithms Trained According to Cephalometric Measurements
Source: Diagnostics (Basel). 2025 Apr 22;15(9):1059. doi: 10.3390/diagnostics15091059 (PMC12071284; doi:10.3390/diagnostics15091059)
Supplement: Supplementary file 1 [file diagnostics-15-01059-s001.zip › diagnostics-3519690-supplementary.pdf]

LIST OF THE CAPTIONS/LEGENDS

Table S1. Summary table for accuracy values of IMPA Classification

Figure S1. Training and Validation Loss and Training and Accuracy Graphs for MOBILENET V2

Figure S2. Confusion Matrix for Actual and Predicted IMPA values by MOBILENET V2

Table S2. Classification Report for IMPA by MOBILENET V2

Figure S3. Training and Validation Loss and Training and Accuracy Graphs for INCEPTION V3

Figure S4. Confusion Matrix for Actual and Predicted IMPA values by INCEPTION V3

Table S3. Classification Report for IMPA by INCEPTION V3

Figure S5. Training and Validation Loss and Training and Accuracy Graphs for DENSENET 121

Figure S6. Confusion Matrix for Actual and Predicted IMPA values by DENSENET 121

Table S4. Classification Report for IMPA by DENSENET 121

Figure S7. Training and Validation Loss and Training and Accuracy Graphs for DENSENET 169

Figure S8. Confusion Matrix for Actual and Predicted IMPA values by DENSENET 169

Table S5. Classification Report for IMPA by DENSENET 169

Figure S9. Training and Validation Loss and Training and Accuracy Graphs for DENSENET 201

Figure S10. Confusion Matrix for Actual and Predicted IMPA values by DENSENET 201

Table S6. Classification Report for IMPA by DENSENET 201

Figure S11. Training and Validation Loss and Training and Accuracy Graphs for EFFICIENTNET B0

Figure S12. Confusion Matrix for Actual and Predicted IMPA values by EFFICIENTNET B0

Table S7. Classification Report for IMPA by EFFICIENTNET B0

Figure S13. Training and Validation Loss and Training and Accuracy Graphs for XCEPTION

Figure S14. Confusion Matrix for Actual and Predicted IMPA values by XCEPTION

Table S8. Classification Report for IMPA by XCEPTION

Figure S15. Training and Validation Loss and Training and Accuracy Graphs for VGG16

Figure S16. Confusion Matrix for Actual and Predicted IMPA values by VGG16

Table S9. Classification Report for IMPA by VGG16

Figure S17. Training and Validation Loss and Training and Accuracy Graphs for VGG19

Figure S18. Confusion Matrix for Actual and Predicted IMPA values by VGG19

Table S10. Classification Report for IMPA by VGG19

Figure S19. Training and Validation Loss and Training and Accuracy Graphs for NASNETMOBILE

Figure S20. Confusion Matrix for Actual and Predicted IMPA values by NASNETMOBILE

Table S11. Classification Report for IMPA by NASNETMOBILE

Figure S21. Training and Validation Loss and Training and Accuracy Graphs for RESNET101

Figure S22. Confusion Matrix for Actual and Predicted IMPA values by RESNET101

Table S12. Classification Report for IMPA by RESNET101

Figure S23. Training and Validation Loss and Training and Accuracy Graphs for RESNET152

Figure S24. Confusion Matrix for Actual and Predicted IMPA values by RESNET152

Table S13. Classification Report for IMPA by RESNET152

Figure S25. Training and Validation Loss and Training and Accuracy Graphs for RESNET50

Figure S26. Confusion Matrix for Actual and Predicted IMPA values by RESNET50

Table S14. Classification Report for IMPA by RESNET50

Figure S27. Training and Validation Loss and Training and Accuracy Graphs for EFFICIENTNET V2

Figure S28. Confusion Matrix for Actual and Predicted IMPA values by EFFICIENTNET V2

Table S15. Classification Report for IMPA by EFFICIENTNET V2

Table S16. Summary table for accuracy values of U1-L1 Classification

Figure S29. Training and Validation Loss and Training and Accuracy Graphs for MOBILENET V2

Figure S30. Confusion Matrix for Actual and Predicted U1-L1 values by MOBILENET V2

Table S17. Classification Report for U1-L1 by MOBILENET V2

Figure S31. Training and Validation Loss and Training and Accuracy Graphs for INCEPTION V3

Figure S32. Confusion Matrix for Actual and Predicted U1-L1 values by INCEPTION V3

Table S18. Classification Report for U1-L1 by INCEPTION V3

Figure S33. Training and Validation Loss and Training and Accuracy Graphs for DENSENET 121

Figure S34. Confusion Matrix for Actual and Predicted U1-L1 values by DENSENET 121

Table S19. Classification Report for U1-L1 by DENSENET 121

Figure S35. Training and Validation Loss and Training and Accuracy Graphs for DENSENET 169

Figure S36. Confusion Matrix for Actual and Predicted U1-L1 values by DENSENET 169

Table S20. Classification Report for U1-L1 by DENSENET 169

Figure S37. Training and Validation Loss and Training and Accuracy Graphs for DENSENET 201

Figure S38. Confusion Matrix for Actual and Predicted U1-L1 values by DENSENET 201

Table S21. Classification Report for U1-L1 by DENSENET 201

Figure S39. Training and Validation Loss and Training and Accuracy Graphs for EFFICIENTNET B0

Figure S40. Confusion Matrix for Actual and Predicted U1-L1 values by EFFICIENTNET B0

Table S22. Classification Report for U1-L1 by EFFICIENTNET B0

Figure S41. Training and Validation Loss and Training and Accuracy Graphs for XCEPTION

Figure S42. Confusion Matrix for Actual and Predicted U1-L1 values by XCEPTION

Table S23. Classification Report for U1-L1 by XCEPTION

Figure S43. Training and Validation Loss and Training and Accuracy Graphs for VGG16

Figure S44. Confusion Matrix for Actual and Predicted U1-L1 values by VGG16

Table S24. Classification Report for U1-L1 by VGG16

Figure S45. Training and Validation Loss and Training and Accuracy Graphs for VGG19

Figure S46. Confusion Matrix for Actual and Predicted U1-L1 values by VGG19

Table S25. Classification Report for U1-L1 by VGG19

Figure S47. Training and Validation Loss and Training and Accuracy Graphs for NASNETMOBILE

Figure S48. Confusion Matrix for Actual and Predicted U1-L1 values by NASNETMOBILE

Table S26. Classification Report for U1-L1 by NASNETMOBILE

Figure S49. Training and Validation Loss and Training and Accuracy Graphs for RESNET101

Figure S50. Confusion Matrix for Actual and Predicted U1-L1 values by RESNET101

Table S27. Classification Report for U1-L1 by RESNET101

Figure S51. Training and Validation Loss and Training and Accuracy Graphs for RESNET152

Figure S52. Confusion Matrix for Actual and Predicted U1-L1 values by RESNET152

Table S28. Classification Report for U1-L1 by RESNET152

Figure S53. Training and Validation Loss and Training and Accuracy Graphs for RESNET50

Figure S54. Confusion Matrix for Actual and Predicted U1-L1 values by RESNET50

Table S29. Classification Report for U1-L1 by RESNET50

Figure S55. Training and Validation Loss and Training and Accuracy Graphs for EFFICIENTNET V2

Figure S56. Confusion Matrix for Actual and Predicted U1-L1 values by EFFICIENTNET V2

Table S30. Classification Report for U1-L1 by EFFICIENTNET V2

Table S31. Summary table for accuracy values of U1-PP Classification

Figure S57. Training and Validation Loss and Training and Accuracy Graphs for MOBILENET V2

Figure S58. Confusion Matrix for Actual and Predicted U1-PP values by MOBILENET V2

Table S32. Classification Report for U1-PP by MOBILENET V2

Figure S59. Training and Validation Loss and Training and Accuracy Graphs for DENSENET 121

Figure S60. Confusion Matrix for Actual and Predicted U1-PP values by DENSENET 121

Table S33. Classification Report for U1-PP by DENSENET 121

Figure S61. Training and Validation Loss and Training and Accuracy Graphs for DENSENET 169

Figure S62. Confusion Matrix for Actual and Predicted U1-PP values by DENSENET 169

Table S34. Classification Report for U1-PP by DENSENET 169

Figure S63. Training and Validation Loss and Training and Accuracy Graphs for DENSENET 201

Figure S64. Confusion Matrix for Actual and Predicted U1-PP values by DENSENET 201

Table S35. Classification Report for U1-PP by DENSENET 201

Figure S65. Training and Validation Loss and Training and Accuracy Graphs for EFFICIENTNET B0

Figure S66. Confusion Matrix for Actual and Predicted U1-PP values by EFFICIENTNET B0

Table S36. Classification Report for U1-PP by EFFICIENTNET B0

Figure S67. Training and Validation Loss and Training and Accuracy Graphs for NASNETMOBILE

Figure S68. Confusion Matrix for Actual and Predicted U1-PP values by NASNETMOBILE

Table S37. Classification Report for U1-PP by NASNETMOBILE

Figure S69. Training and Validation Loss and Training and Accuracy Graphs for RESNET50

Figure S70. Confusion Matrix for Actual and Predicted U1-PP values by RESNET50

Table S38. Classification Report for U1-PP by RESNET50

Figure S71. Training and Validation Loss and Training and Accuracy Graphs for EFFICIENTNET V2

Figure S72. Confusion Matrix for Actual and Predicted U1-PP values by EFFICIENTNET V2

Table S39. Classification Report for U1-PP by EFFICIENTNET V2

Table S40. Summary table for accuracy values of WITS Classification

Figure S73. Training and Validation Loss and Training and Accuracy Graphs for MOBILENET V2

Figure S74. Confusion Matrix for Actual and Predicted WITS values by MOBILENET V2

Table S41. Classification Report for WITS by MOBILENET V2

Figure S75. Training and Validation Loss and Training and Accuracy Graphs for INCEPTION V3

Figure S76. Confusion Matrix for Actual and Predicted WITS values by INCEPTION V3

Table S42. Classification Report for WITS by INCEPTION V3

Figure S77. Training and Validation Loss and Training and Accuracy Graphs for DENSENET 121

Figure S78. Confusion Matrix for Actual and Predicted WITS values by DENSENET 121

Table S43. Classification Report for WITS by DENSENET 121

Figure S79. Training and Validation Loss and Training and Accuracy Graphs for DENSENET 169

Figure S80. Confusion Matrix for Actual and Predicted WITS values by DENSENET 169

Table S44. Classification Report for WITS by DENSENET 169

Figure S81. Training and Validation Loss and Training and Accuracy Graphs for DENSENET 201

Figure S82. Confusion Matrix for Actual and Predicted WITS values by DENSENET 201

Table S45. Classification Report for WITS by DENSENET 201

Figure S83. Training and Validation Loss and Training and Accuracy Graphs for EFFICIENTNET B0

Figure S84. Confusion Matrix for Actual and Predicted WITS values by EFFICIENTNET B0

Table S46. Classification Report for WITS by EFFICIENTNET B0

Figure S85. Training and Validation Loss and Training and Accuracy Graphs for XCEPTION

Figure S86. Confusion Matrix for Actual and Predicted WITS values by XCEPTION

Table S47. Classification Report for WITS by XCEPTION

Figure S87. Training and Validation Loss and Training and Accuracy Graphs for VGG16

Figure S88. Confusion Matrix for Actual and Predicted WITS values by VGG16

Table S48. Classification Report for WITS by VGG16

Figure S89. Training and Validation Loss and Training and Accuracy Graphs for VGG19

Figure S90. Confusion Matrix for Actual and Predicted WITS values by VGG19

Table S49. Classification Report for WITS by VGG19

Figure S91. Training and Validation Loss and Training and Accuracy Graphs for NASNETMOBILE

Figure S92. Confusion Matrix for Actual and Predicted WITS values by NASNETMOBILE

Table S50. Classification Report for WITS by NASNETMOBILE

Figure S93. Training and Validation Loss and Training and Accuracy Graphs for RESNET101

Figure S94. Confusion Matrix for Actual and Predicted WITS values by RESNET101

Table S51. Classification Report for WITS by RESNET101

Figure S95. Training and Validation Loss and Training and Accuracy Graphs for RESNET152

Figure S96. Confusion Matrix for Actual and Predicted WITS values by RESNET152

Table S52. Classification Report for WITS by RESNET152

Figure S97. Training and Validation Loss and Training and Accuracy Graphs for RESNET50

Figure S98. Confusion Matrix for Actual and Predicted WITS values by RESNET50

Table S53. Classification Report for WITS by RESNET50

Figure S99. Training and Validation Loss and Training and Accuracy Graphs for EFFICIENTNET V2

Figure S100. Confusion Matrix for Actual and Predicted WITS values by EFFICIENTNET V2

Table S54. Classification Report for WITS by EFFICIENTNET V2

Table S1. Summary table for accuracy values of IMPA

IMPA	
MOBILENET V2	91.33
INCEPTION V3	89.00
DENSENET 121	95.00
DENSENET 169	96.67
DENSENET 201	97.67
EFFICIENTNET B0	98.00
XCEPTION	98.33
VGG16	59.00
VGG19	60.67
NASNETMOBILE	83.67
RESNET101	90.33 (GRAPH)
RESNET152	91.00 (GRAPH)
RESNET50	92.00 (GRAPH)
EFFICIENTNET V2	97.67

Figure S1. Training and Validation Loss and Training and Accuracy Graphs for MOBILENET V2

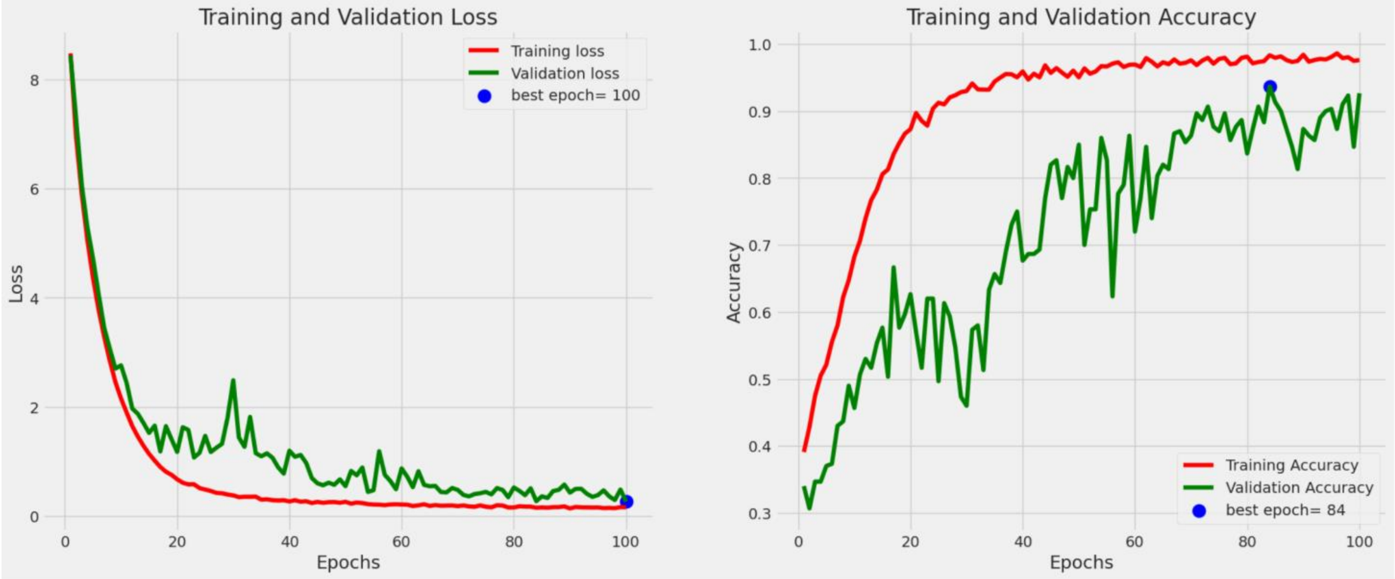

Figure S2. Confusion Matrix for Actual and Predicted IMPA values.

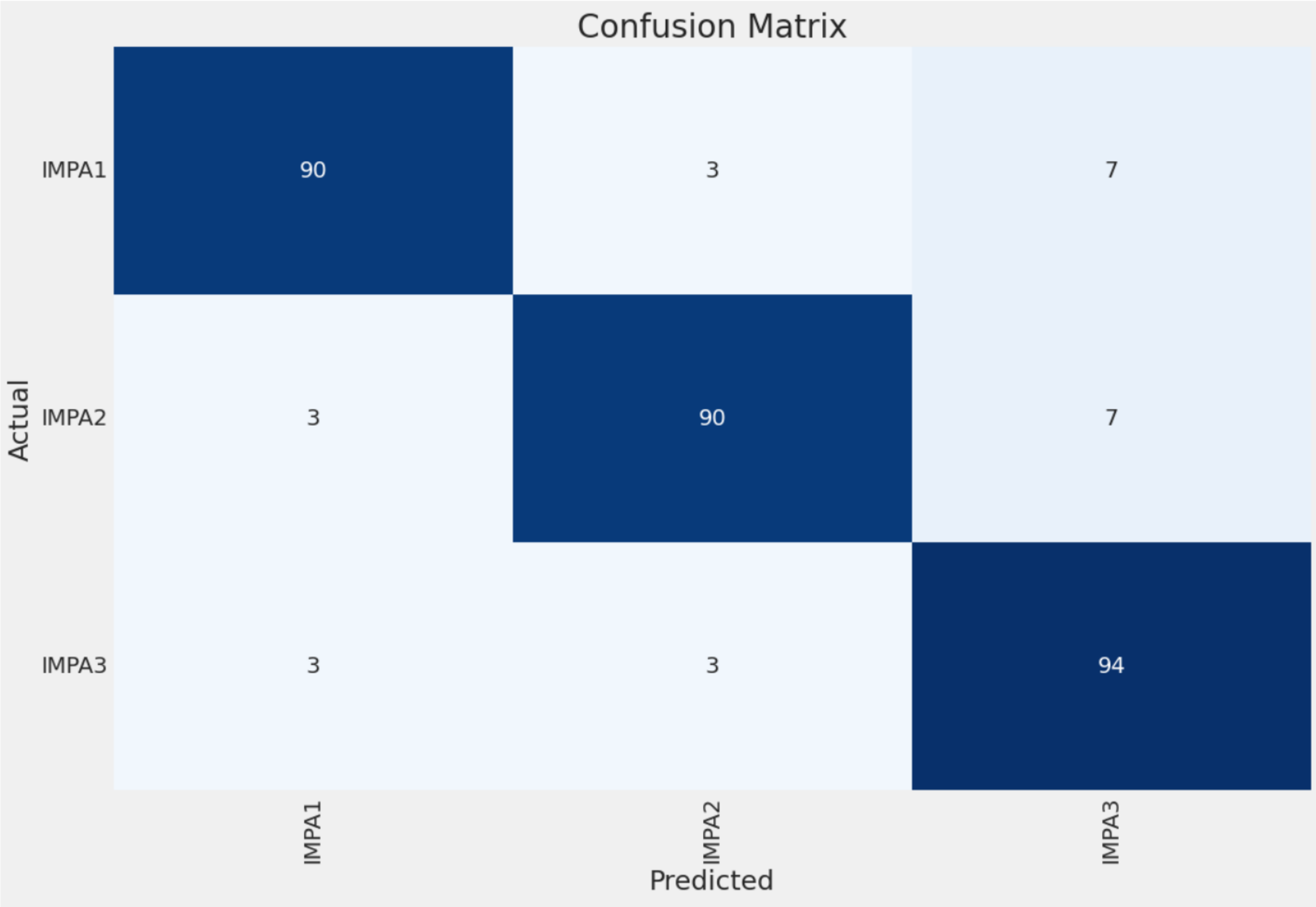

Table S2. Classification Report for IMPA

	precision	recall	f1-score	support
IMPA1	0.9375	0.9000	0.9184	100
IMPA2	0.9375	0.9000	0.9184	100
IMPA3	0.8704	0.9400	0.9038	100
accuracy			0.9133	300
macro avg	0.9151	0.9133	0.9135	300
weighted avg	0.9151	0.9133	0.9135	300

Figure S3. Training and Validation Loss and Training and Accuracy Graphs for INCEPTION V3

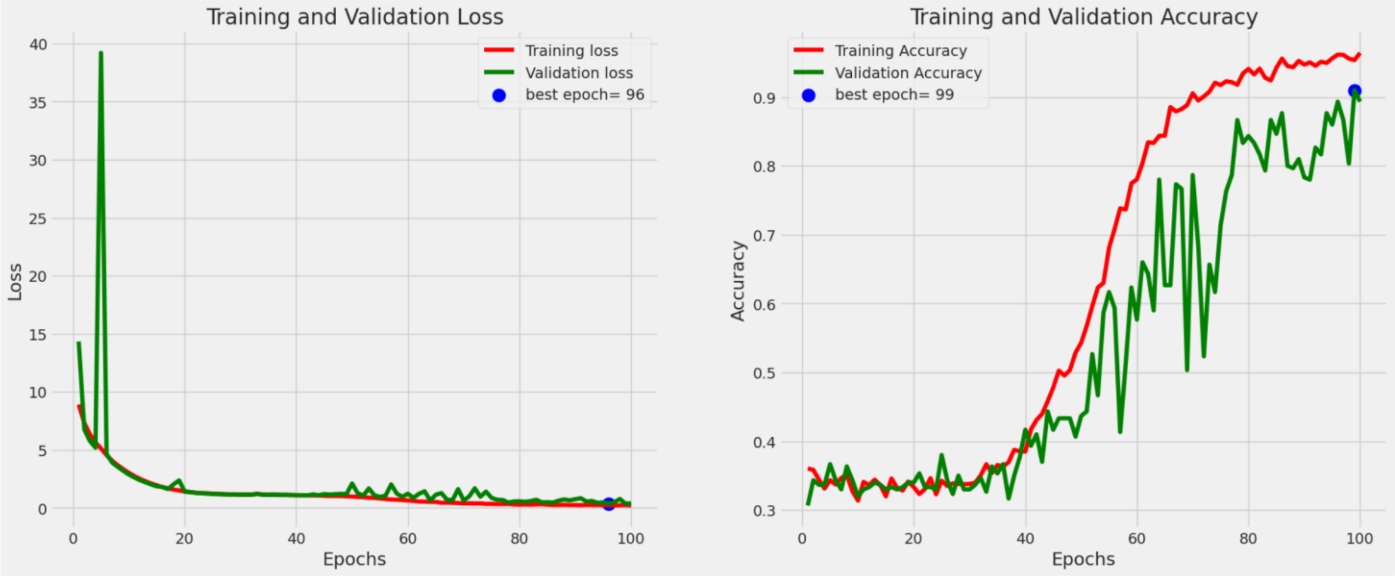

Figure S4. Confusion Matrix for Actual and Predicted IMPA values by INCEPTION V3

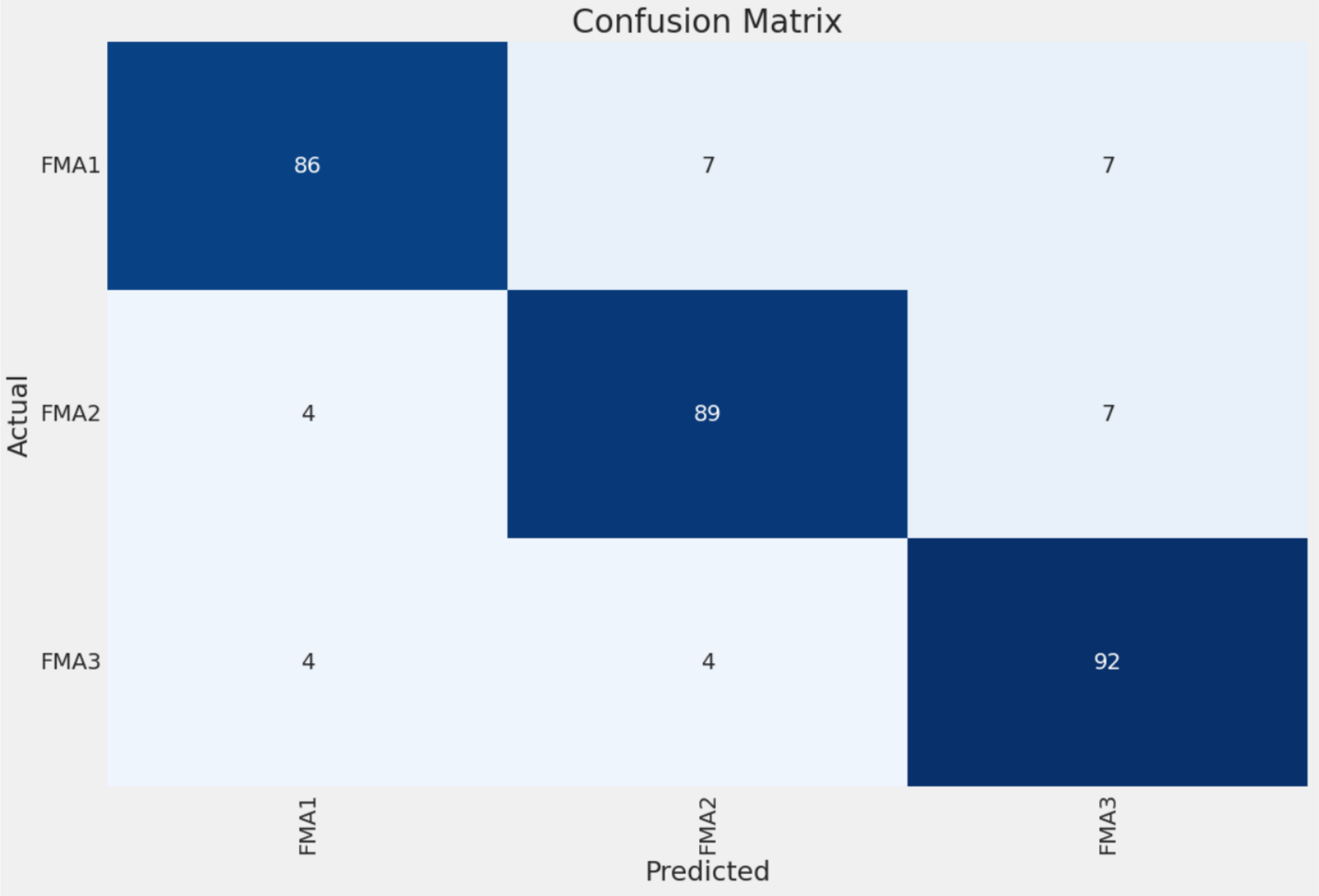

Table S3. Classification Report for IMPA by INCEPTION V3

----

	precision	recall	f1-score	support
FMA1	0.9149	0.8600	0.8866	100
FMA2	0.8900	0.8900	0.8900	100
FMA3	0.8679	0.9200	0.8932	100
accuracy			0.8900	300
macro avg	0.8909	0.8900	0.8899	300
weighted avg	0.8909	0.8900	0.8899	300

Figure S5. Training and Validation Loss and Training and Accuracy Graphs for DENSENET 121

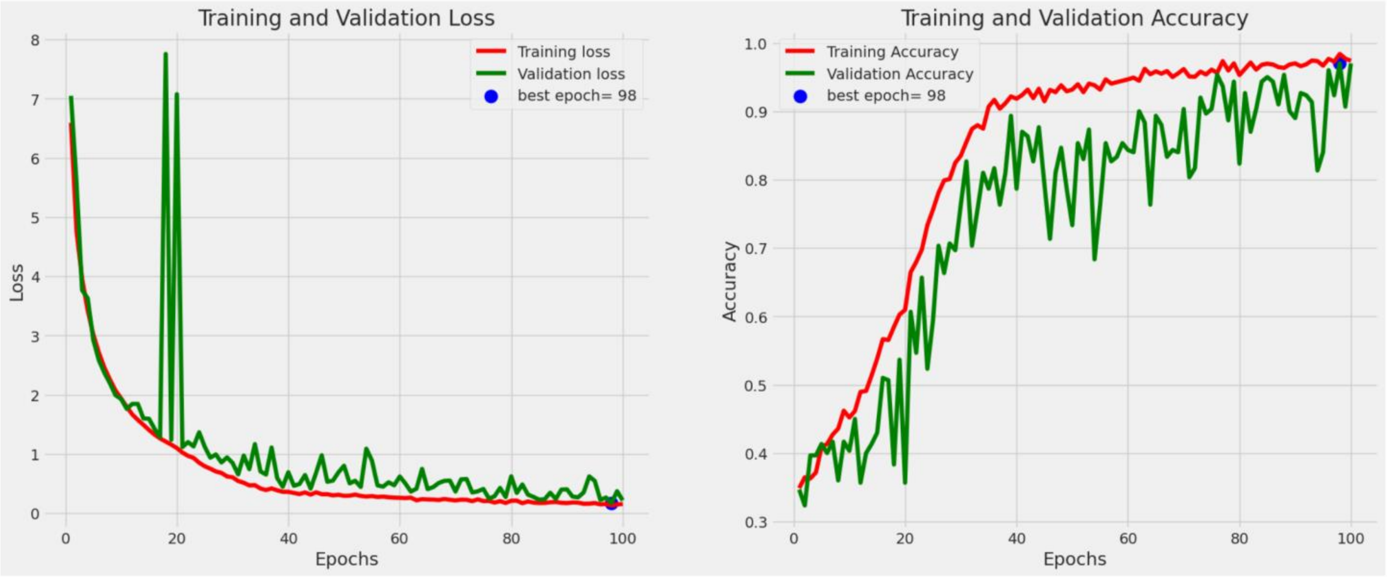

Figure S6. Confusion Matrix for Actual and Predicted IMPA values by DENSENET 121

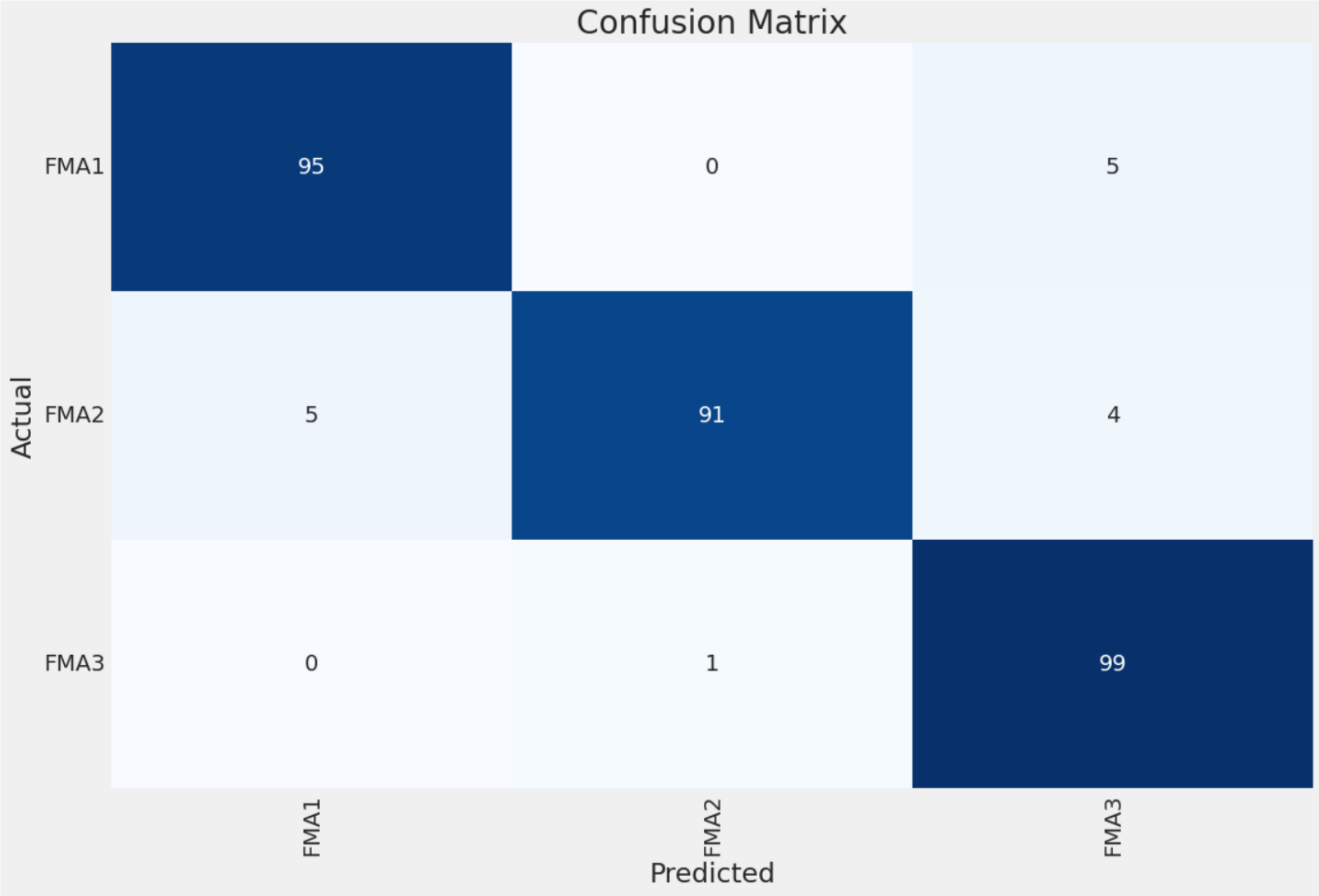

Table S4. Classification Report for IMPA by DENSENET 121

----

	precision	recall	f1-score	support
FMA1	0.9500	0.9500	0.9500	100
FMA2	0.9891	0.9100	0.9479	100
FMA3	0.9167	0.9900	0.9519	100
accuracy			0.9500	300
macro avg	0.9519	0.9500	0.9499	300
weighted avg	0.9519	0.9500	0.9499	300

Figure S7. Training and Validation Loss and Training and Accuracy Graphs for DENSENET 169

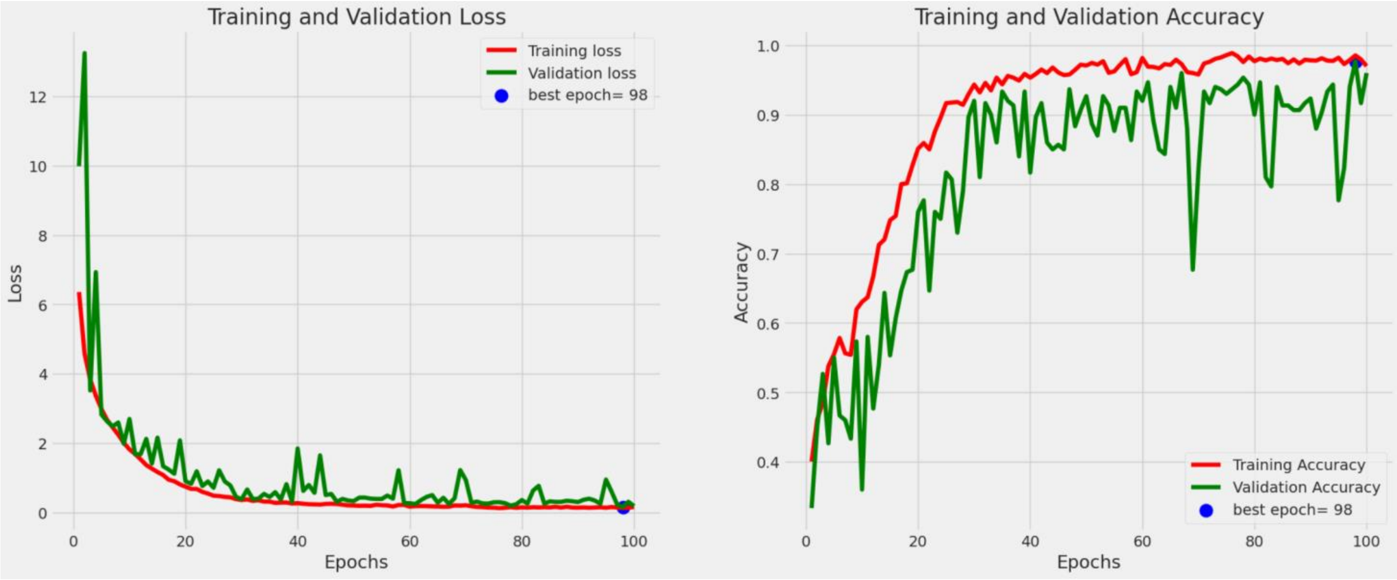

Figure S8. Confusion Matrix for Actual and Predicted IMPA values by DENSENET 169

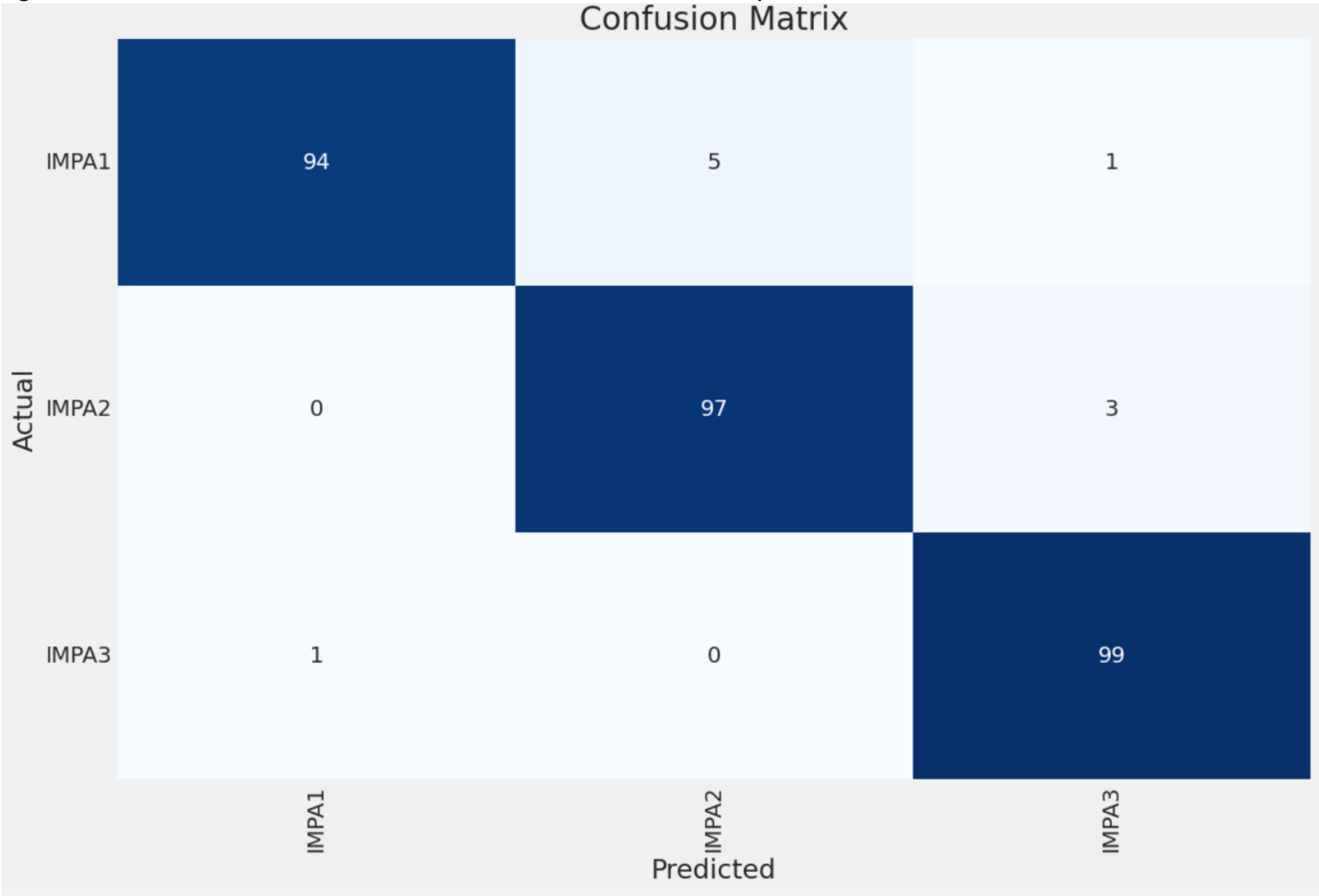

Table S5. Classification Report for IMPA by DENSENET 169

----

	precision	recall	f1-score	support
IMPA1	0.9895	0.9400	0.9641	100
IMPA2	0.9510	0.9700	0.9604	100
IMPA3	0.9612	0.9900	0.9754	100

accuracy			0.9667	300
macro avg	0.9672	0.9667	0.9666	300
weighted avg	0.9672	0.9667	0.9666	300

Figure S9. Training and Validation Loss and Training and Accuracy Graphs for DENSENET 201

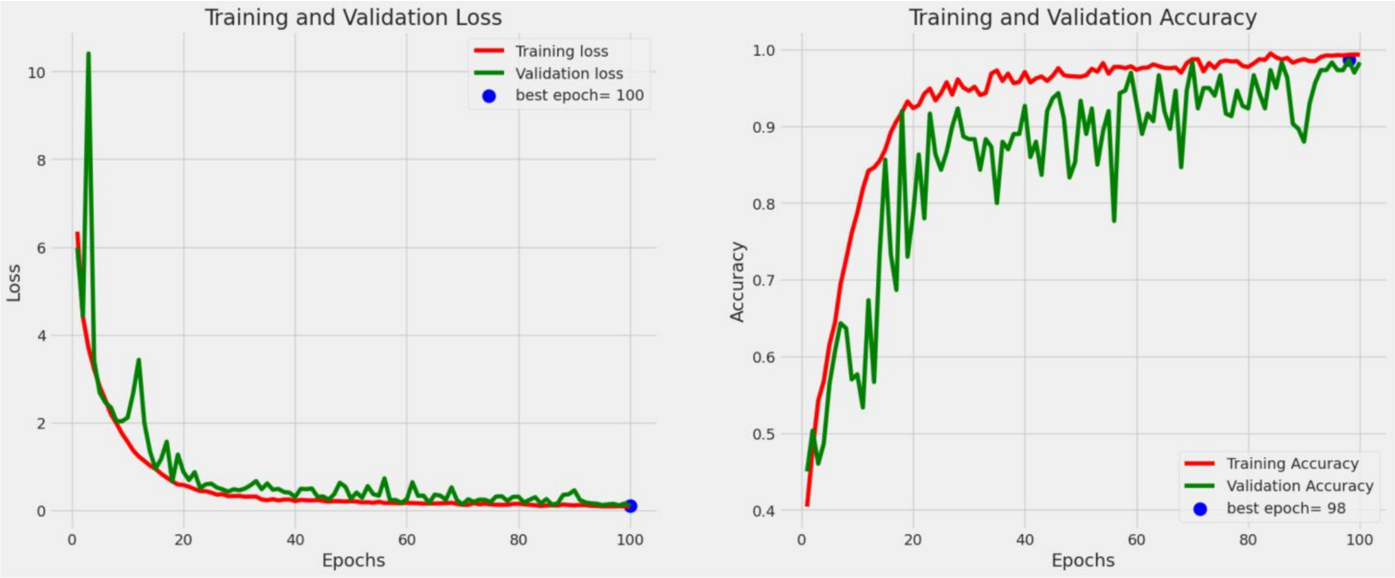

Figure S10. Confusion Matrix for Actual and Predicted IMPA values by DENSENET 201

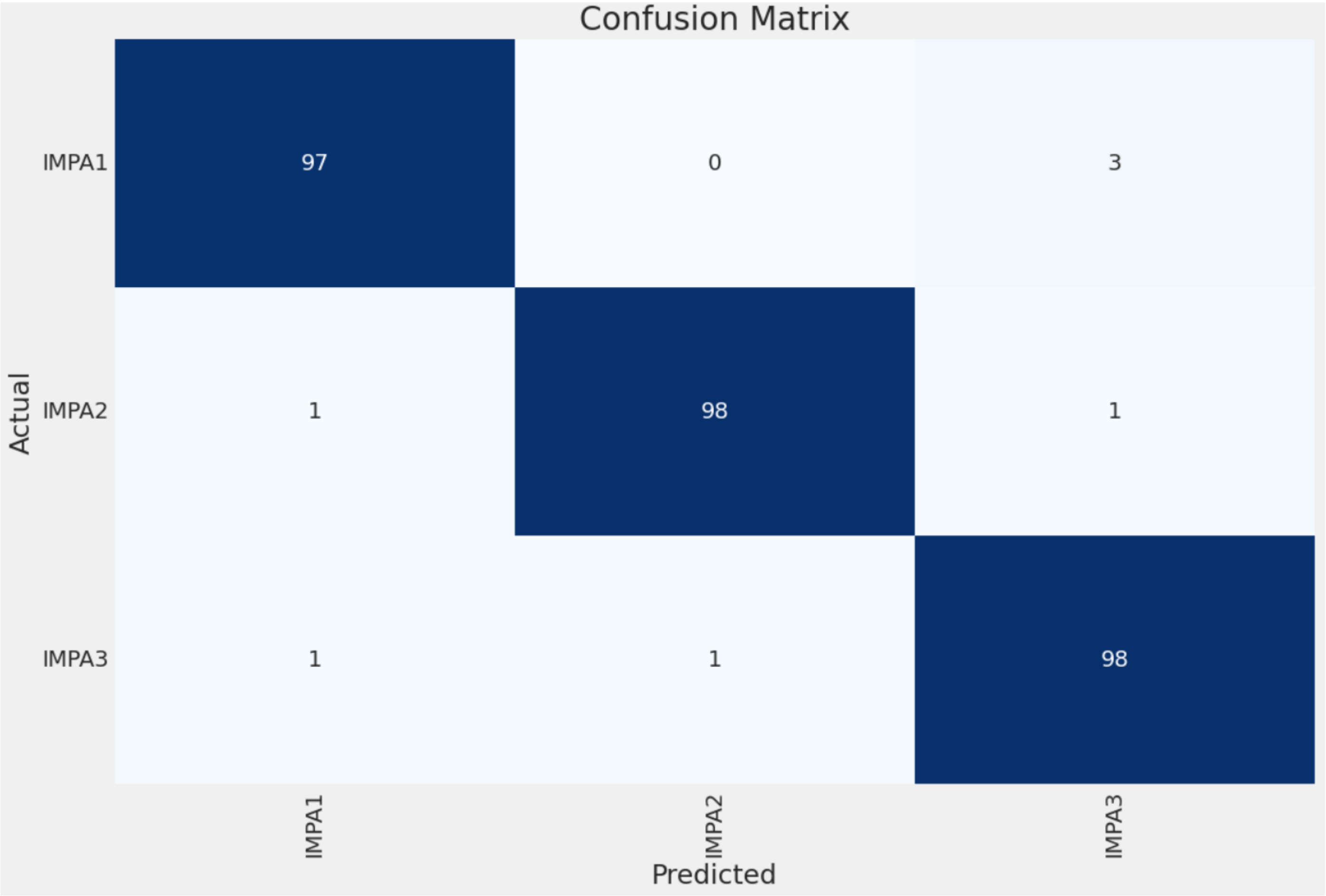

Table S6. Classification Report for IMPA by DENSENET 201

----

	precision	recall	f1-score	support
IMPA1	0.9798	0.9700	0.9749	100
IMPA2	0.9899	0.9800	0.9849	100
IMPA3	0.9608	0.9800	0.9703	100
accuracy			0.9767	300
macro avg	0.9768	0.9767	0.9767	300
weighted avg	0.9768	0.9767	0.9767	300

Figure S11. Training and Validation Loss and Training and Accuracy Graphs for EFFICIENTNET B0

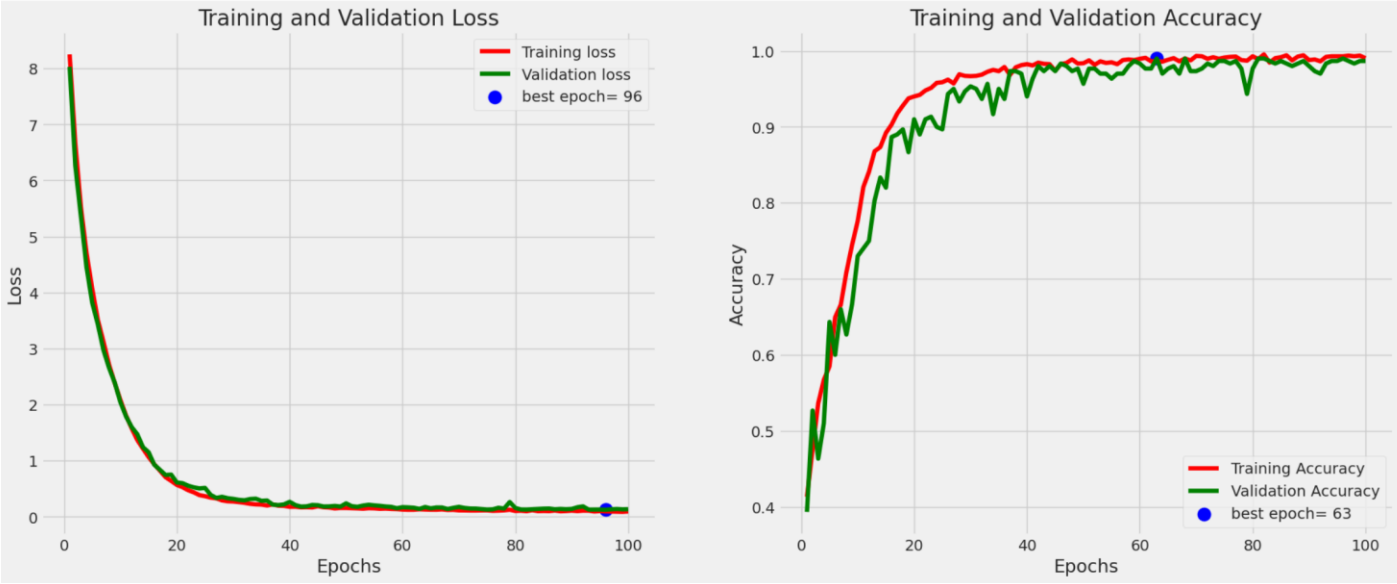

Figure S12. Confusion Matrix for Actual and Predicted IMPA values by EFFICIENTNET B0

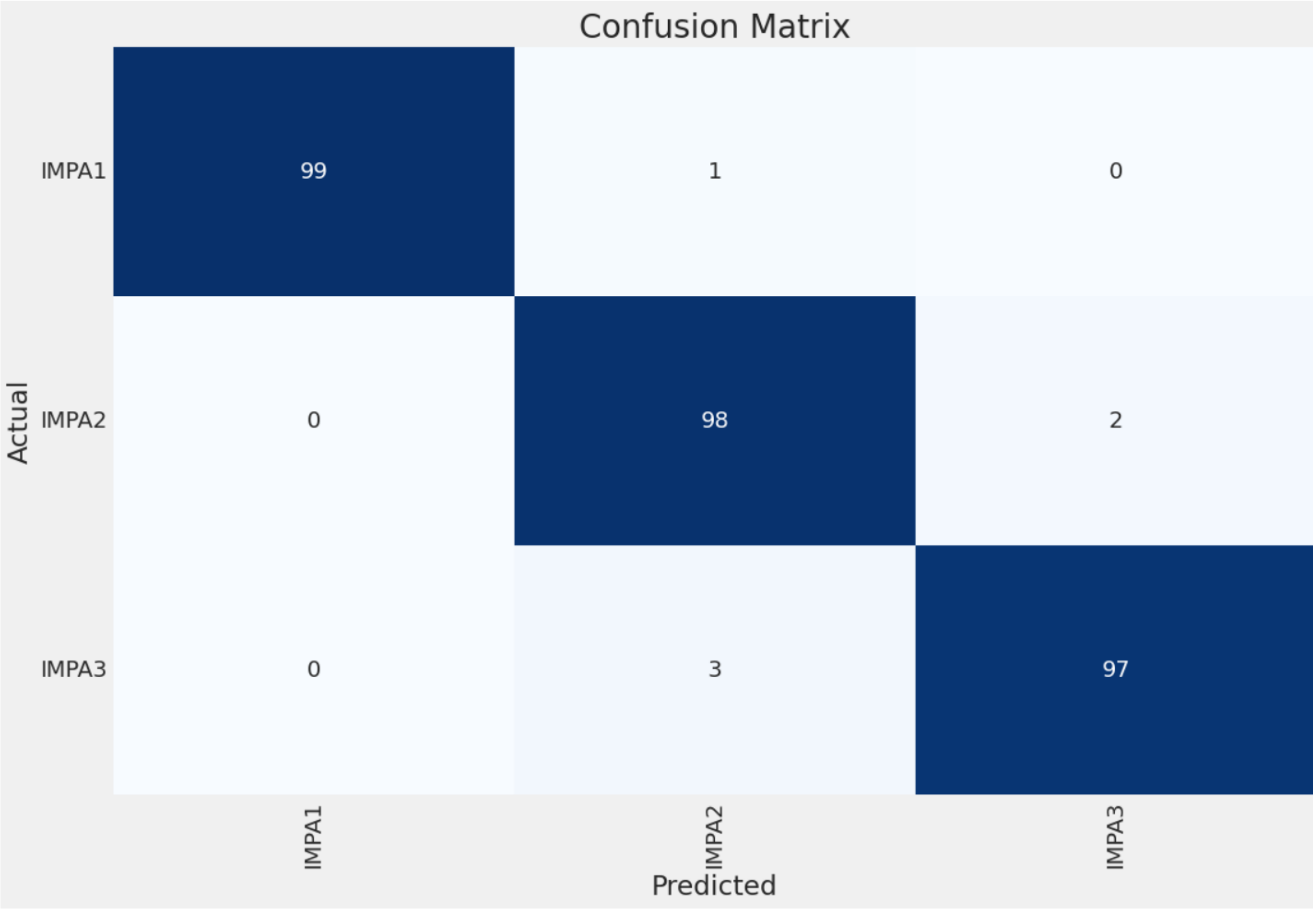

Table S7. Classification Report for IMPA by EFFICIENTNET B0

	precision	recall	f1-score	support
IMPA1	1.0000	0.9900	0.9950	100
IMPA2	0.9608	0.9800	0.9703	100
IMPA3	0.9798	0.9700	0.9749	100
accuracy			0.9800	300
macro avg	0.9802	0.9800	0.9800	300

```
weighted avg      0.9802     0.9800     0.9800       300
```

Figure S13. Training and Validation Loss and Training and Accuracy Graphs for XCEPTION

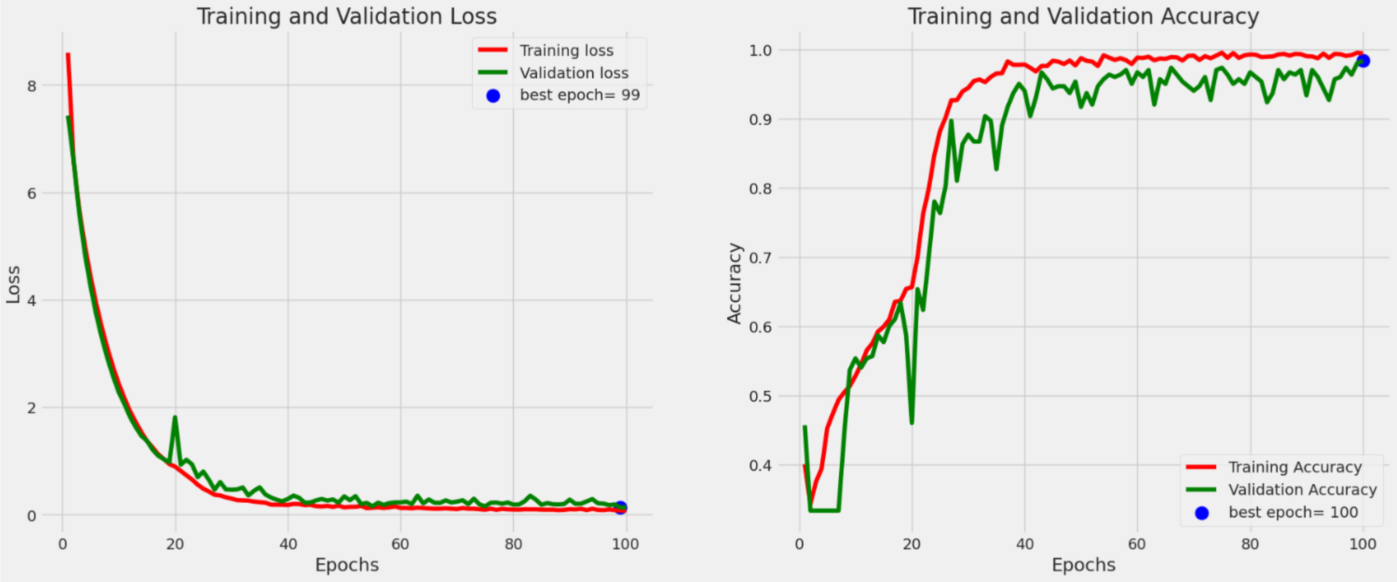

Figure S14. Confusion Matrix for Actual and Predicted IMPA values by XCEPTION

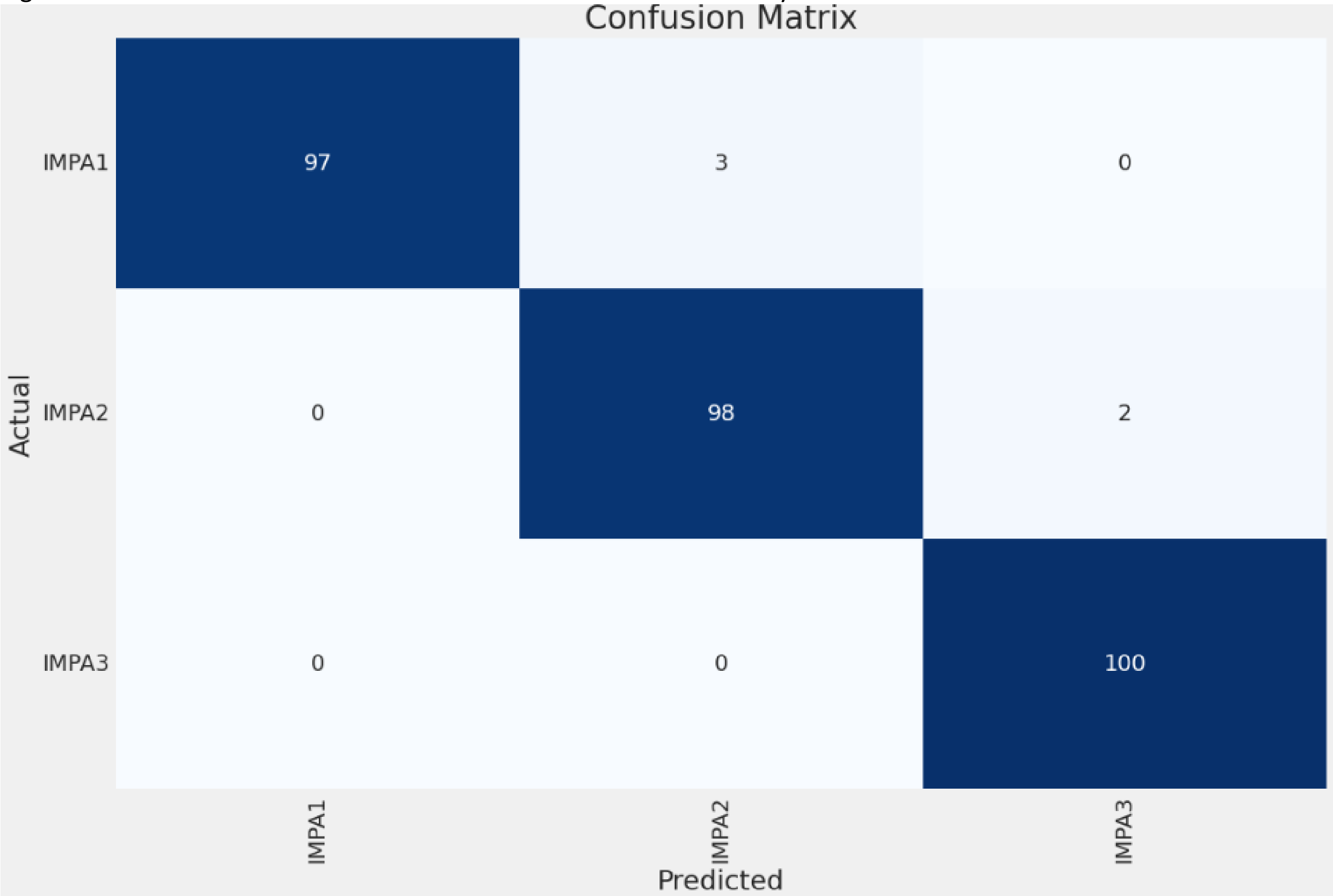

Table S8. Classification Report for IMPA by XCEPTION

	precision	recall	f1-score	support
IMPA1	1.0000	0.9700	0.9848	100
IMPA2	0.9703	0.9800	0.9751	100
IMPA3	0.9804	1.0000	0.9901	100
accuracy			0.9833	300
macro avg	0.9836	0.9833	0.9833	300
weighted avg	0.9836	0.9833	0.9833	300

Figure S15. Training and Validation Loss and Training and Accuracy Graphs for VGG16

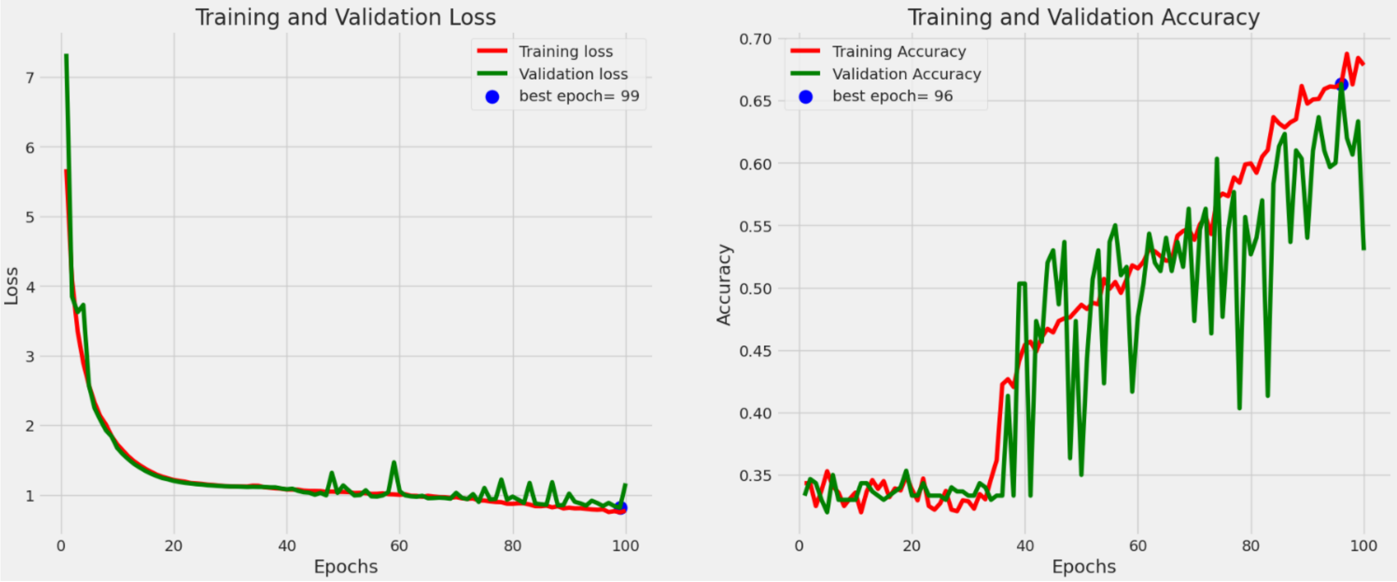

Figure S16. Confusion Matrix for Actual and Predicted IMPA values by VGG16

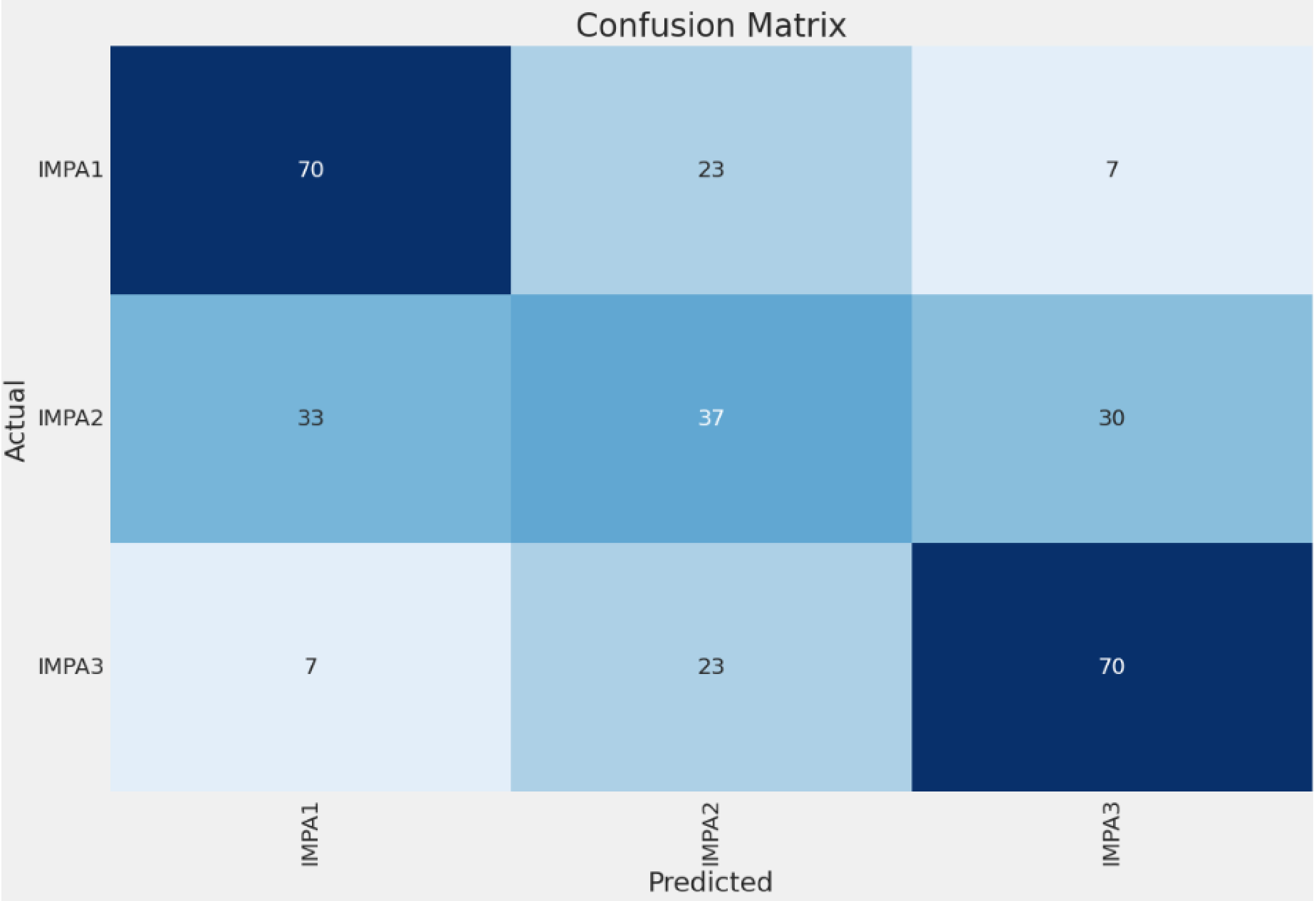

Table S9. Classification Report for IMPA by VGG16

	precision	recall	f1-score	support
IMPA1	0.6364	0.7000	0.6667	100
IMPA2	0.4458	0.3700	0.4044	100
IMPA3	0.6542	0.7000	0.6763	100
accuracy			0.5900	300
macro avg	0.5788	0.5900	0.5825	300
weighted avg	0.5788	0.5900	0.5825	300

Figure S17. Training and Validation Loss and Training and Accuracy Graphs for VGG19

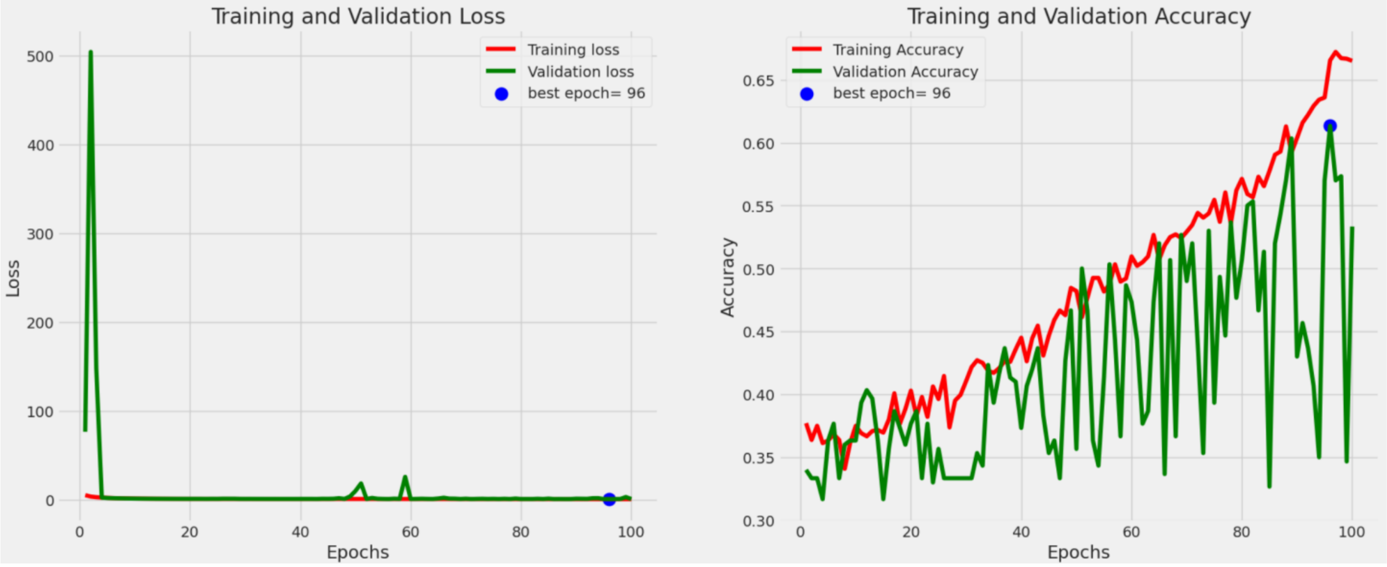

Figure S18. Confusion Matrix for Actual and Predicted IMPA values by VGG19

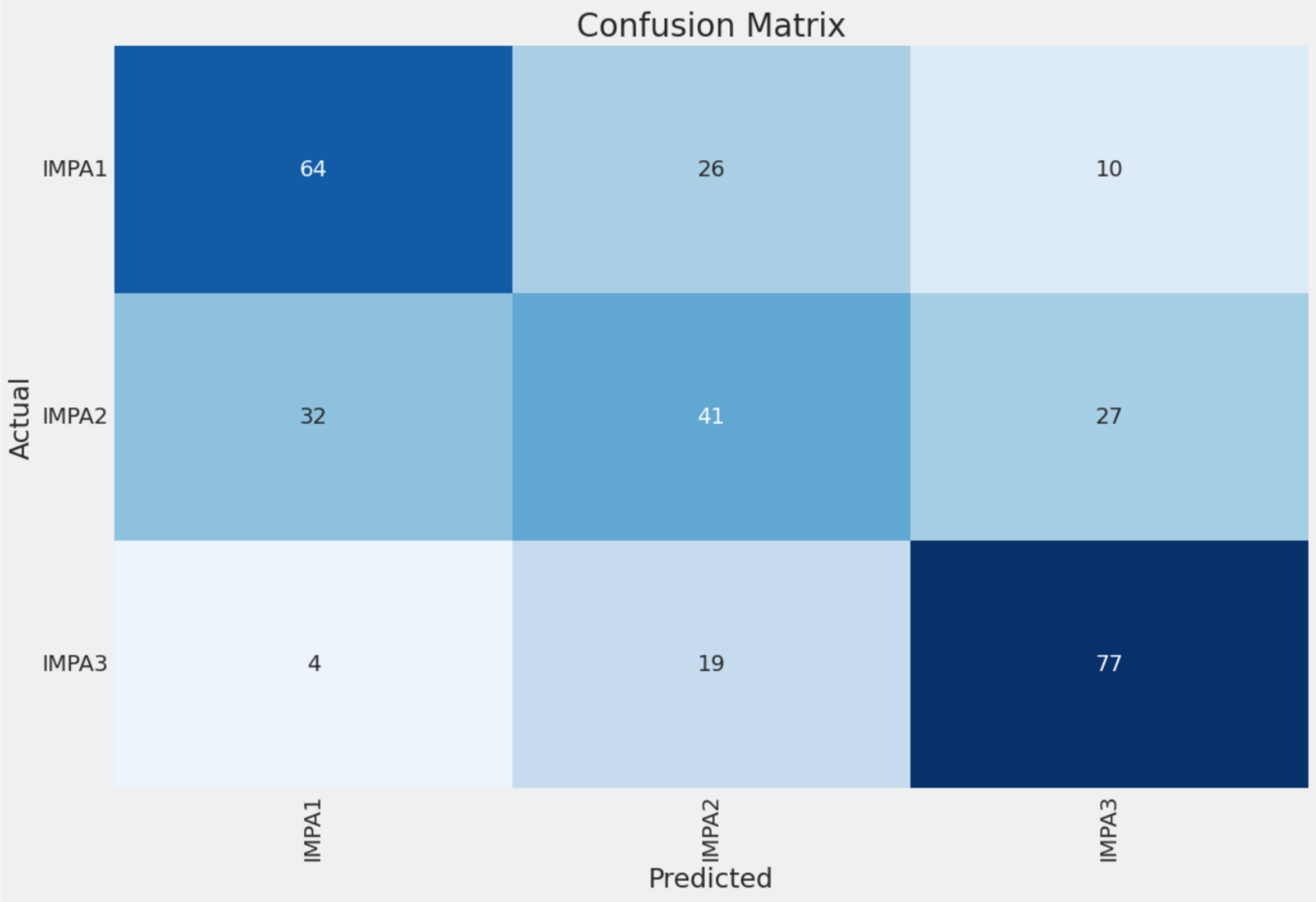

Table S10. Classification Report for IMPA by VGG19

	precision	recall	f1-score	support
IMPA1	0.6400	0.6400	0.6400	100
IMPA2	0.4767	0.4100	0.4409	100
IMPA3	0.6754	0.7700	0.7196	100
accuracy			0.6067	300
macro avg	0.5974	0.6067	0.6002	300

```
weighted avg     0.5974    0.6067    0.6002       300
```

Figure S19. Training and Validation Loss and Training and Accuracy Graphs for NASNETMOBILE

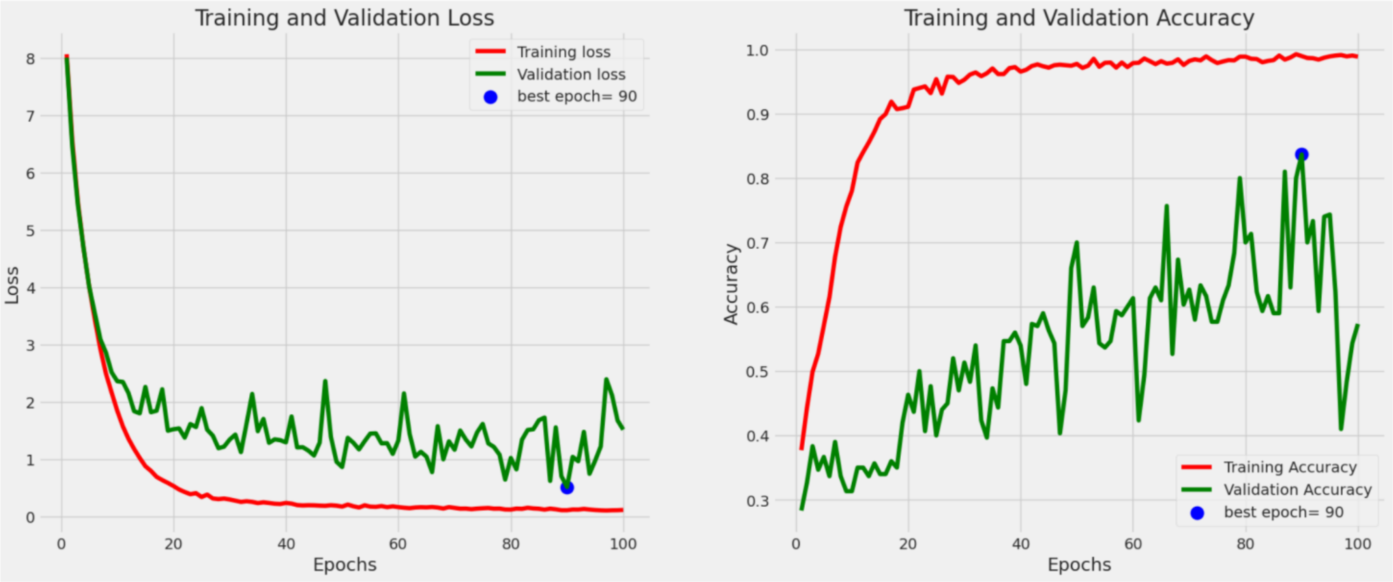

Figure S20. Confusion Matrix for Actual and Predicted IMPA values by NASNETMOBILE

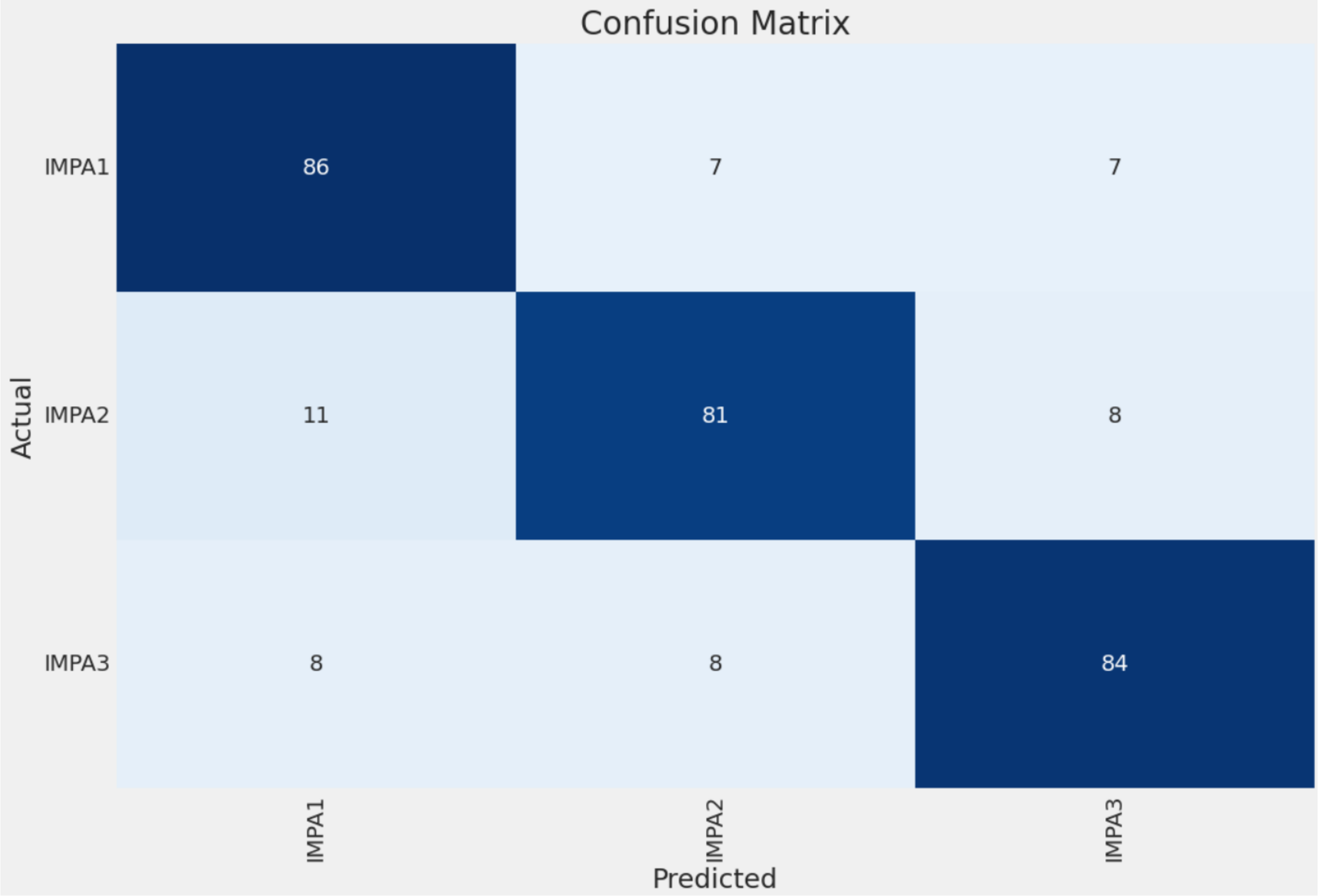

Table S11. Classification Report for IMPA by NASNETMOBILE

----

	precision	recall	f1-score	support
IMPA1	0.8190	0.8600	0.8390	100
IMPA2	0.8438	0.8100	0.8265	100
IMPA3	0.8485	0.8400	0.8442	100
accuracy			0.8367	300
macro avg	0.8371	0.8367	0.8366	300
weighted avg	0.8371	0.8367	0.8366	300

Figure S21. Training and Validation Loss and Training and Accuracy Graphs for RESNET101

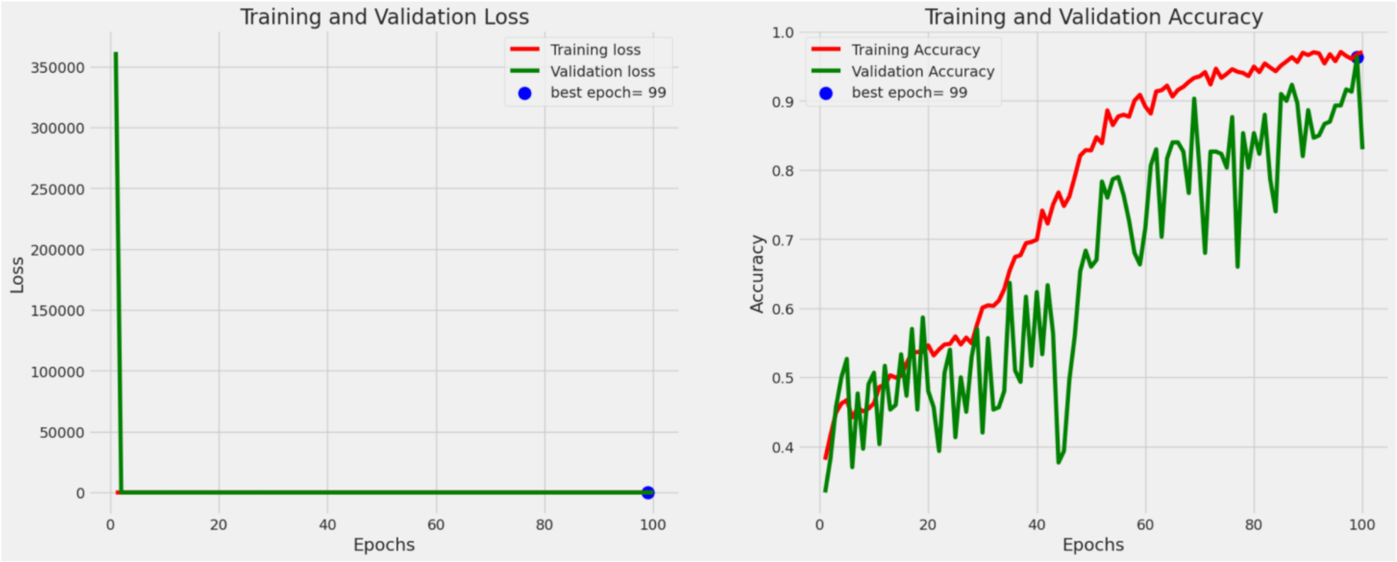

Figure S22. Confusion Matrix for Actual and Predicted IMPA values by RESNET101

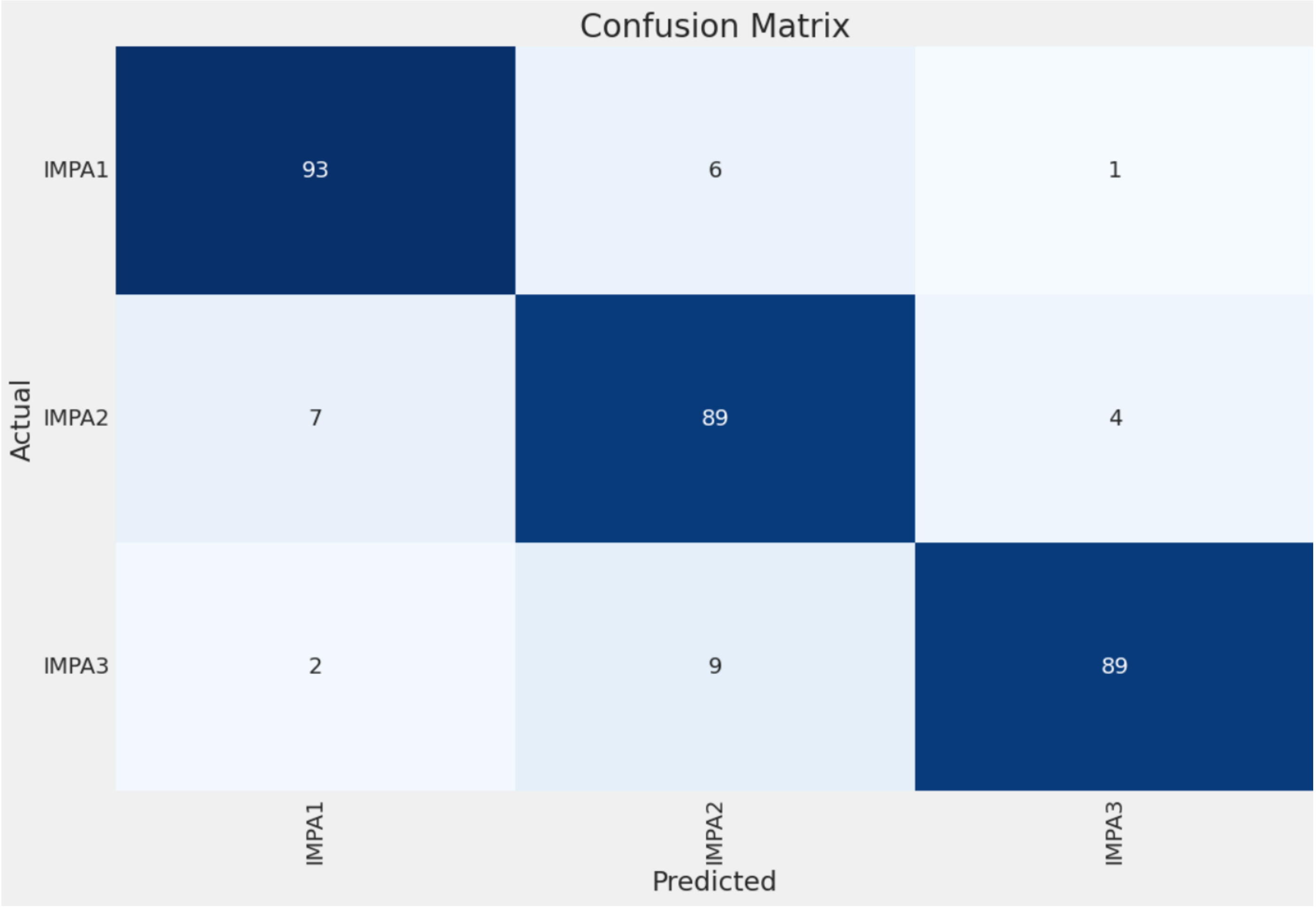

Table S12. Classification Report for IMPA by RESNET101

----

	precision	recall	f1-score	support
IMPA1	0.9118	0.9300	0.9208	100
IMPA2	0.8558	0.8900	0.8725	100
IMPA3	0.9468	0.8900	0.9175	100
accuracy			0.9033	300
macro avg	0.9048	0.9033	0.9036	300

weighted avg	0.9048	0.9033	0.9036	300

Figure S23. Training and Validation Loss and Training and Accuracy Graphs for RESNET152

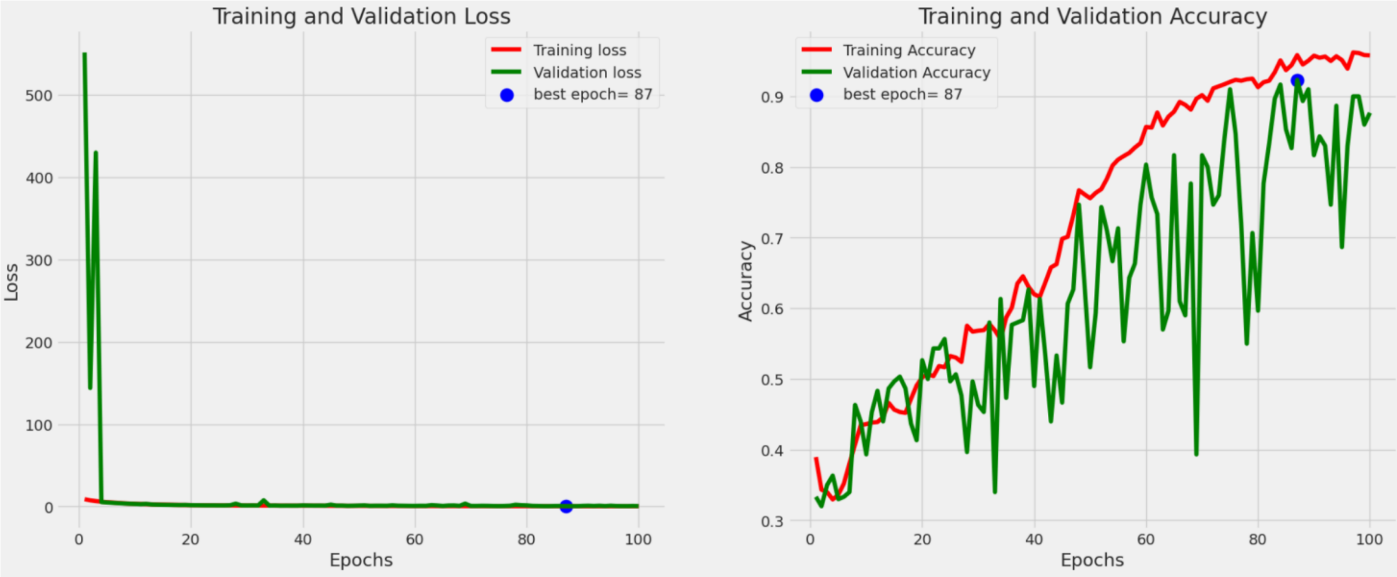

Figure S24. Confusion Matrix for Actual and Predicted IMPA values by RESNET152

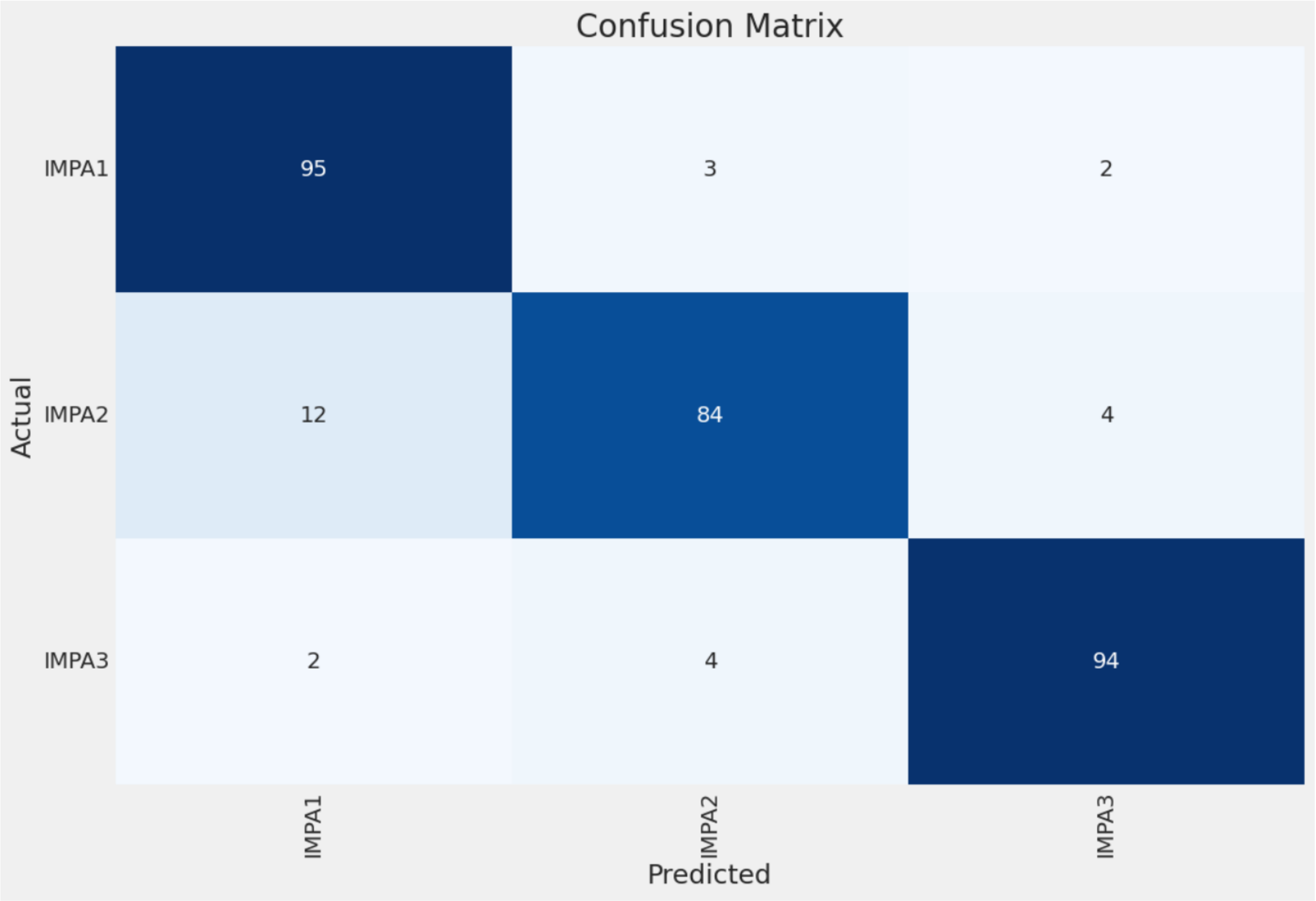

Table S13. Classification Report for IMPA by RESNET152

	precision	recall	f1-score	support
IMPA1	0.8716	0.9500	0.9091	100
IMPA2	0.9231	0.8400	0.8796	100
IMPA3	0.9400	0.9400	0.9400	100
accuracy			0.9100	300
macro avg	0.9115	0.9100	0.9096	300
weighted avg	0.9115	0.9100	0.9096	300

Figure S25. Training and Validation Loss and Training and Accuracy Graphs for RESNET50

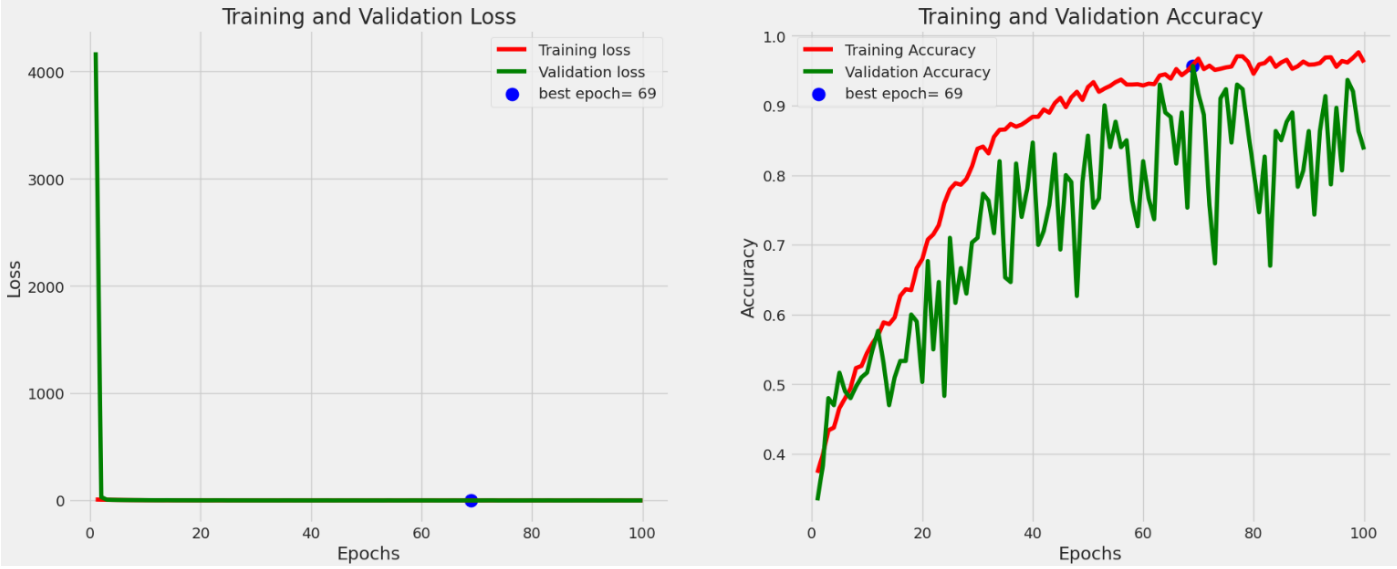

Figure S26. Confusion Matrix for Actual and Predicted IMPA values by RESNET50

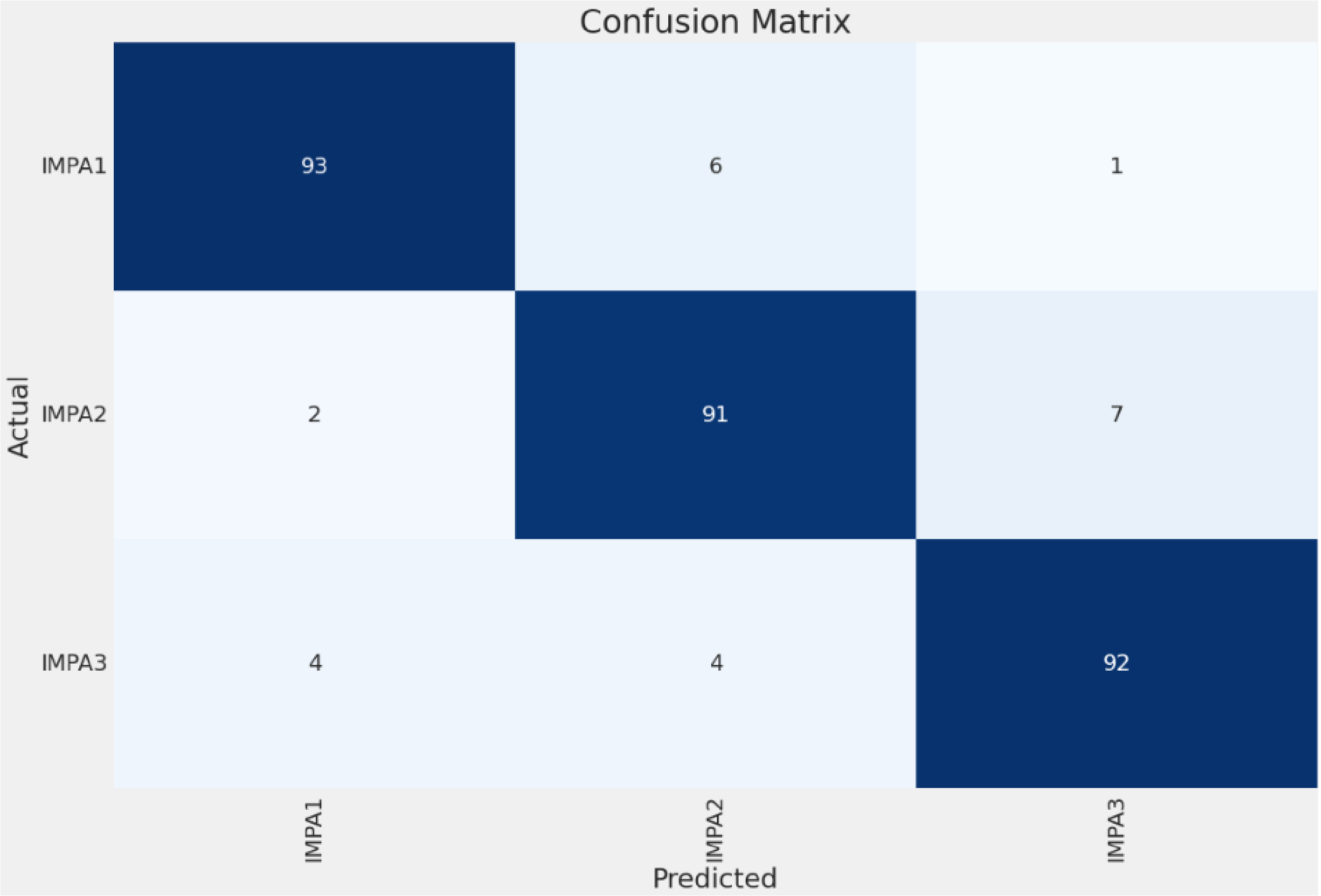

Table S14. Classification Report for IMPA by RESNET50

	precision	recall	f1-score	support
IMPA1	0.9394	0.9300	0.9347	100
IMPA2	0.9010	0.9100	0.9055	100
IMPA3	0.9200	0.9200	0.9200	100
accuracy			0.9200	300
macro avg	0.9201	0.9200	0.9200	300
weighted avg	0.9201	0.9200	0.9200	300

Figure S27. Training and Validation Loss and Training and Accuracy Graphs for EFFICIENTNET V2

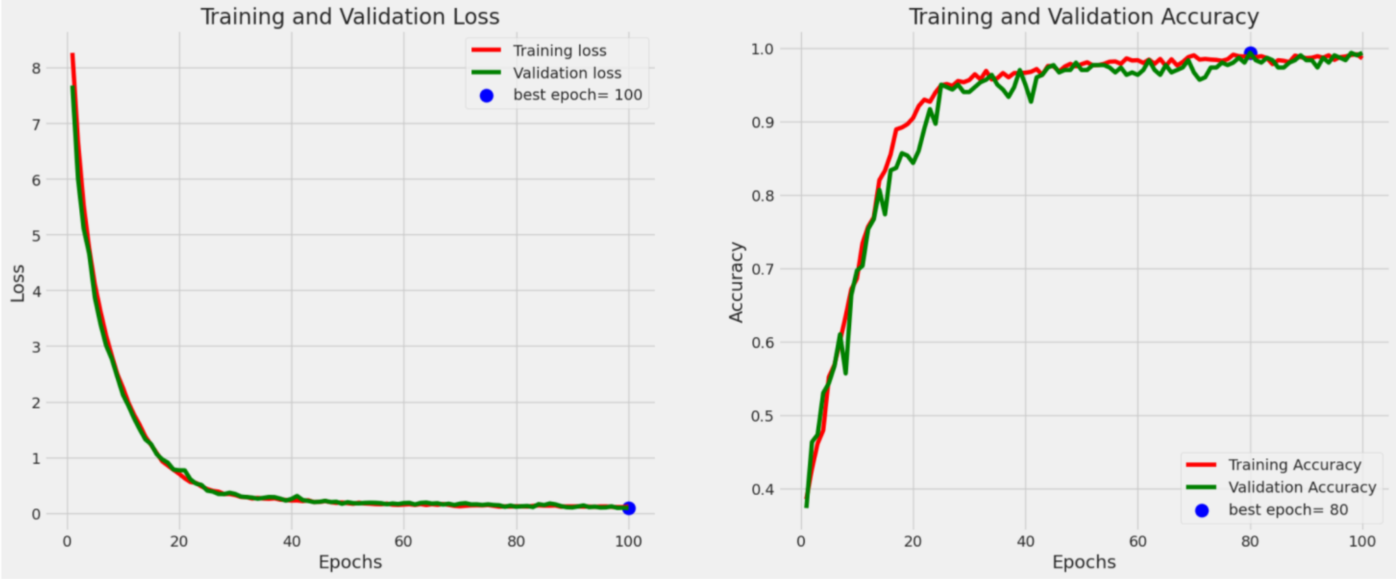

Figure S28. Confusion Matrix for Actual and Predicted IMPA values by EFFICIENTNET V2

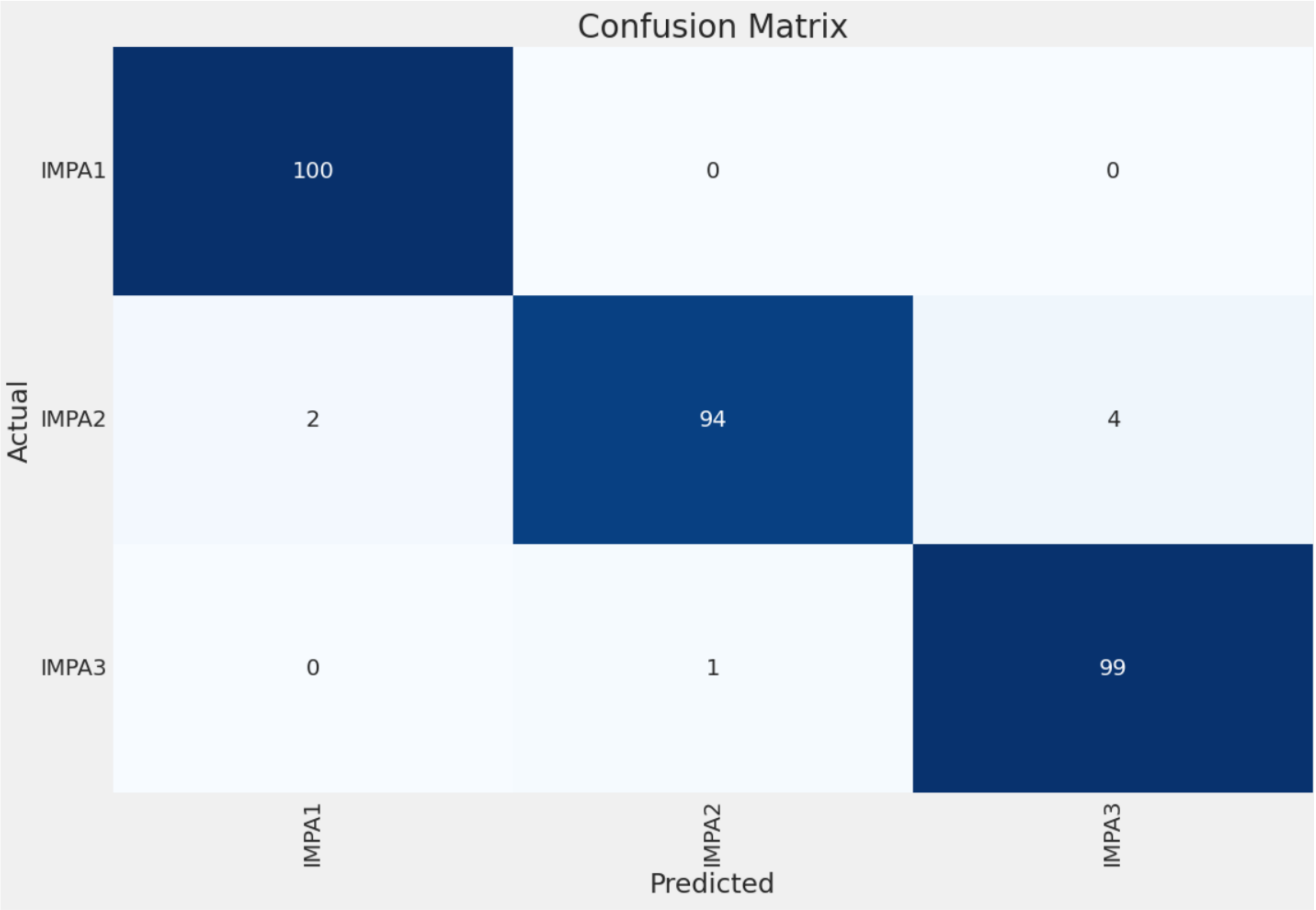

Table S15. Classification Report for IMPA by EFFICIENTNET V2

	precision	recall	f1-score	support
IMPA1	0.9804	1.0000	0.9901	100
IMPA2	0.9895	0.9400	0.9641	100
IMPA3	0.9612	0.9900	0.9754	100
accuracy			0.9767	300
macro avg	0.9770	0.9767	0.9765	300

```
weighted avg      0.9770     0.9767     0.9765        300
```

Table S16. Summary table for accuracy values of U1-L1 Classification

INTERINCISAL	
MOBILENET V2	93.67
INCEPTION V3	57.00
DENSENET 121	95.00
DENSENET 169	96.33
DENSENET 201	95.67
EFFICIENTNET B0	97.67
XCEPTION	97.00
VGG16	53.00
VGG19	51.67
NASNETMOBILE	87.00
RESNET101	92.67 (GRAPH)
RESNET152	71.00 (GRAPH)
RESNET50	89.67 (GRAPH)
EFFICIENTNET V2	99.00

Figure S29. Training and Validation Loss and Training and Accuracy Graphs for MOBILENET V2

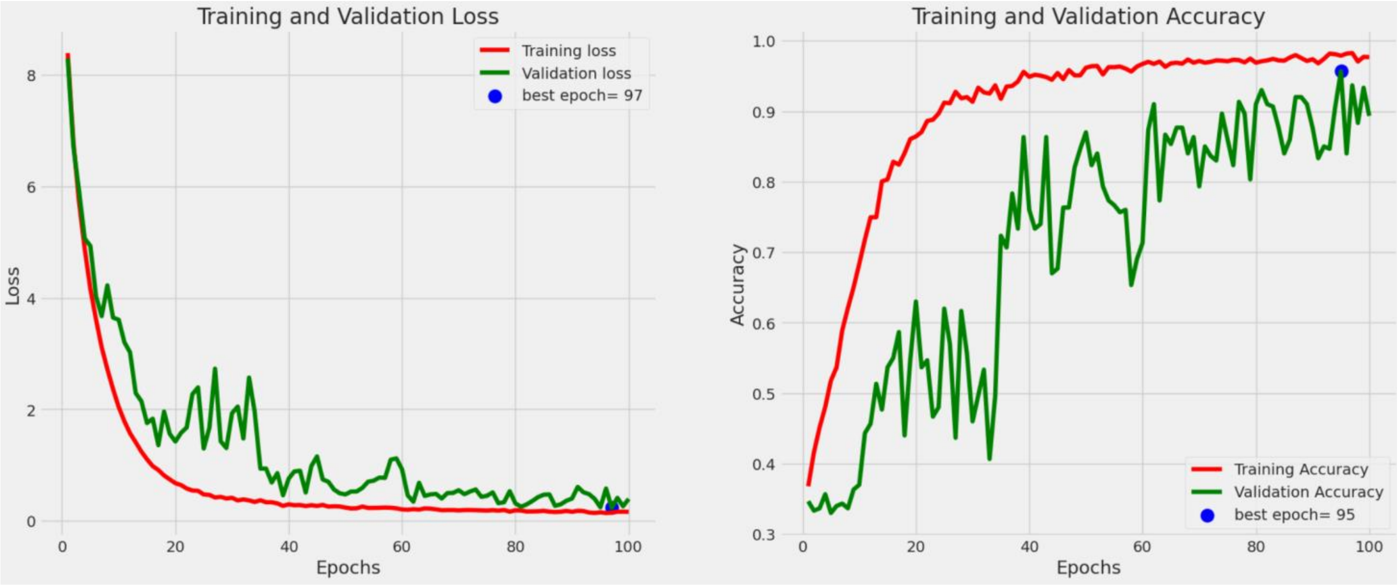

Figure S30. Confusion Matrix for Actual and Predicted U1-L1 values by MOBILENET V2

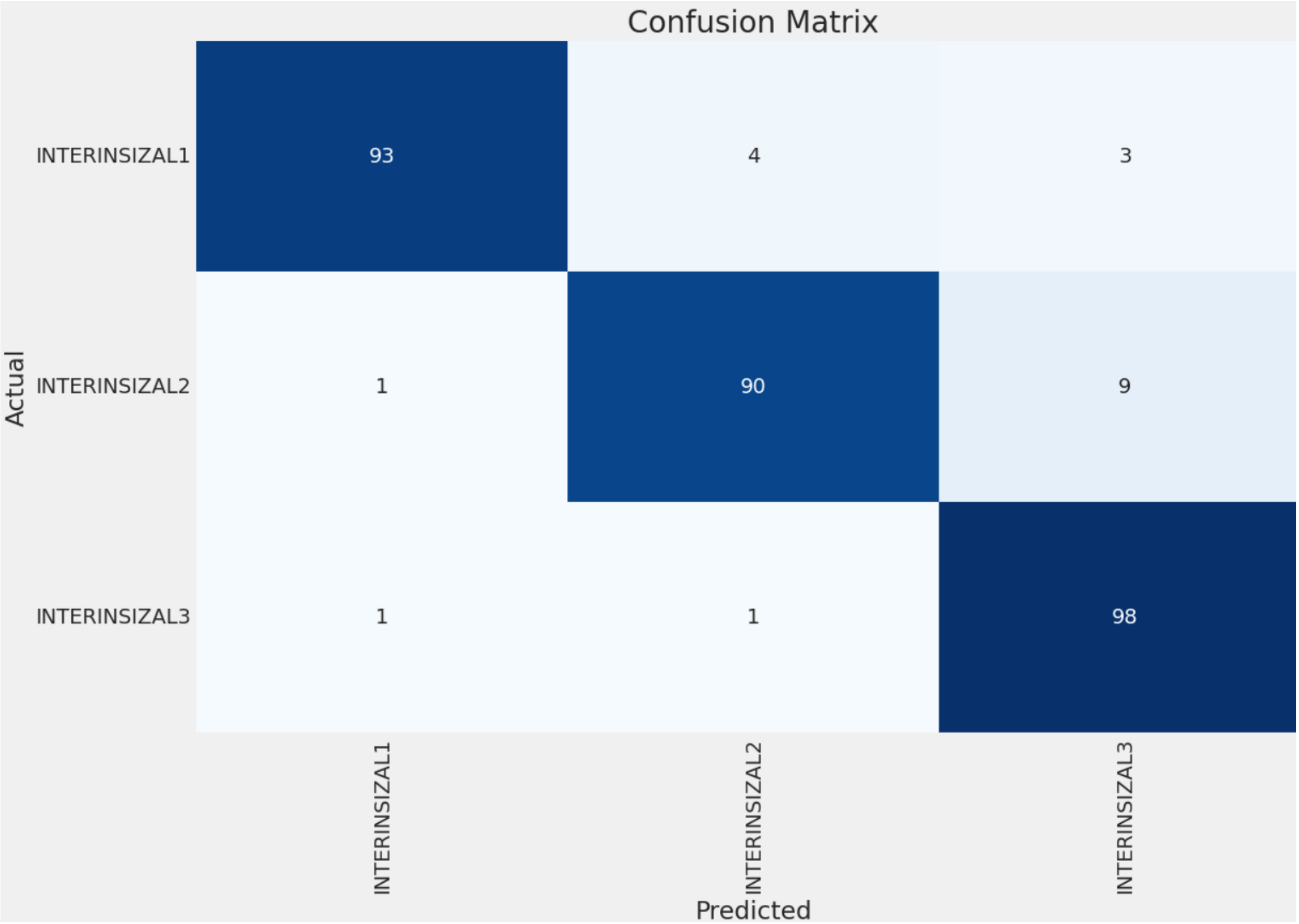

Table S17. Classification Report for U1-L1 by MOBILENET V2

	precision	recall	f1-score	support
INTERINSIZAL1	0.9789	0.9300	0.9538	100
INTERINSIZAL2	0.9474	0.9000	0.9231	100
INTERINSIZAL3	0.8909	0.9800	0.9333	100
accuracy			0.9367	300
macro avg	0.9391	0.9367	0.9368	300
weighted avg	0.9391	0.9367	0.9368	300

Figure S31. Training and Validation Loss and Training and Accuracy Graphs for INCEPTION V3

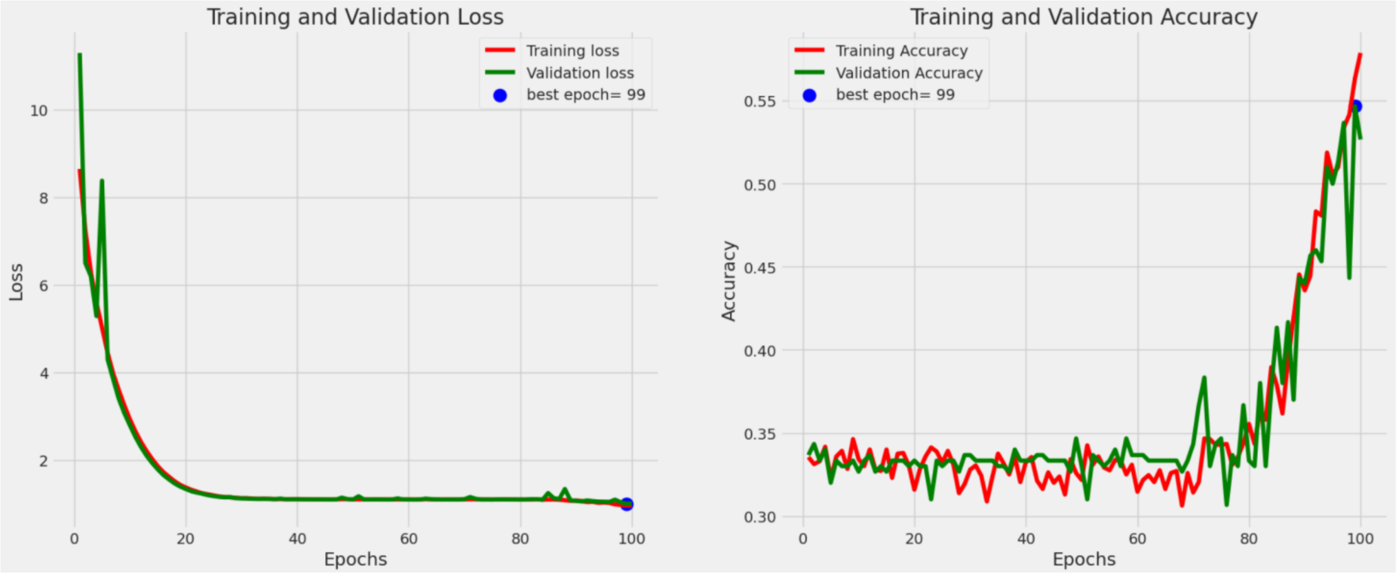

Figure S32. Confusion Matrix for Actual and Predicted U1-L1 values by INCEPTION V3

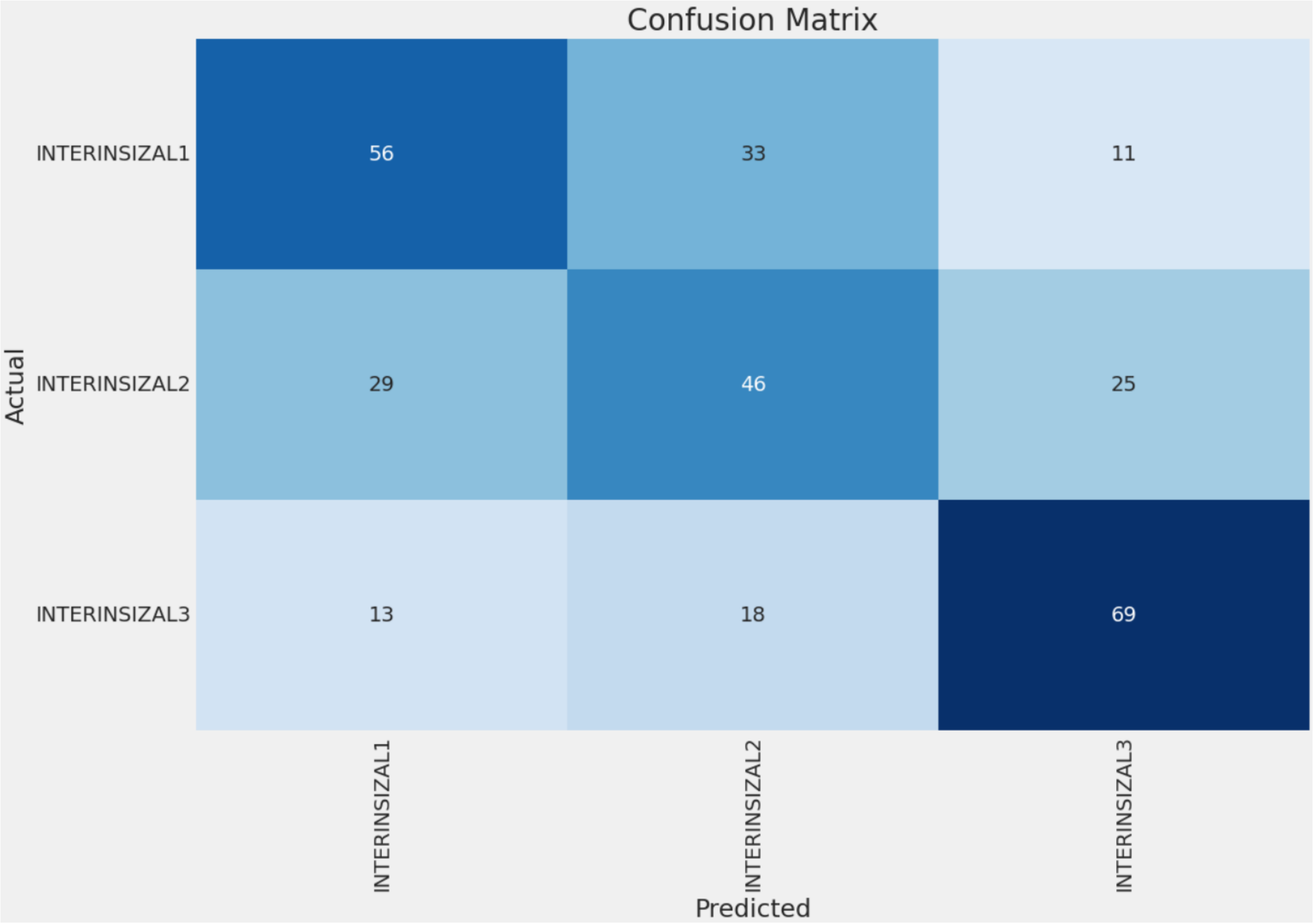

Table S18. Classification Report for U1-L1 by INCEPTION V3

	precision	recall	f1-score	support
INTERINSIZAL1	0.5714	0.5600	0.5657	100
INTERINSIZAL2	0.4742	0.4600	0.4670	100
INTERINSIZAL3	0.6571	0.6900	0.6732	100

accuracy			0.5700	300
macro avg	0.5676	0.5700	0.5686	300
weighted avg	0.5676	0.5700	0.5686	300

Figure S33. Training and Validation Loss and Training and Accuracy Graphs for DENSENET 121

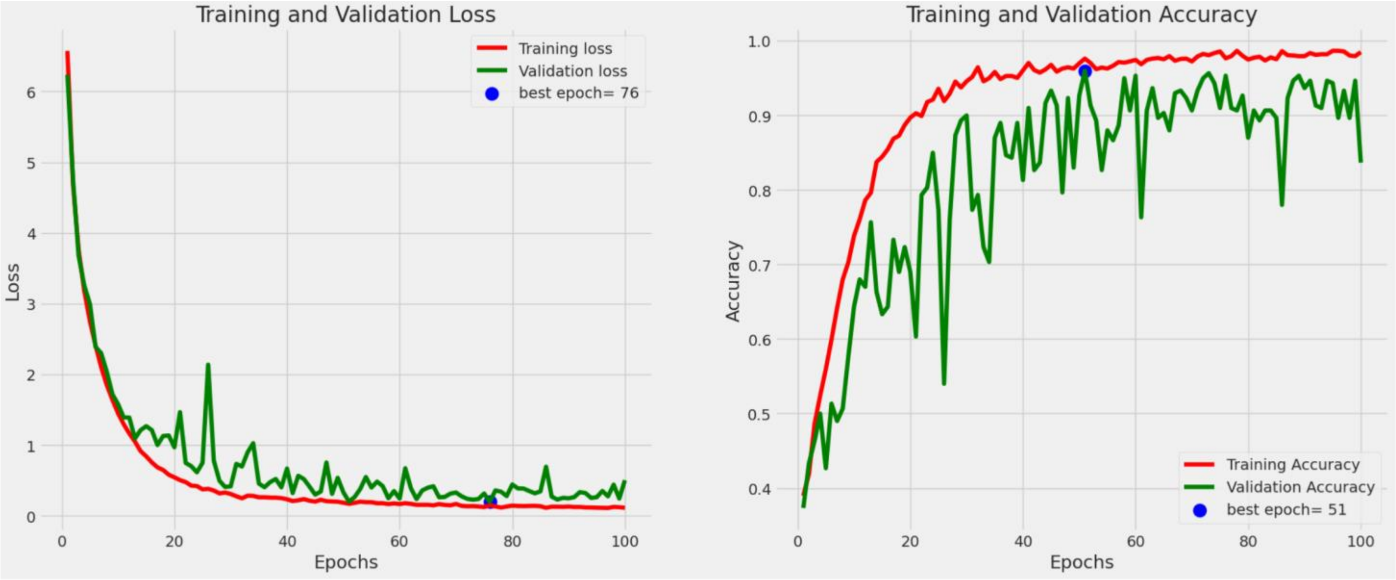

Figure S34. Confusion Matrix for Actual and Predicted U1-L1 values by DENSENET 121

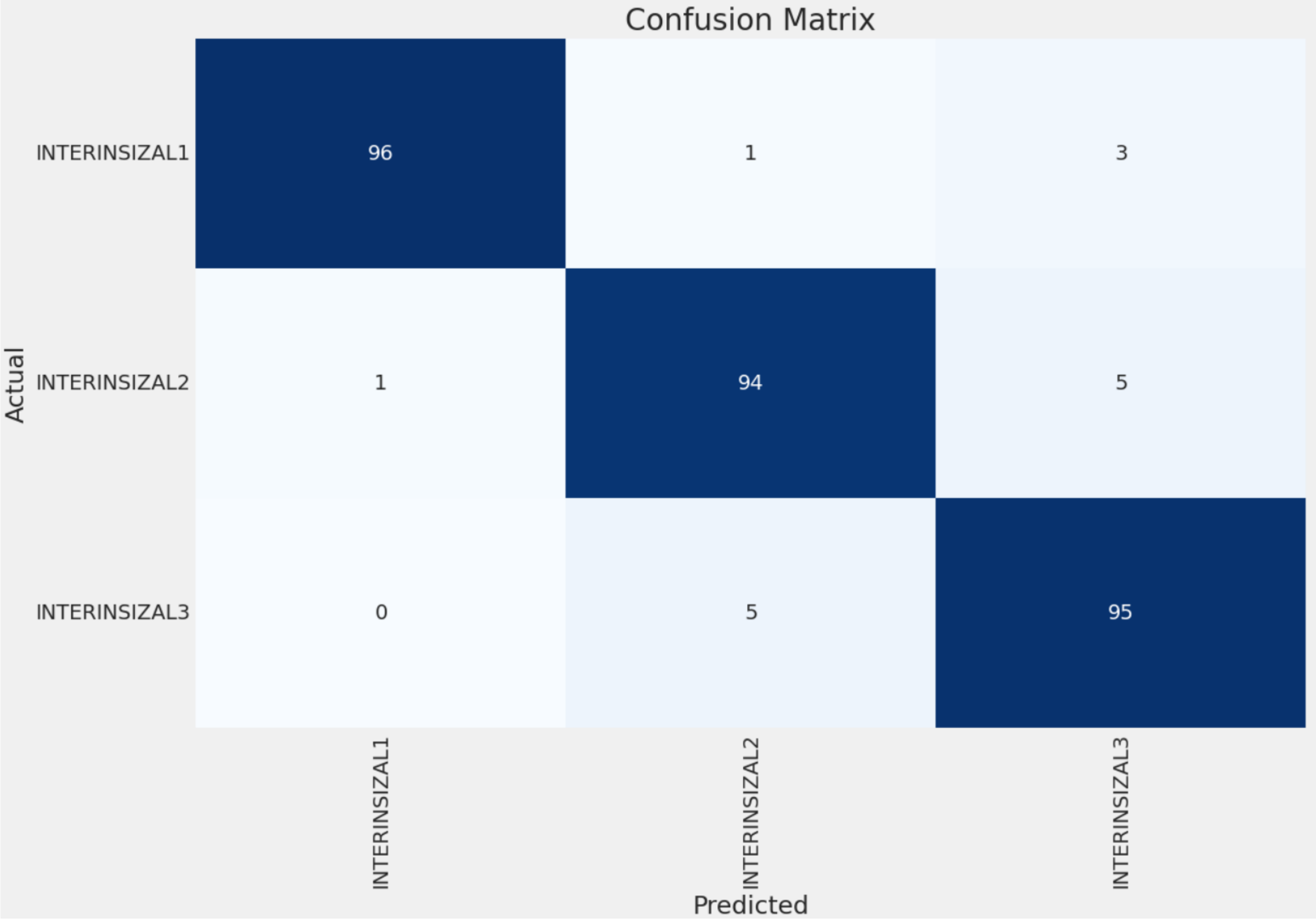

Table S19. Classification Report for U1-L1 by DENSENET 121

	precision	recall	f1-score	support
INTERINSIZAL1	0.9897	0.9600	0.9746	100
INTERINSIZAL2	0.9400	0.9400	0.9400	100
INTERINSIZAL3	0.9223	0.9500	0.9360	100
accuracy			0.9500	300
macro avg	0.9507	0.9500	0.9502	300
weighted avg	0.9507	0.9500	0.9502	300

Figure S35. Training and Validation Loss and Training and Accuracy Graphs for DENSENET 169

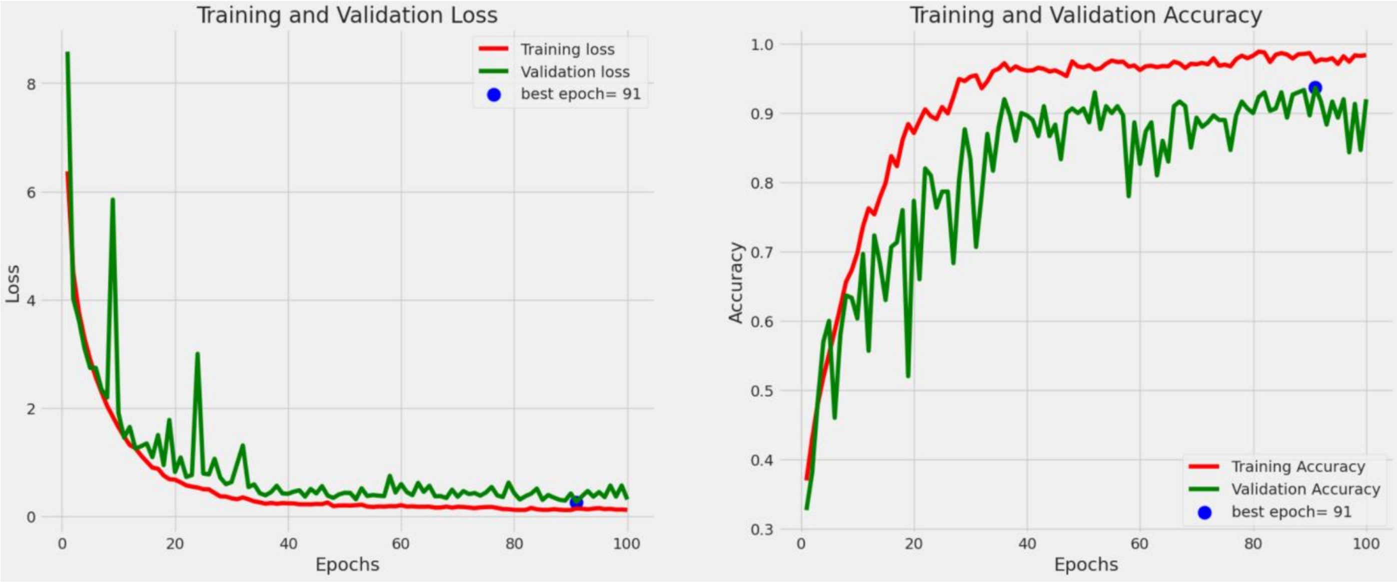

Figure S36. Confusion Matrix for Actual and Predicted U1-L1 values by DENSENET 169

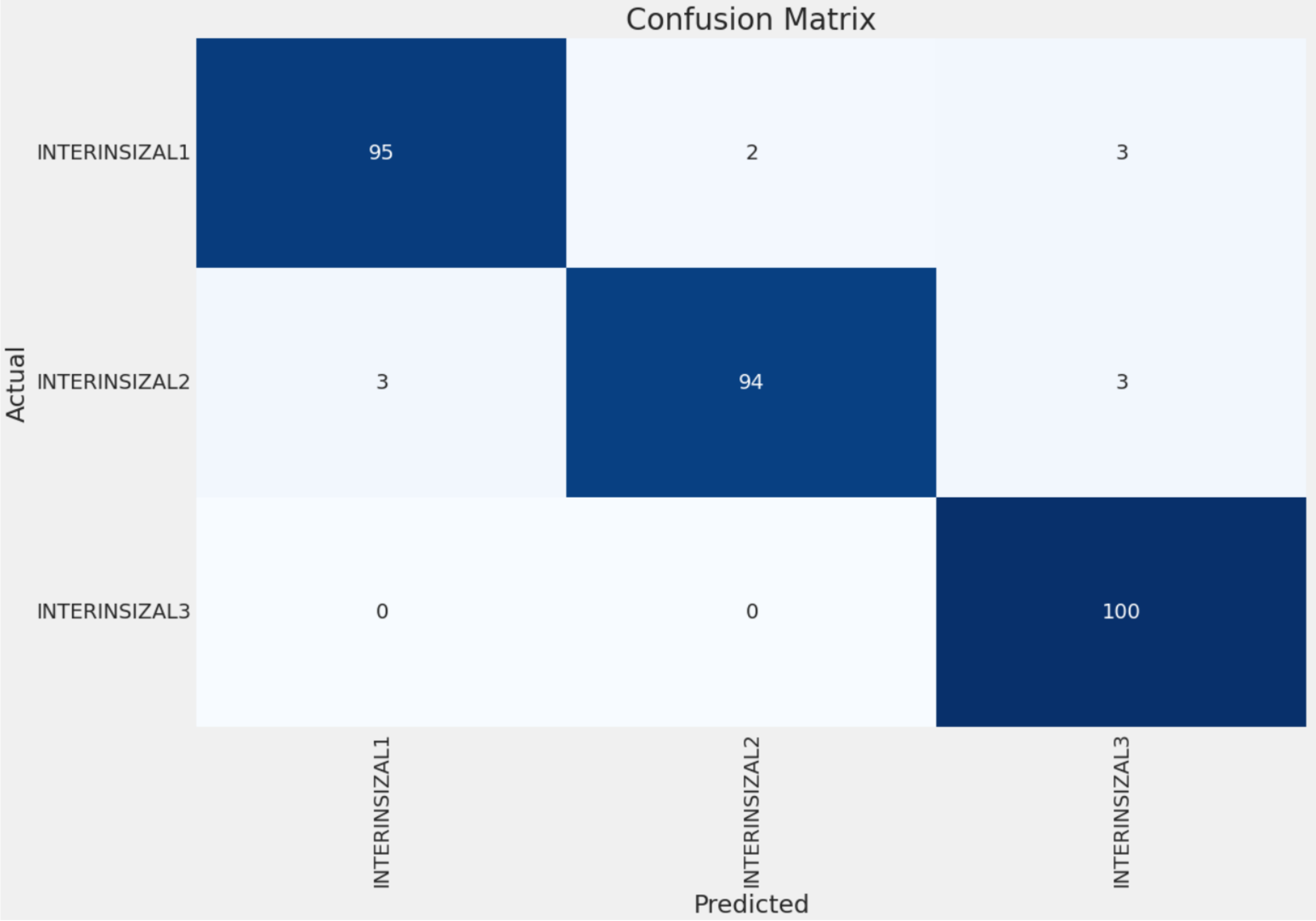

Table S20. Classification Report for U1-L1 by DENSENET 169

	precision	recall	f1-score	support
INTERINSIZAL1	0.9694	0.9500	0.9596	100
INTERINSIZAL2	0.9792	0.9400	0.9592	100
INTERINSIZAL3	0.9434	1.0000	0.9709	100

accuracy			0.9633	300
macro avg	0.9640	0.9633	0.9632	300
weighted avg	0.9640	0.9633	0.9632	300

Figure S37. Training and Validation Loss and Training and Accuracy Graphs for DENSENET 201

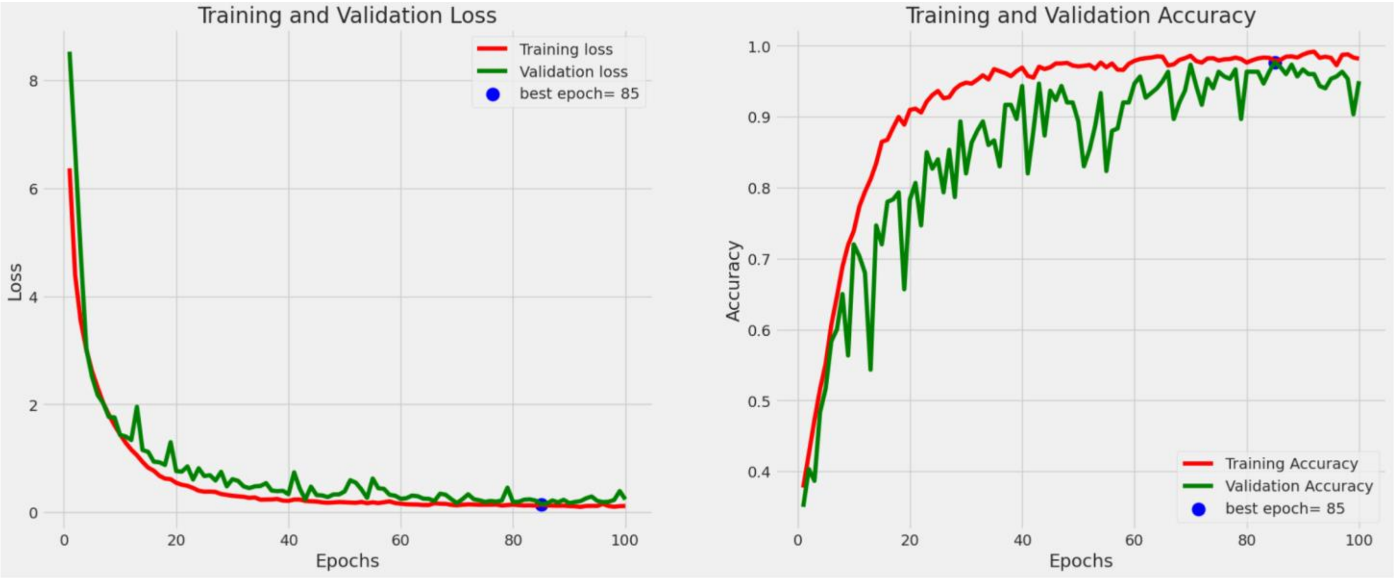

Figure S38. Confusion Matrix for Actual and Predicted U1-L1 values by DENSENET 201

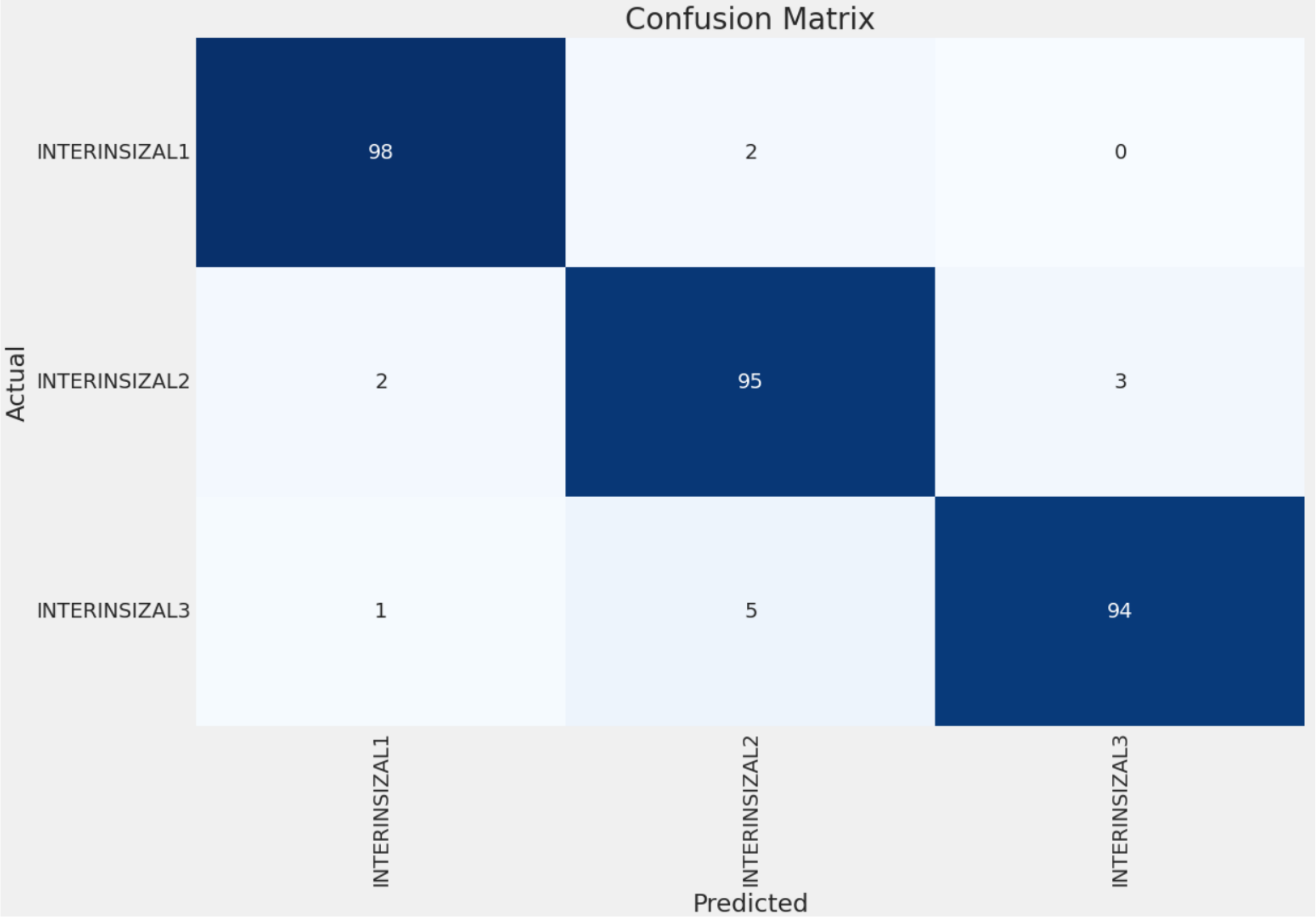

Table S21. Classification Report for U1-L1 by DENSENET 201

	precision	recall	f1-score	support
INTERINSIZAL1	0.9703	0.9800	0.9751	100
INTERINSIZAL2	0.9314	0.9500	0.9406	100
INTERINSIZAL3	0.9691	0.9400	0.9543	100
accuracy			0.9567	300
macro avg	0.9569	0.9567	0.9567	300
weighted avg	0.9569	0.9567	0.9567	300

Figure S39. Training and Validation Loss and Training and Accuracy Graphs for EFFICIENTNET B0

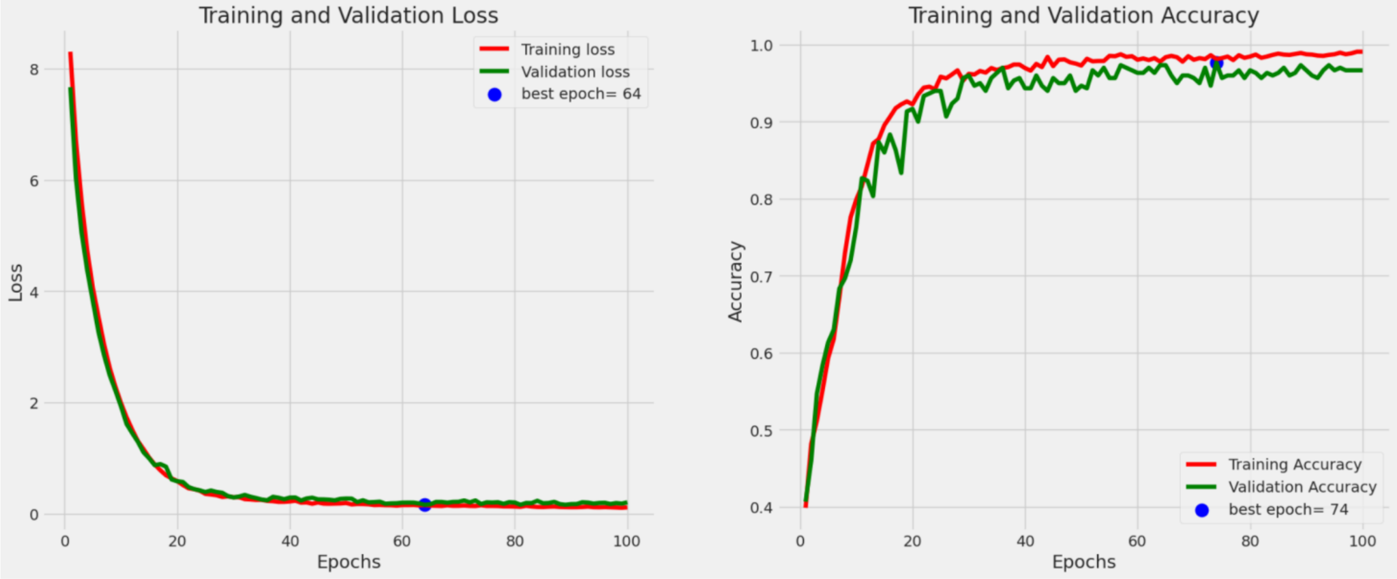

Figure S40. Confusion Matrix for Actual and Predicted U1-L1 values by EFFICIENTNET B0

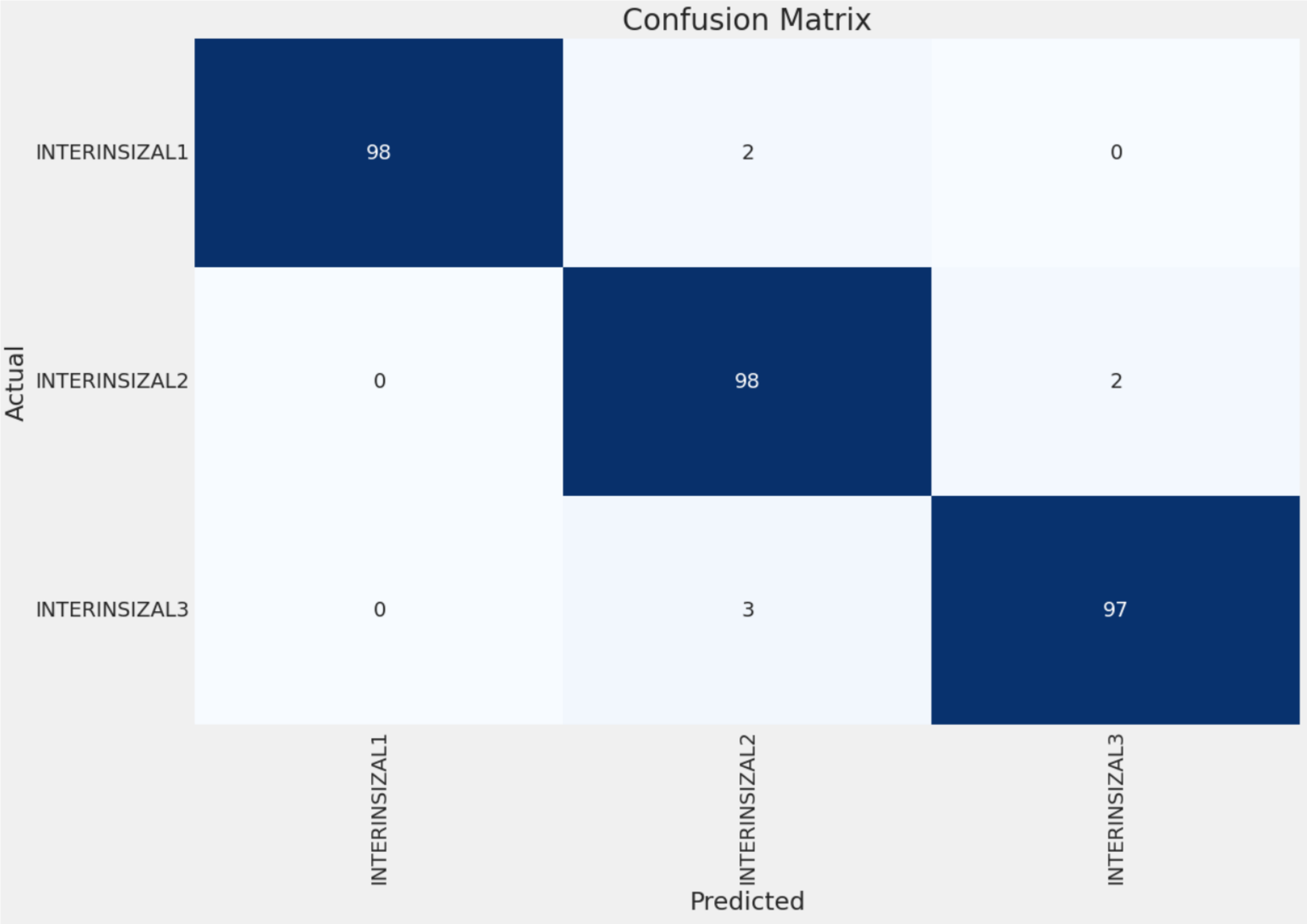

Table S22. Classification Report for U1-L1 by EFFICIENTNET B0

	precision	recall	f1-score	support
INTERINSIZAL1	1.0000	0.9800	0.9899	100
INTERINSIZAL2	0.9515	0.9800	0.9655	100
INTERINSIZAL3	0.9798	0.9700	0.9749	100
accuracy			0.9767	300

macro avg	0.9771	0.9767	0.9768	300
weighted avg	0.9771	0.9767	0.9768	300

Figure S41. Training and Validation Loss and Training and Accuracy Graphs for XCEPTION

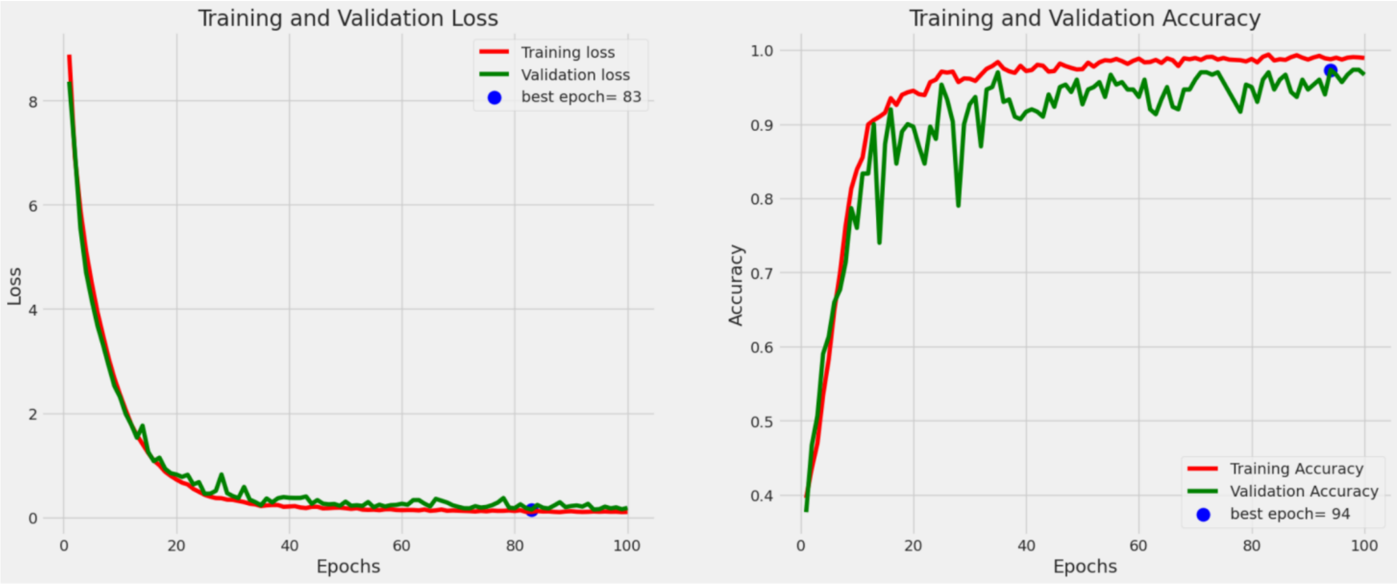

Figure S42. Confusion Matrix for Actual and Predicted U1-L1 values by XCEPTION

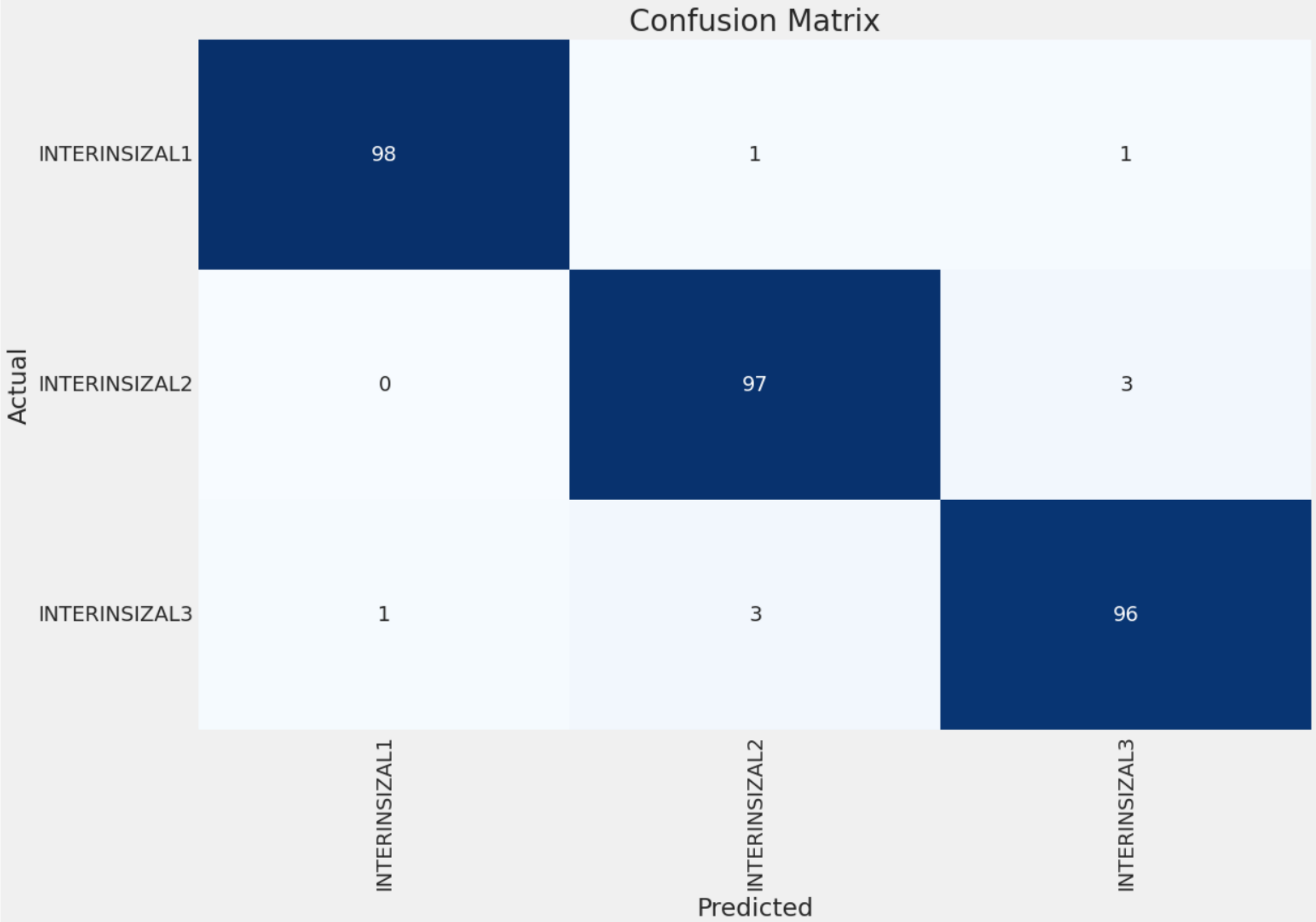

Table S23. Classification Report for U1-L1 by XCEPTION

----

	precision	recall	f1-score	support
INTERINSIZAL1	0.9899	0.9800	0.9849	100
INTERINSIZAL2	0.9604	0.9700	0.9652	100
INTERINSIZAL3	0.9600	0.9600	0.9600	100
accuracy			0.9700	300
macro avg	0.9701	0.9700	0.9700	300
weighted avg	0.9701	0.9700	0.9700	300

Figure S43. Training and Validation Loss and Training and Accuracy Graphs for VGG16

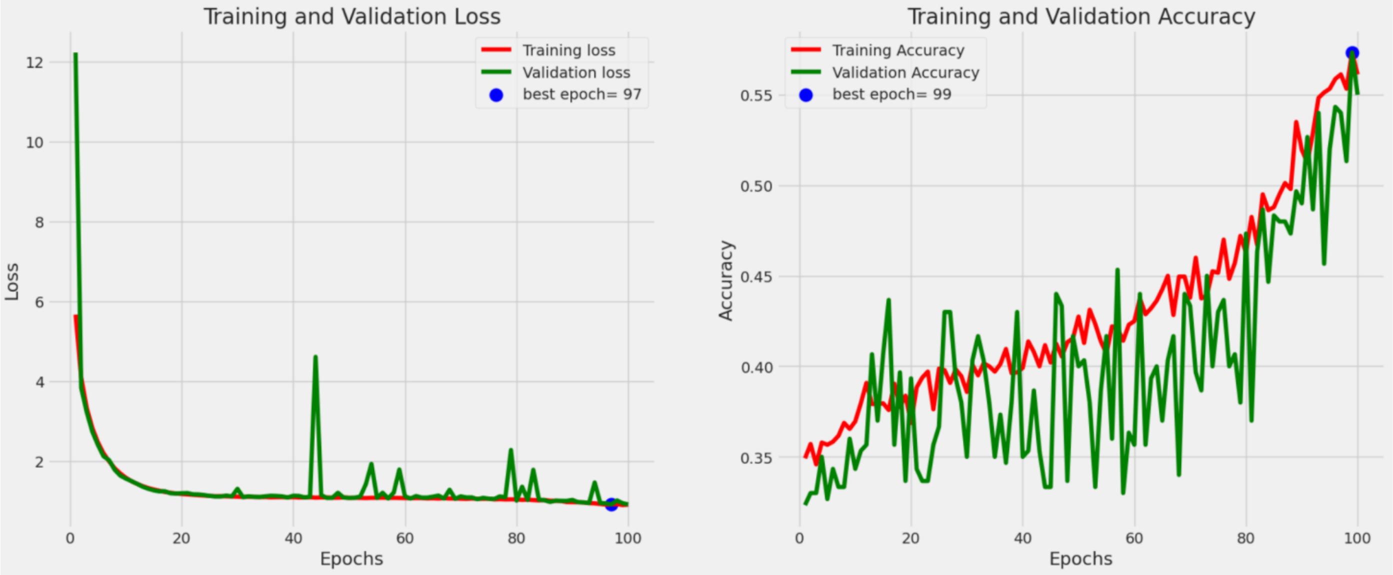

Figure S44. Confusion Matrix for Actual and Predicted U1-L1 values by VGG16

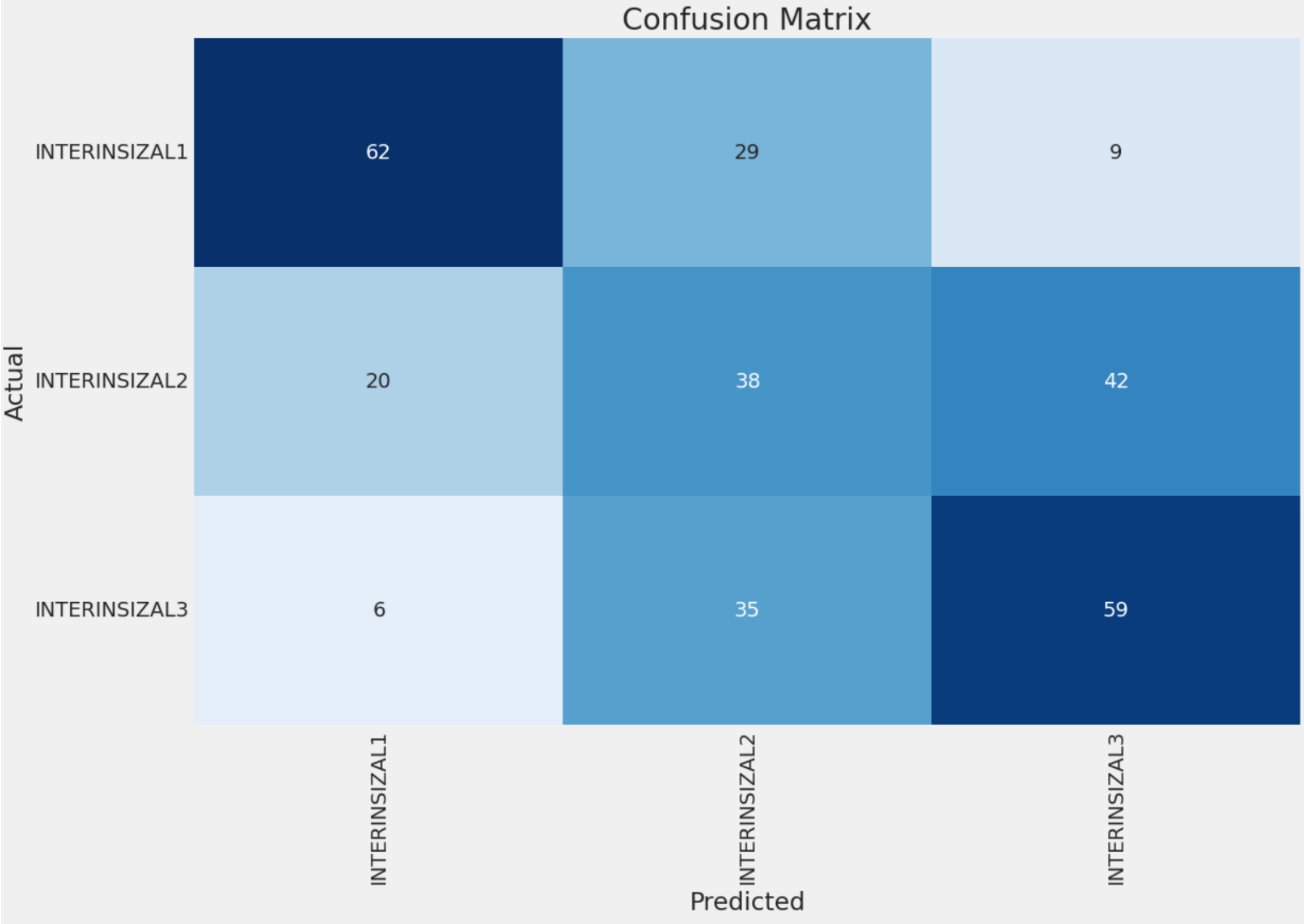

Table S24. Classification Report for U1-L1 by VGG16

	precision	recall	f1-score	support
INTERINSIZAL1	0.7045	0.6200	0.6596	100
INTERINSIZAL2	0.3725	0.3800	0.3762	100
INTERINSIZAL3	0.5364	0.5900	0.5619	100
accuracy			0.5300	300
macro avg	0.5378	0.5300	0.5326	300

weighted avg	0.5378	0.5300	0.5326	300

Figure S45. Training and Validation Loss and Training and Accuracy Graphs for VGG19

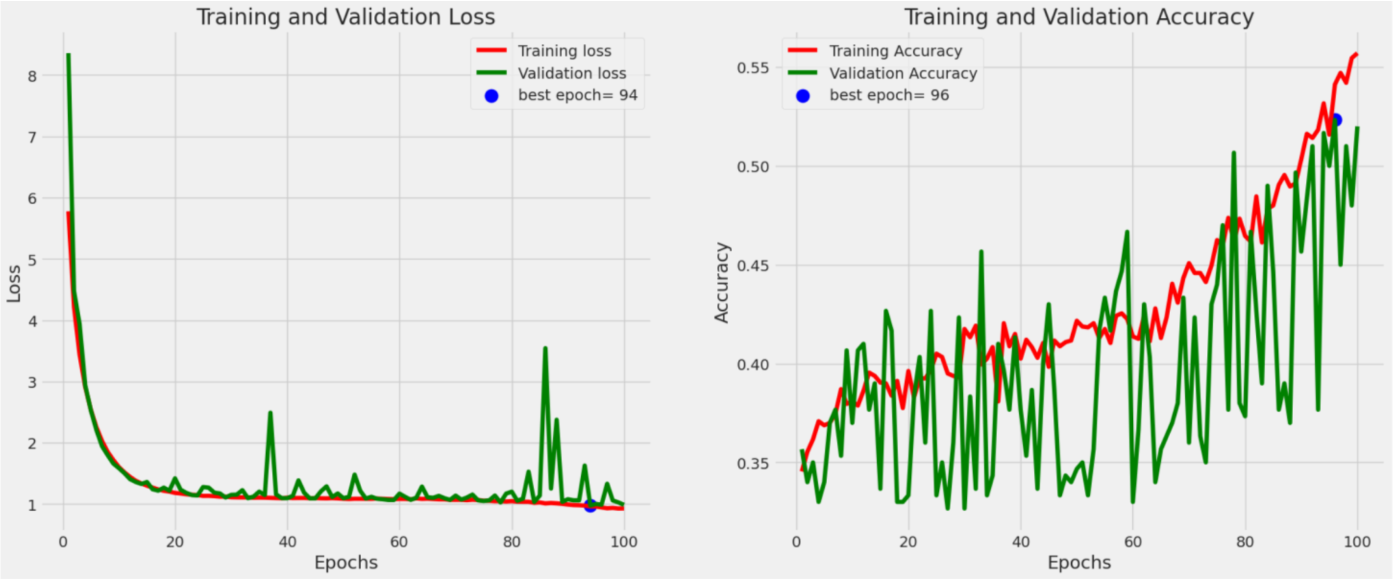

Figure S46. Confusion Matrix for Actual and Predicted U1-L1 values by VGG19

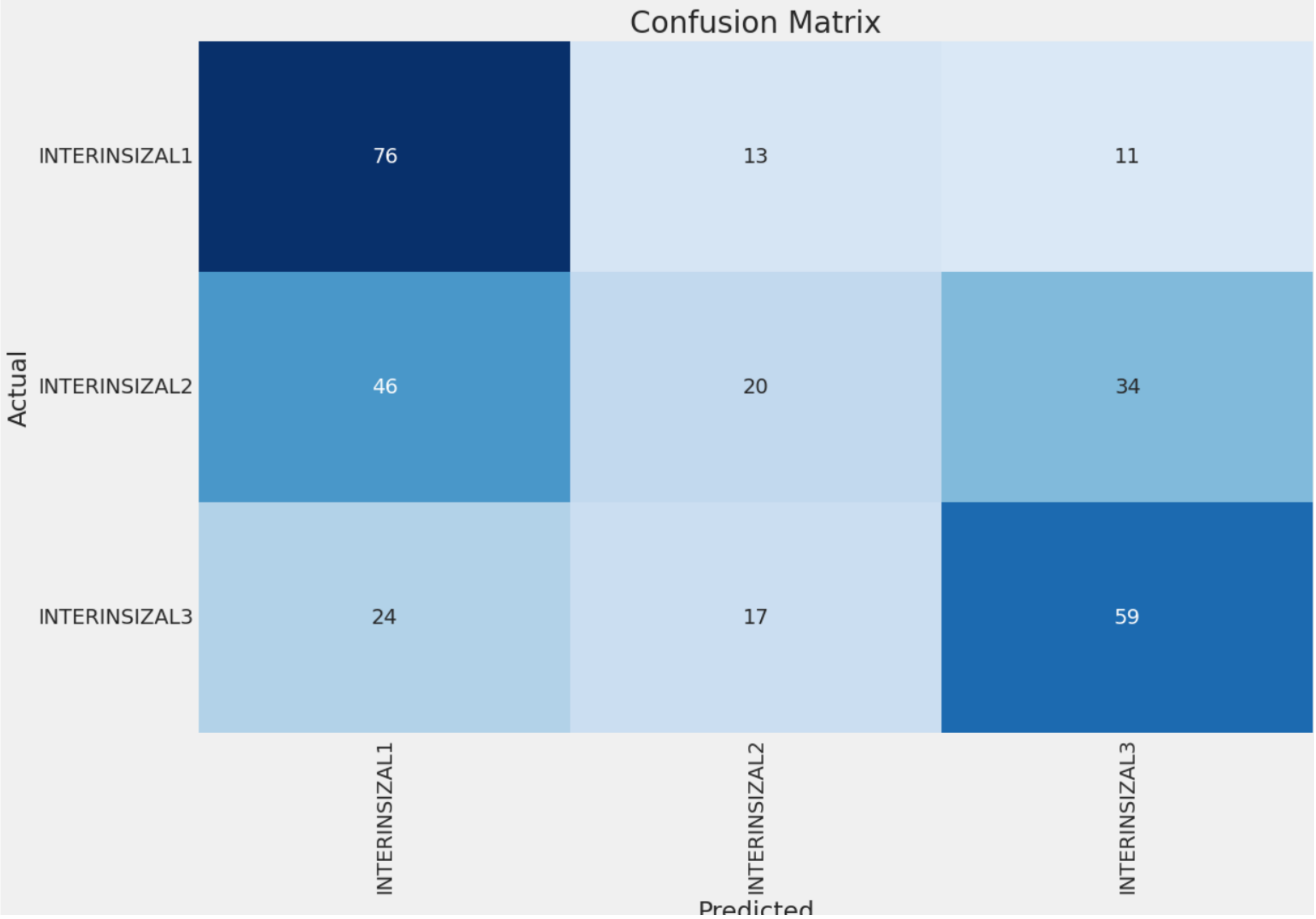

Table S25. Classification Report for U1-L1 by VGG19

	precision	recall	f1-score	support
INTERINSIZAL1	0.5205	0.7600	0.6179	100
INTERINSIZAL2	0.4000	0.2000	0.2667	100
INTERINSIZAL3	0.5673	0.5900	0.5784	100
accuracy			0.5167	300
macro avg	0.4960	0.5167	0.4877	300
weighted avg	0.4960	0.5167	0.4877	300

Figure S47. Training and Validation Loss and Training and Accuracy Graphs for NASNETMOBILE

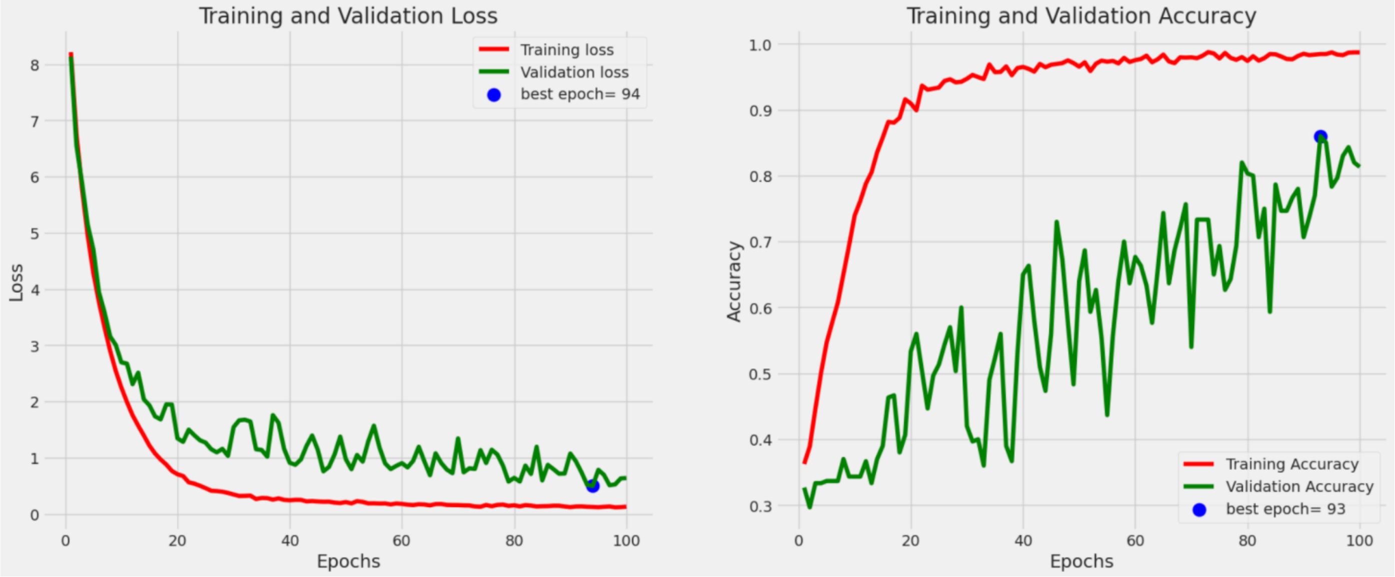

Figure S48. Confusion Matrix for Actual and Predicted U1-L1 values by NASNETMOBILE

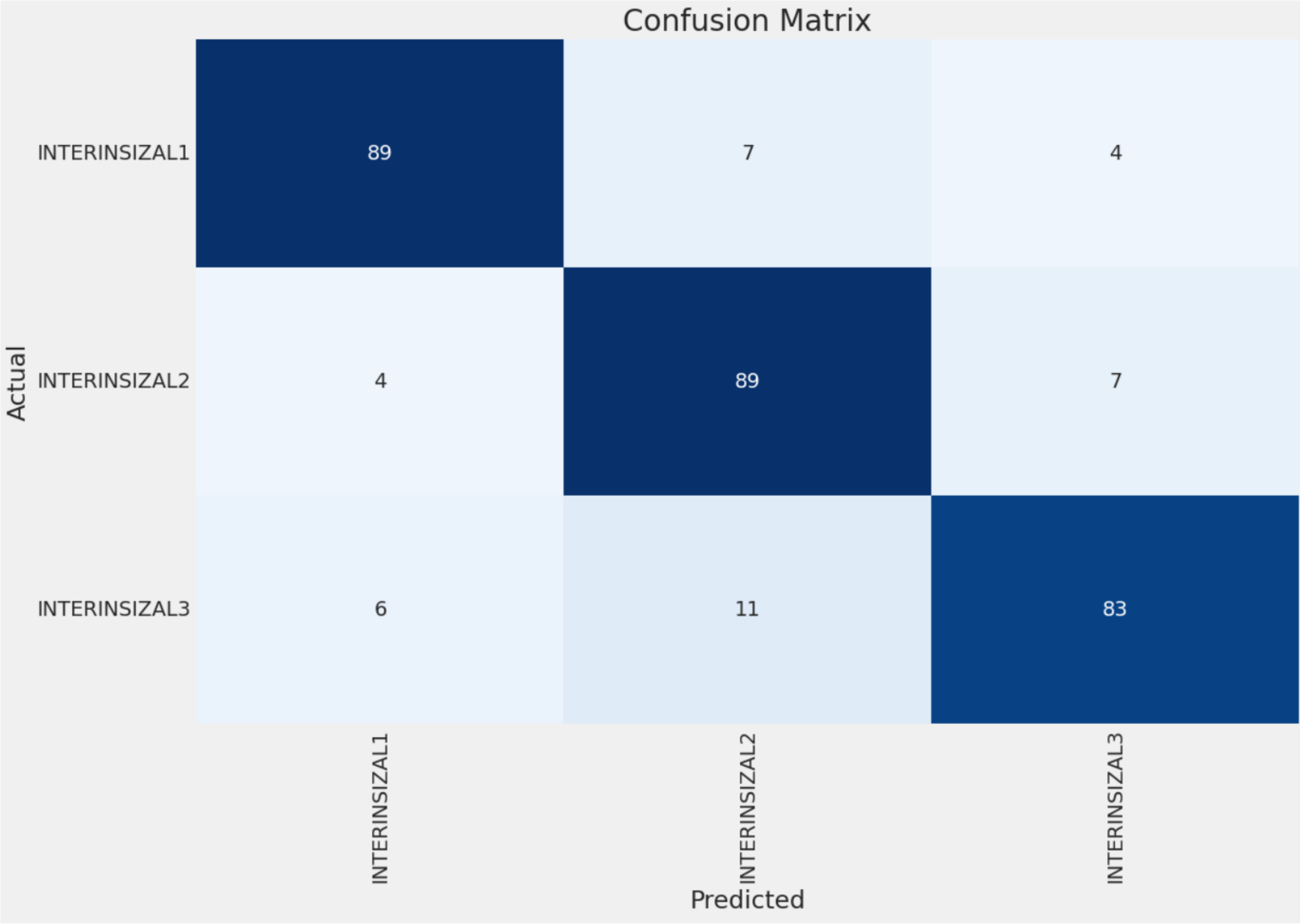

Table S26. Classification Report for U1-L1 by NASNETMOBILE

	precision	recall	f1-score	support
INTERINSIZAL1	0.8990	0.8900	0.8945	100
INTERINSIZAL2	0.8318	0.8900	0.8599	100
INTERINSIZAL3	0.8830	0.8300	0.8557	100

accuracy			0.8700	300
macro avg	0.8712	0.8700	0.8700	300
weighted avg	0.8712	0.8700	0.8700	300

Figure S49. Training and Validation Loss and Training and Accuracy Graphs for RESNET101

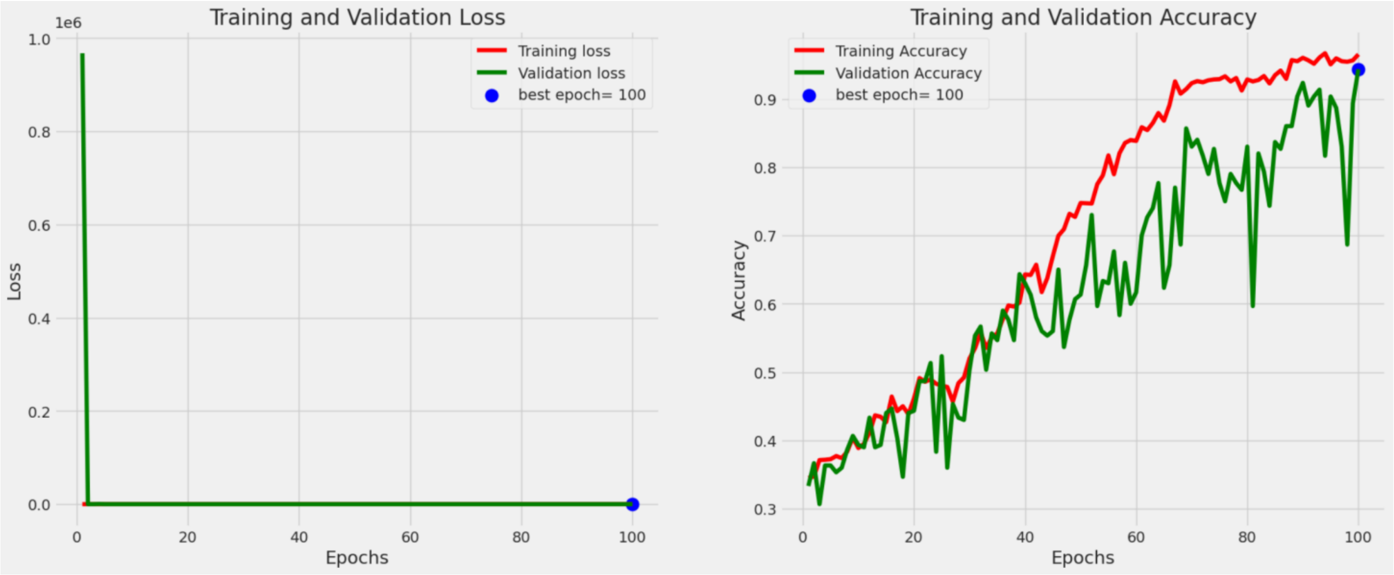

Figure S50. Confusion Matrix for Actual and Predicted U1-L1 values by RESNET101

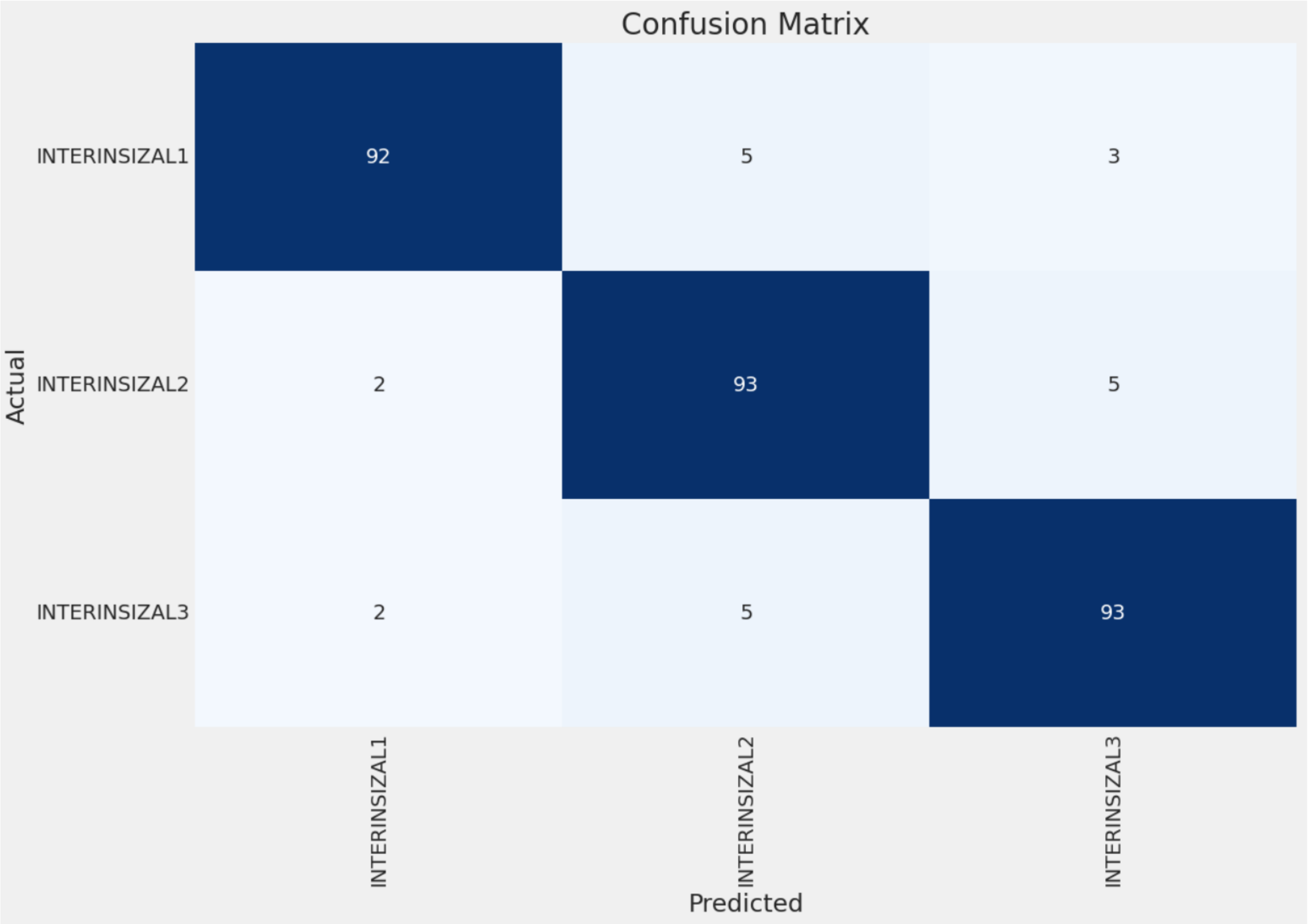

Table S27. Classification Report for U1-L1 by RESNET101

	precision	recall	f1-score	support
INTERINSIZAL1	0.9583	0.9200	0.9388	100
INTERINSIZAL2	0.9029	0.9300	0.9163	100
INTERINSIZAL3	0.9208	0.9300	0.9254	100
accuracy			0.9267	300
macro avg	0.9273	0.9267	0.9268	300
weighted avg	0.9273	0.9267	0.9268	300

Figure S51. Training and Validation Loss and Training and Accuracy Graphs for RESNET152

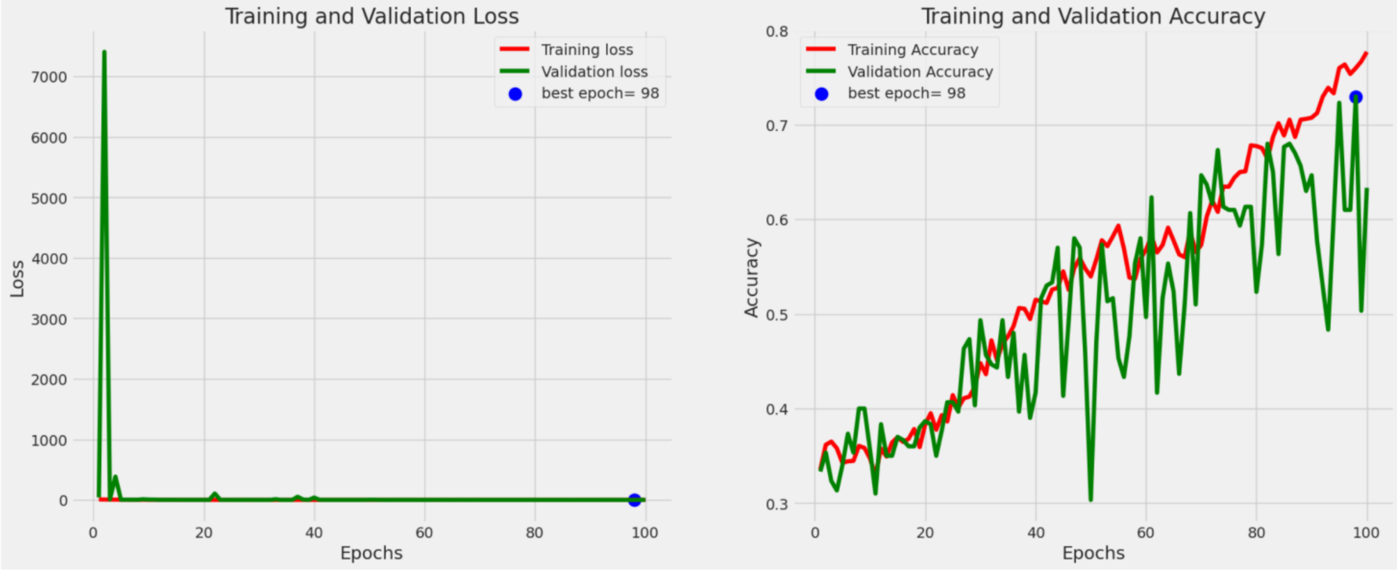

Figure S52. Confusion Matrix for Actual and Predicted U1-L1 values by RESNET152

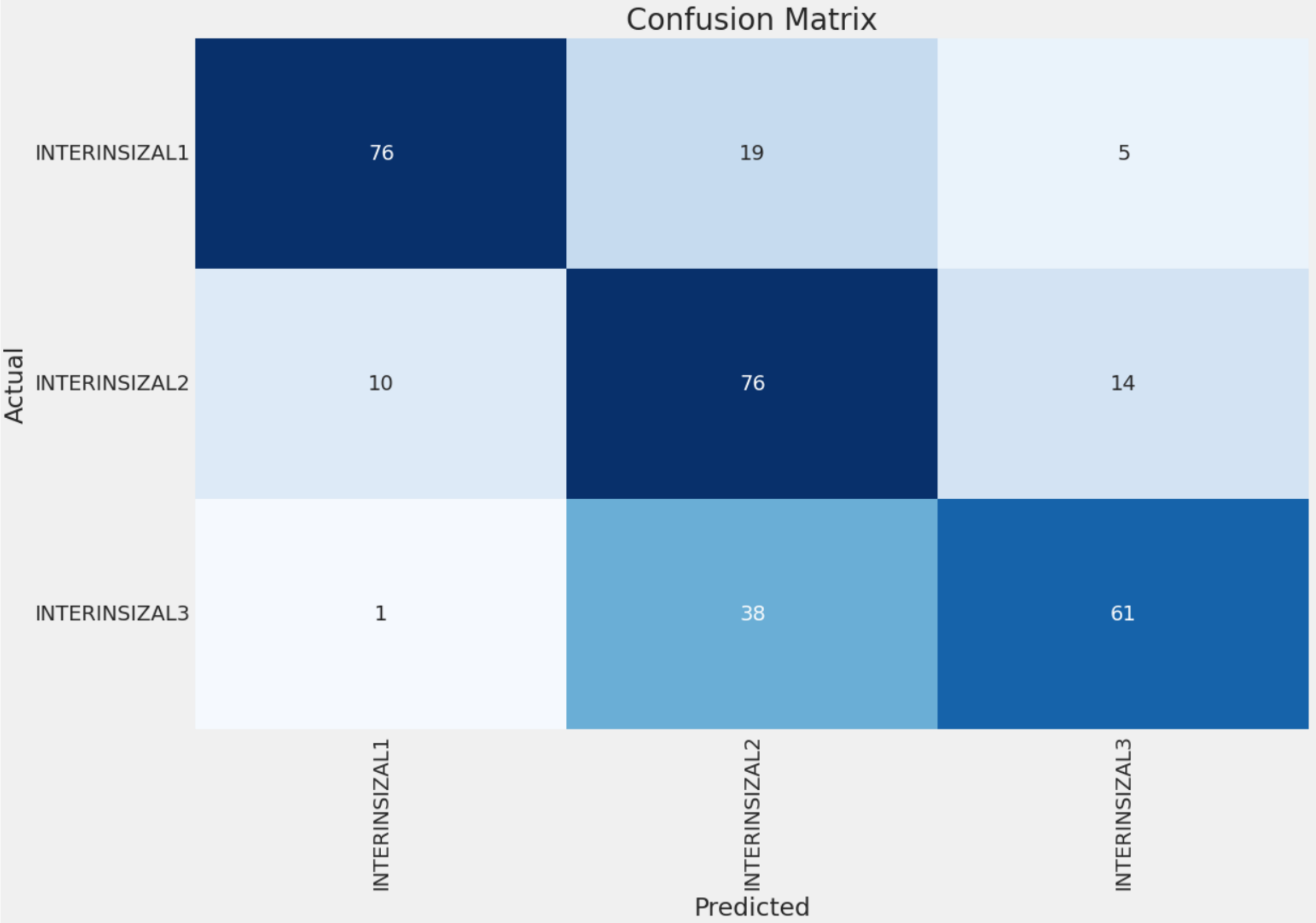

Table S28. Classification Report for U1-L1 by RESNET152

	precision	recall	f1-score	support
INTERINSIZAL1	0.8736	0.7600	0.8128	100
INTERINSIZAL2	0.5714	0.7600	0.6524	100
INTERINSIZAL3	0.7625	0.6100	0.6778	100
accuracy			0.7100	300
macro avg	0.7358	0.7100	0.7143	300

```
weighted avg      0.7358     0.7100     0.7143        300
```

Figure S53. Training and Validation Loss and Training and Accuracy Graphs for RESNET50

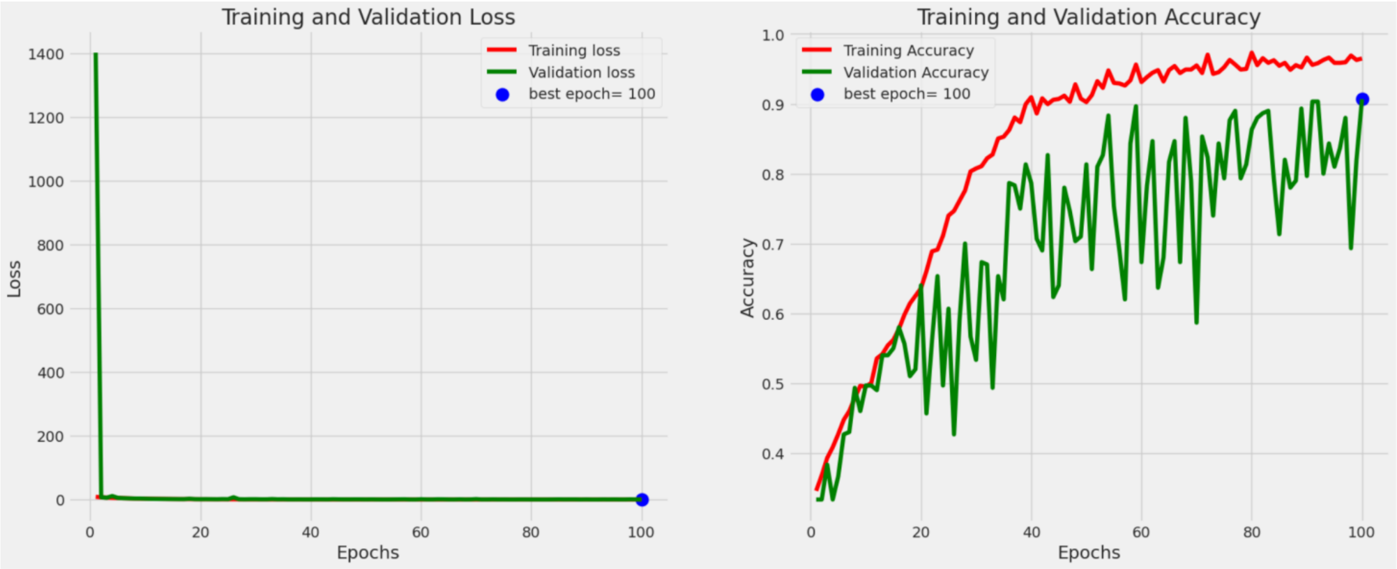

Figure S54. Confusion Matrix for Actual and Predicted U1-L1 values by RESNET50

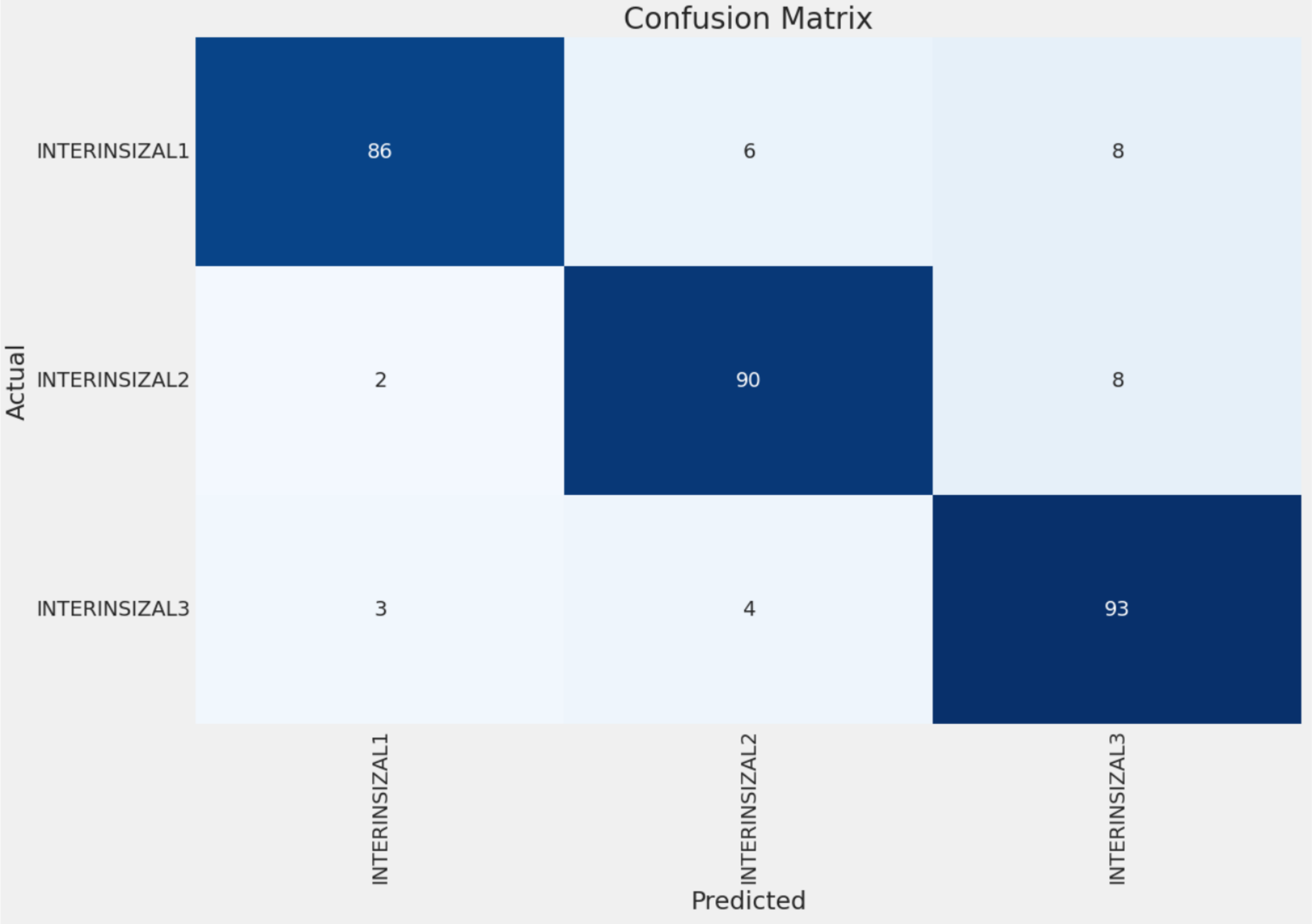

Table S29. Classification Report for U1-L1 by RESNET50

	precision	recall	f1-score	support
INTERINSIZAL1	0.9451	0.8600	0.9005	100
INTERINSIZAL2	0.9000	0.9000	0.9000	100
INTERINSIZAL3	0.8532	0.9300	0.8900	100
accuracy			0.8967	300
macro avg	0.8994	0.8967	0.8968	300
weighted avg	0.8994	0.8967	0.8968	300

Figure S55. Training and Validation Loss and Training and Accuracy Graphs for EFFICIENTNET V2

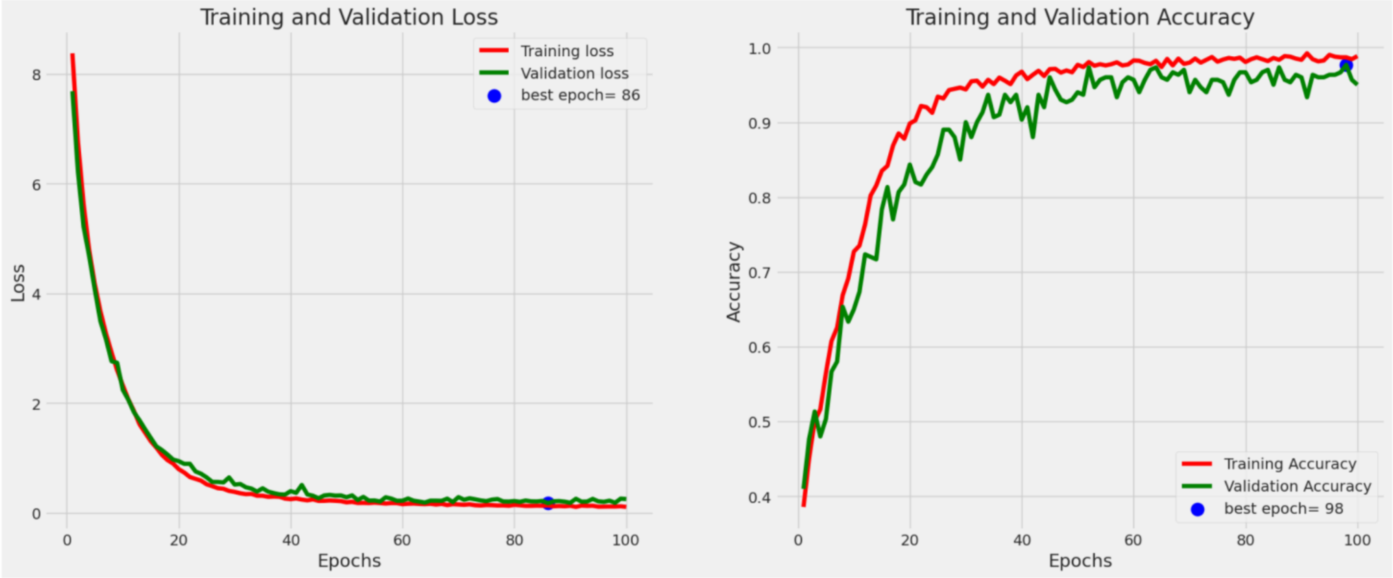

Figure S56. Confusion Matrix for Actual and Predicted U1-L1 values by EFFICIENTNET V2

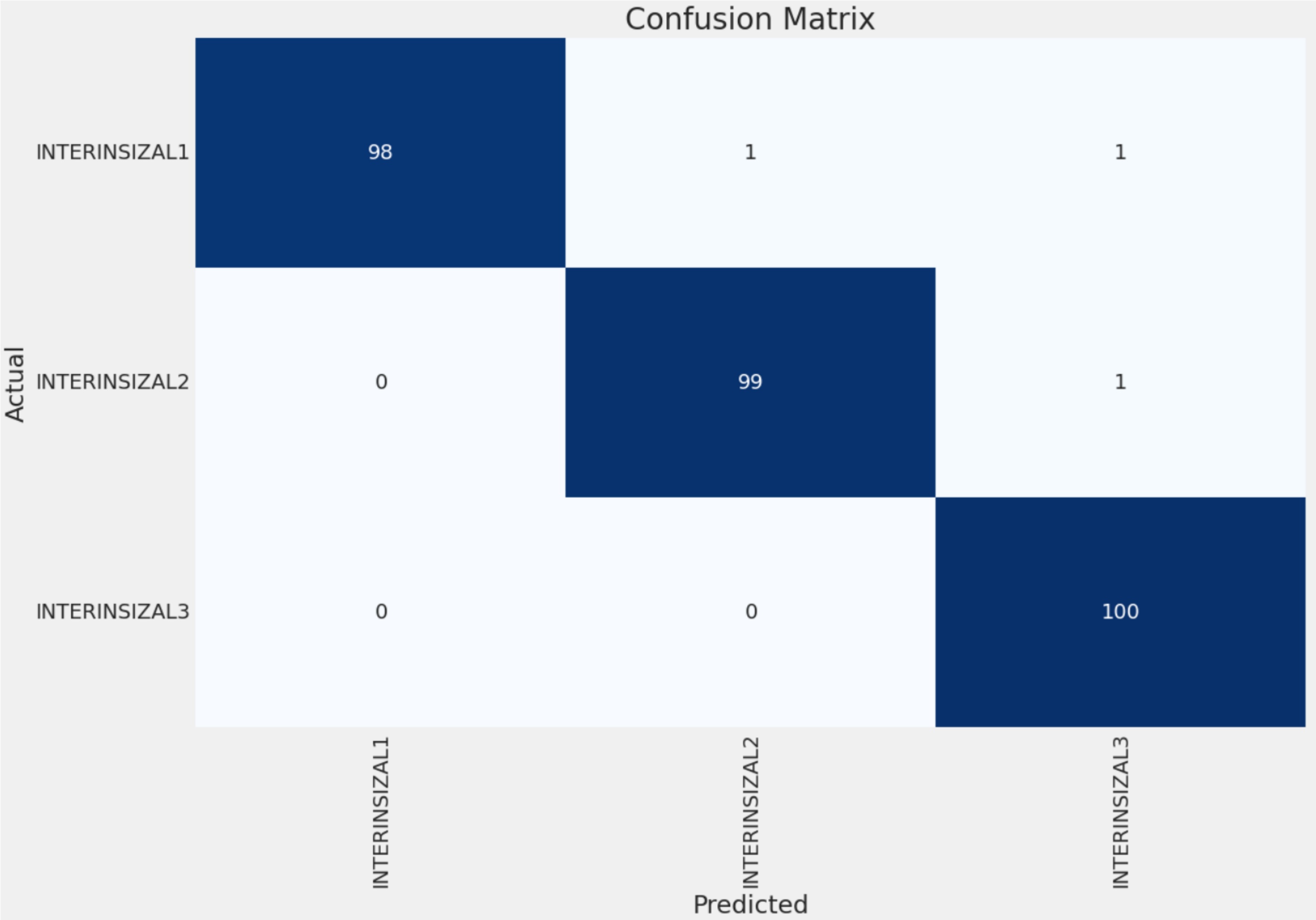

Table S30. Classification Report for U1-L1 by EFFICIENTNET V2

----

	precision	recall	f1-score	support
INTERINSIZAL1	1.0000	0.9800	0.9899	100
INTERINSIZAL2	0.9900	0.9900	0.9900	100
INTERINSIZAL3	0.9804	1.0000	0.9901	100

```
    accuracy                           0.9900       300
   macro avg     0.9901    0.9900    0.9900       300
weighted avg     0.9901    0.9900    0.9900       300
```

Table S31. Summary table for accuracy values of U1-PP Classification

**U1-PP**	
MOBILENET V2	91.67
INCEPTION V3	**33.67 (NOT EVALUATED)**
DENSENET 121	96.00
DENSENET 169	96.33 (GRAFİK)
DENSENET 201	96.00 (GRAFİK)
EFFICIENTNET B0	96.67
XCEPTION	**33.33 (NOT EVALUATED)**
VGG16	**35 (NOT EVALUATED)**
VGG19	**34.67 (NOT EVALUATED)**
NASNETMOBILE	81.00
RESNET101	**36.67 (NOT EVALUATED)**
RESNET152	**34.33 (NOT EVALUATED)**
RESNET50	87.67
EFFICIENTNET V2	94.67

Figure S57. Training and Validation Loss and Training and Accuracy Graphs for MOBILENET V2

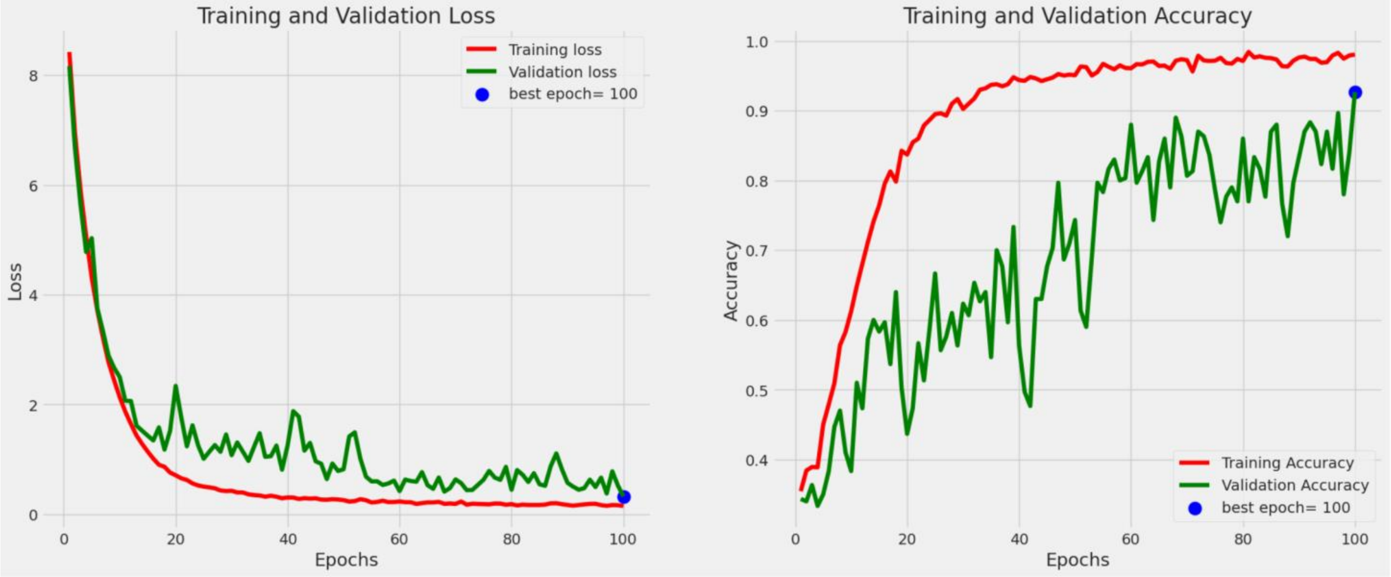

Figure S58. Confusion Matrix for Actual and Predicted U1-PP values by MOBILENET V2

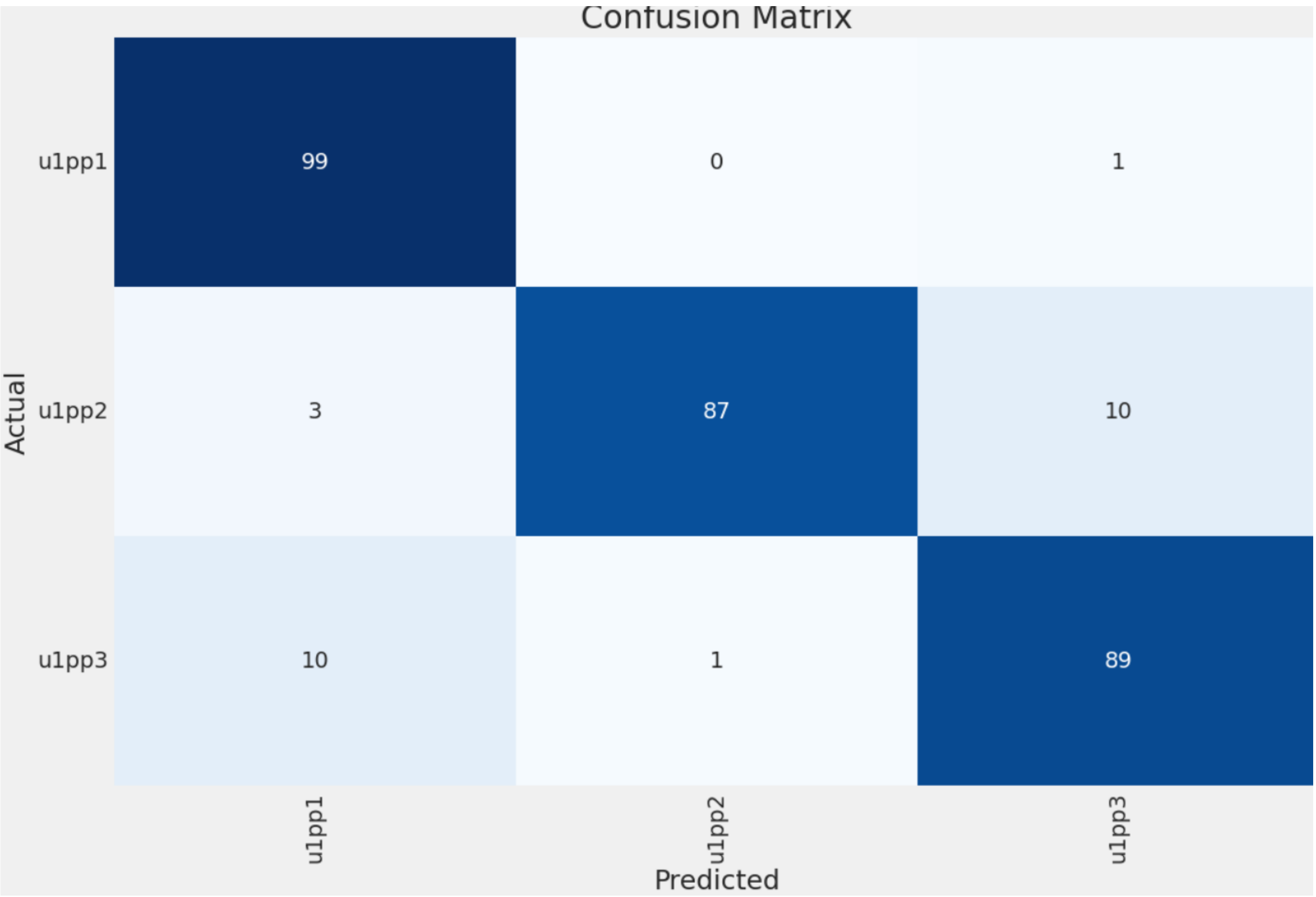

Table S32. Classification Report for U1-PP by MOBILENET V2

	precision	recall	f1-score	support
u1pp1	0.8839	0.9900	0.9340	100
u1pp2	0.9886	0.8700	0.9255	100
u1pp3	0.8900	0.8900	0.8900	100

accuracy			0.9167	300
macro avg	0.9209	0.9167	0.9165	300
weighted avg	0.9209	0.9167	0.9165	300

Figure S59. Training and Validation Loss and Training and Accuracy Graphs for DENSENET 121

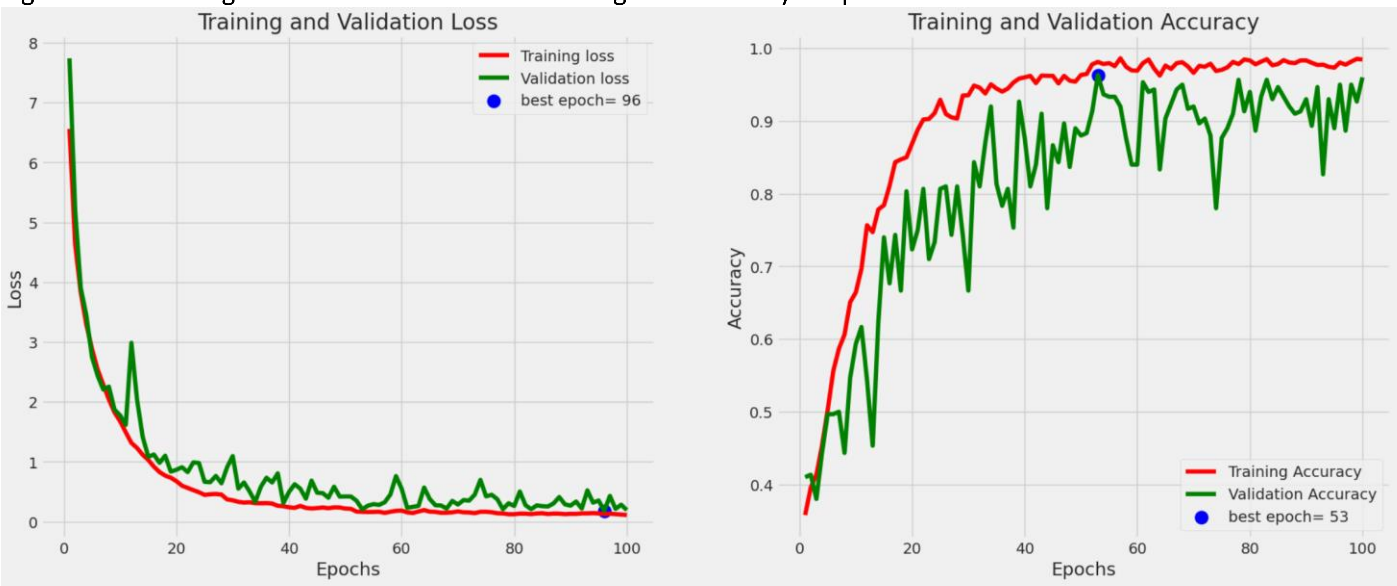

Figure S60. Confusion Matrix for Actual and Predicted U1-PP values by DENSENET 121

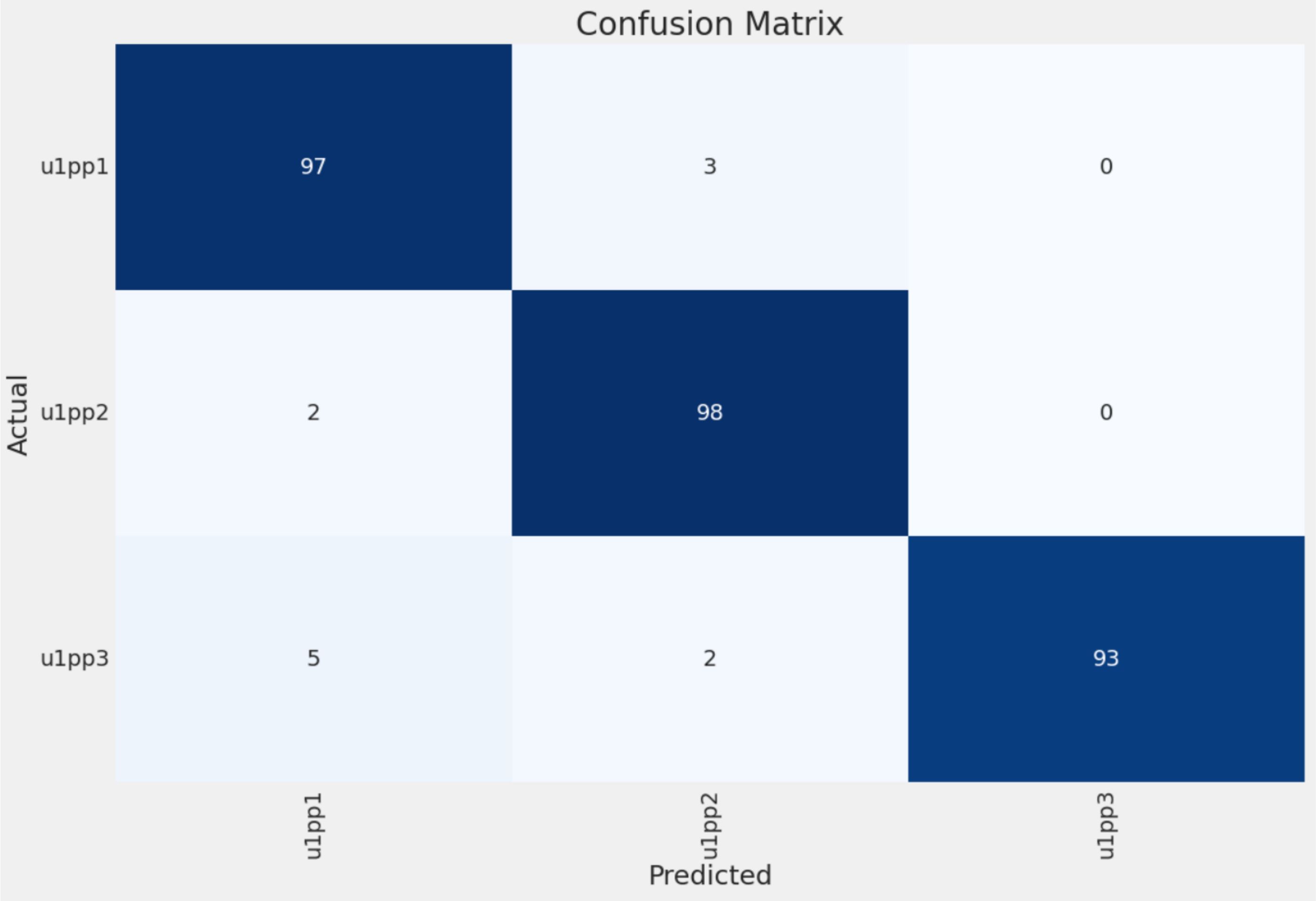

Table S33. Classification Report for U1-PP by DENSENET 121

	precision	recall	f1-score	support
u1pp1	0.9327	0.9700	0.9510	100
u1pp2	0.9515	0.9800	0.9655	100
u1pp3	1.0000	0.9300	0.9637	100
accuracy			0.9600	300
macro avg	0.9614	0.9600	0.9601	300
weighted avg	0.9614	0.9600	0.9601	300

Figure S61. Training and Validation Loss and Training and Accuracy Graphs for DENSENET 169

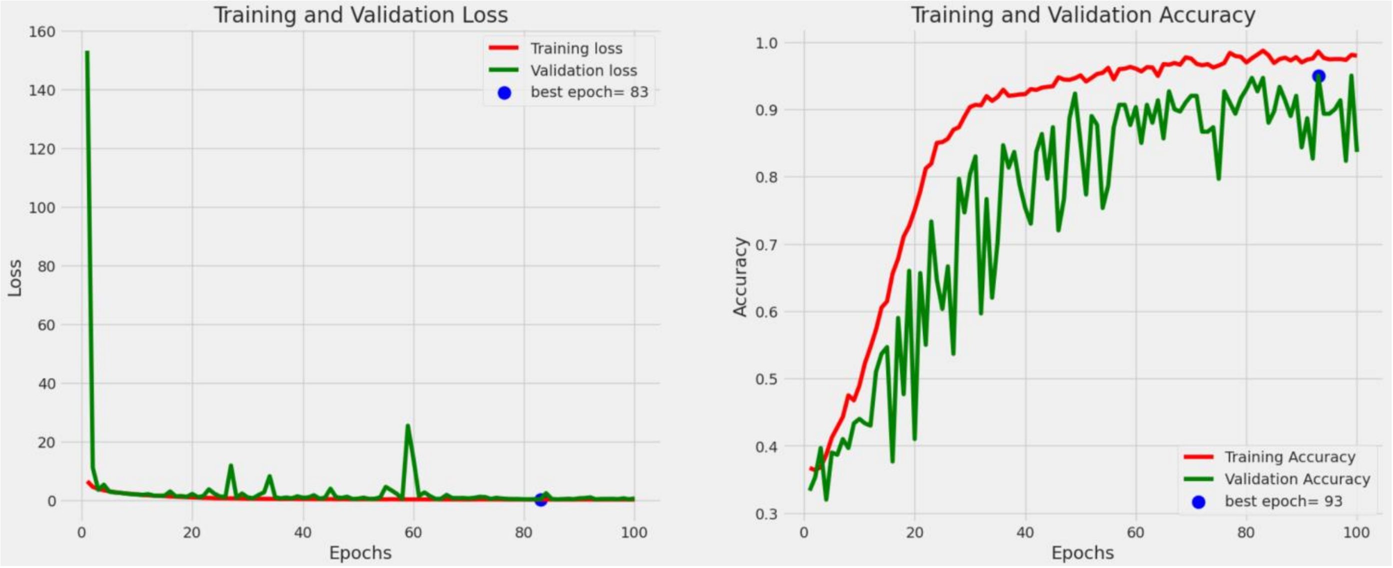

Figure S62. Confusion Matrix for Actual and Predicted U1-PP values by DENSENET 169

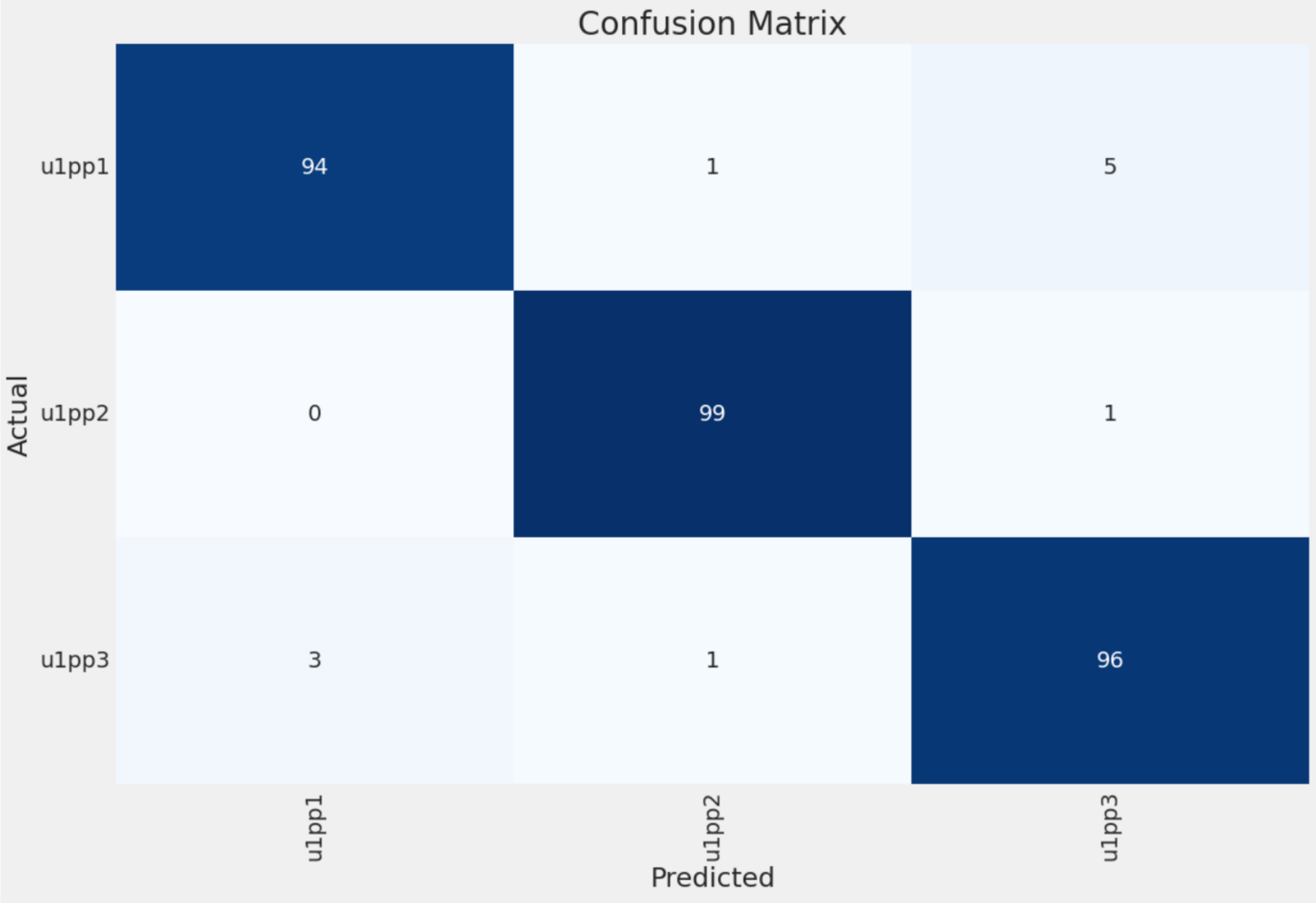

Table S34. Classification Report for U1-PP by DENSENET 169

----

	precision	recall	f1-score	support
u1pp1	0.9691	0.9400	0.9543	100
u1pp2	0.9802	0.9900	0.9851	100
u1pp3	0.9412	0.9600	0.9505	100
accuracy			0.9633	300
macro avg	0.9635	0.9633	0.9633	300

weighted avg	0.9635	0.9633	0.9633	300

Figure S63. Training and Validation Loss and Training and Accuracy Graphs for DENSENET 201

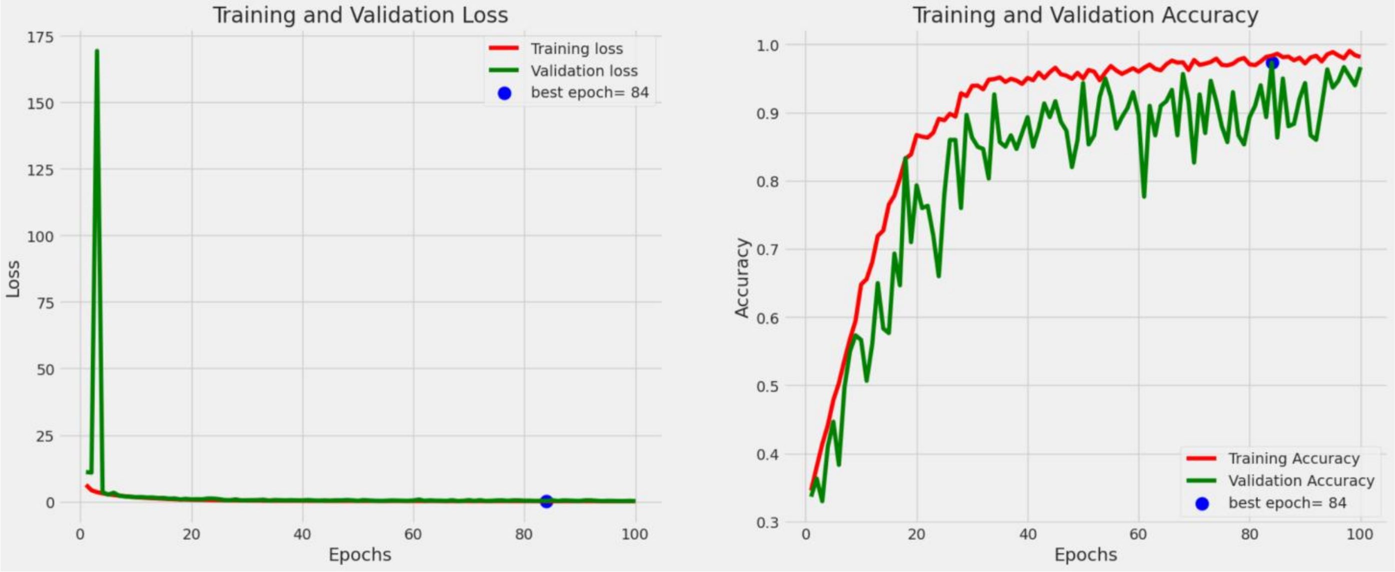

Figure S64. Confusion Matrix for Actual and Predicted U1-PP values by DENSENET 201

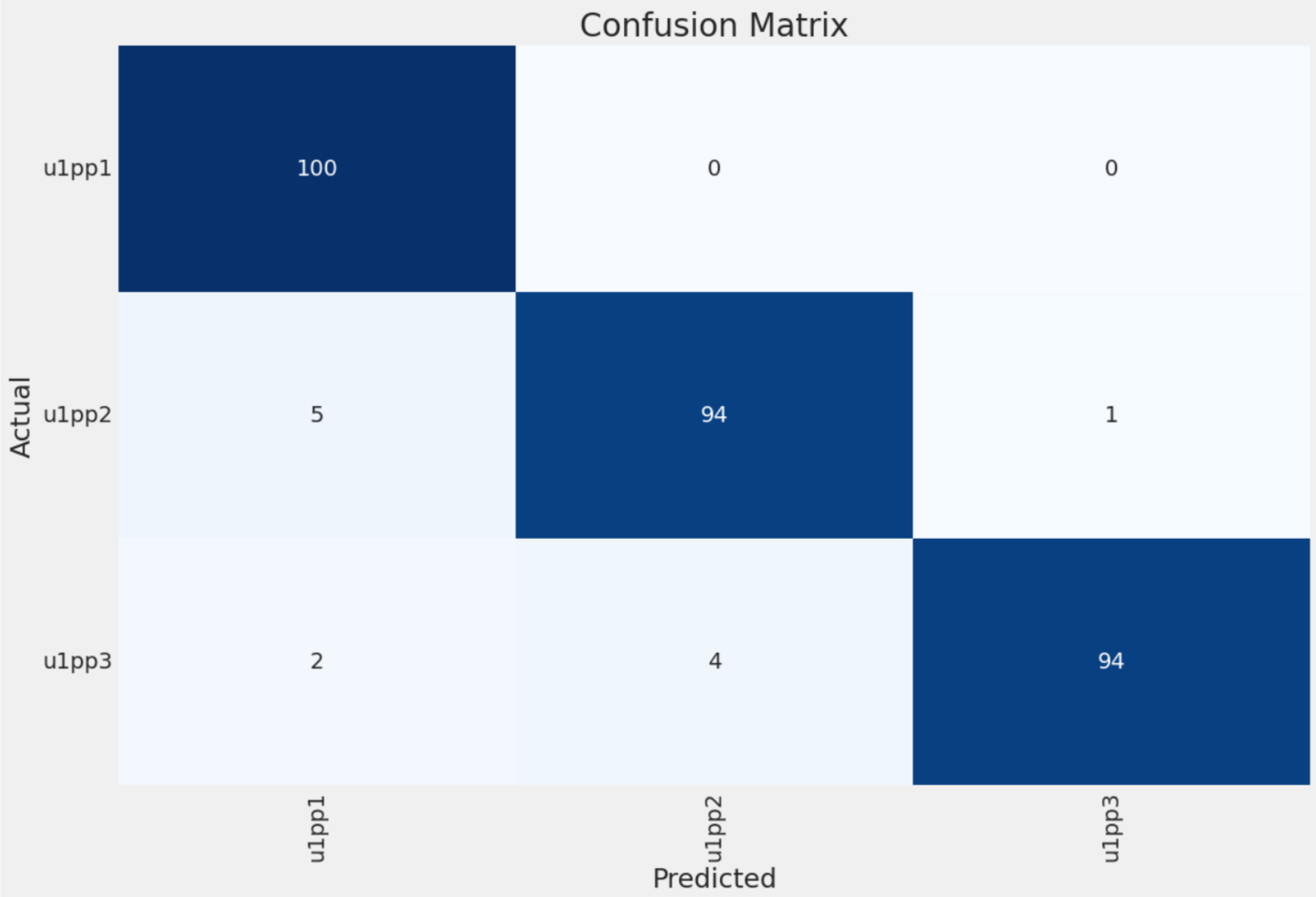

Table S35. Classification Report for U1-PP by DENSENET 201

	precision	recall	f1-score	support
u1pp1	0.9346	1.0000	0.9662	100
u1pp2	0.9592	0.9400	0.9495	100
u1pp3	0.9895	0.9400	0.9641	100
accuracy			0.9600	300
macro avg	0.9611	0.9600	0.9599	300
weighted avg	0.9611	0.9600	0.9599	300

Figure S65. Training and Validation Loss and Training and Accuracy Graphs for EFFICIENTNET B0

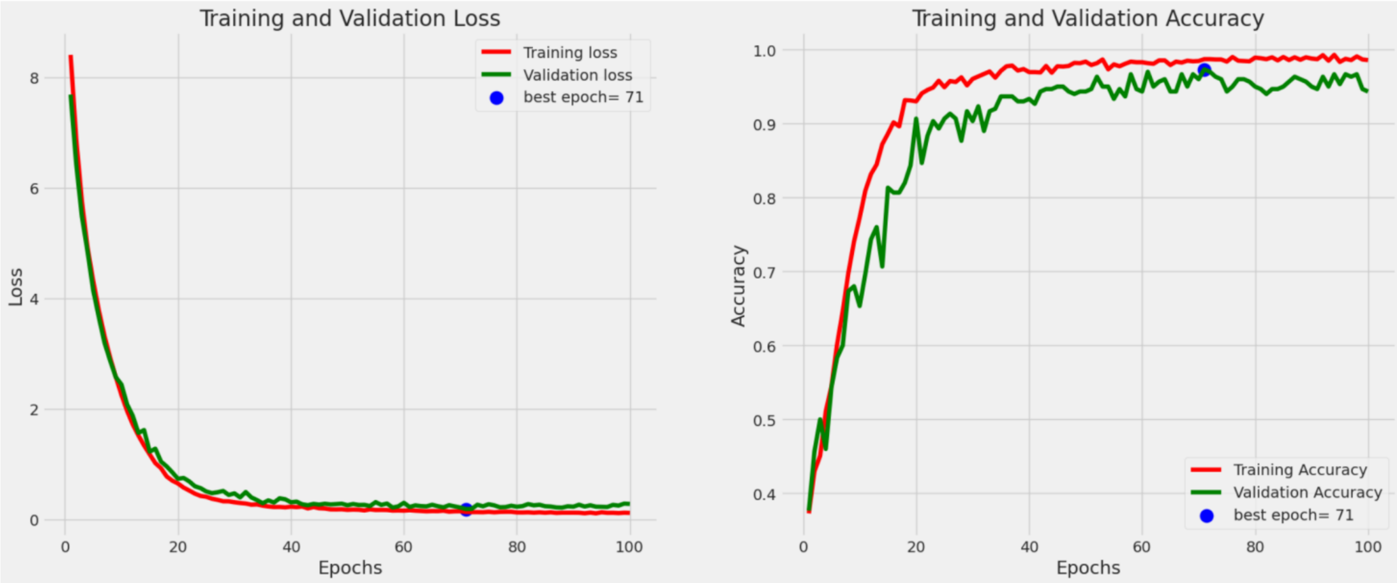

Figure S66. Confusion Matrix for Actual and Predicted U1-PP values by EFFICIENTNET B0

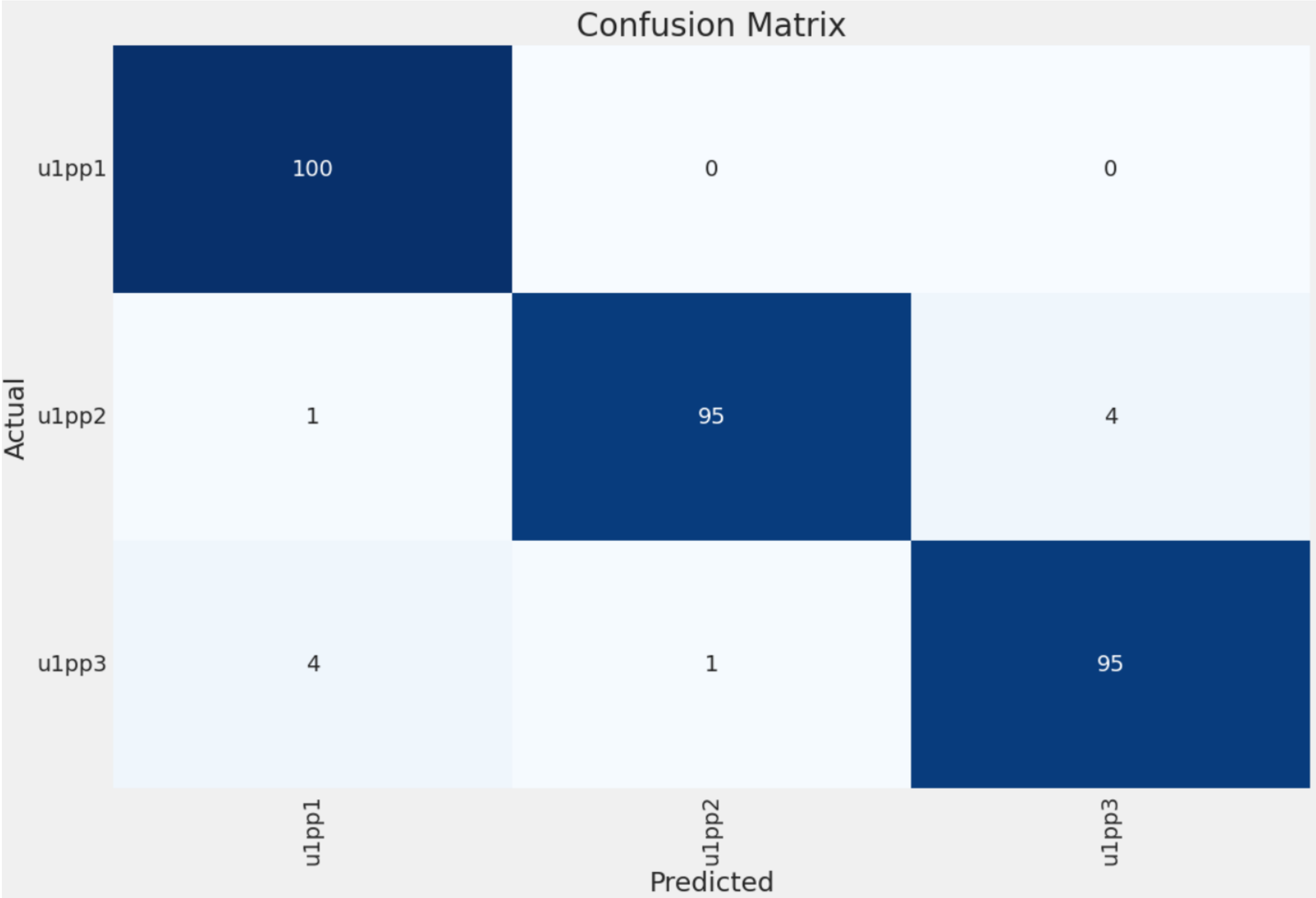

Table S36. Classification Report for U1-PP by EFFICIENTNET B0

	precision	recall	f1-score	support
u1pp1	0.9524	1.0000	0.9756	100
u1pp2	0.9896	0.9500	0.9694	100
u1pp3	0.9596	0.9500	0.9548	100
accuracy			0.9667	300
macro avg	0.9672	0.9667	0.9666	300

weighted avg	0.9672	0.9667	0.9666	300

Figure S67. Training and Validation Loss and Training and Accuracy Graphs for NASNETMOBILE

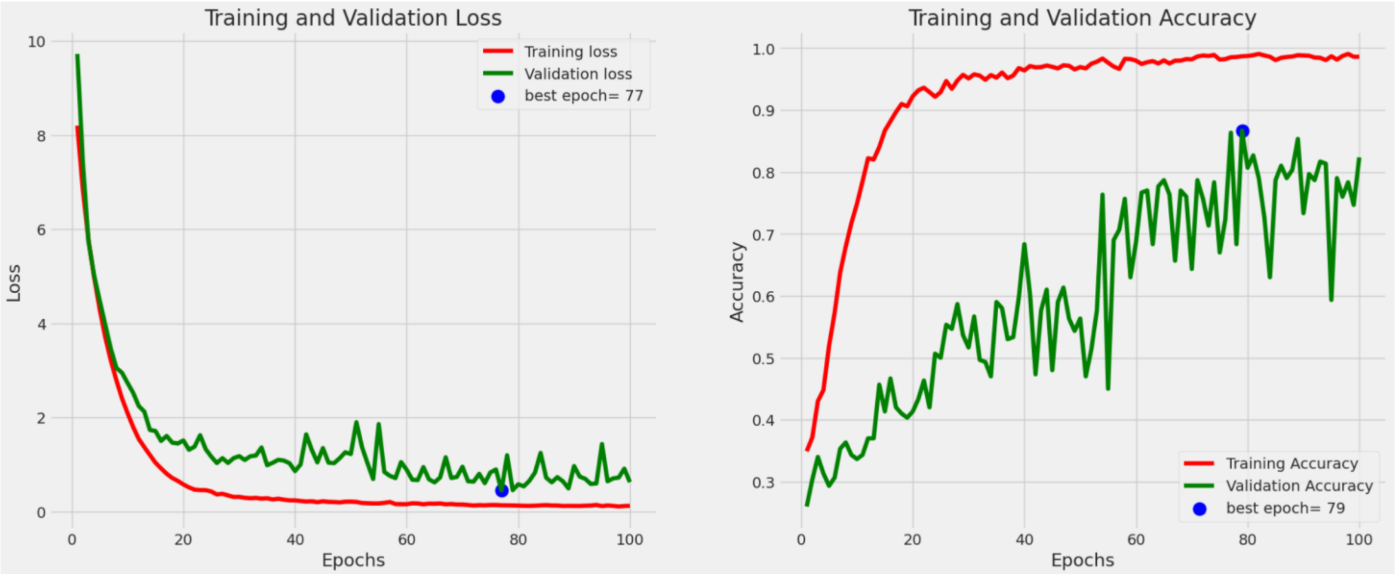

Figure S68. Confusion Matrix for Actual and Predicted U1-PP values by NASNETMOBILE

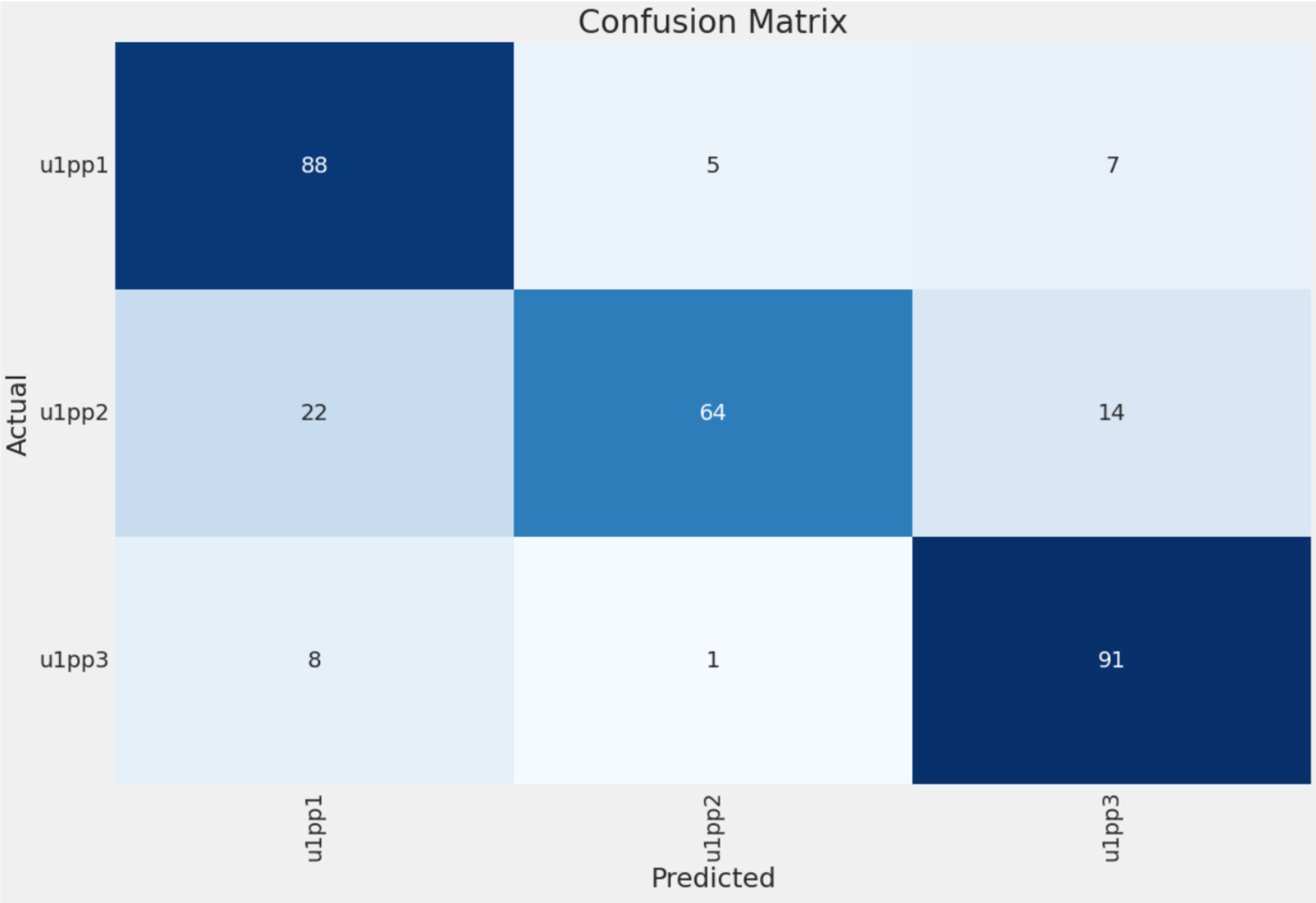

Table S37. Classification Report for U1-PP by NASNETMOBILE

	precision	recall	f1-score	support
u1pp1	0.7458	0.8800	0.8073	100
u1pp2	0.9143	0.6400	0.7529	100
u1pp3	0.8125	0.9100	0.8585	100
accuracy			0.8100	300
macro avg	0.8242	0.8100	0.8063	300
weighted avg	0.8242	0.8100	0.8063	300

Figure S69. Training and Validation Loss and Training and Accuracy Graphs for RESNET50

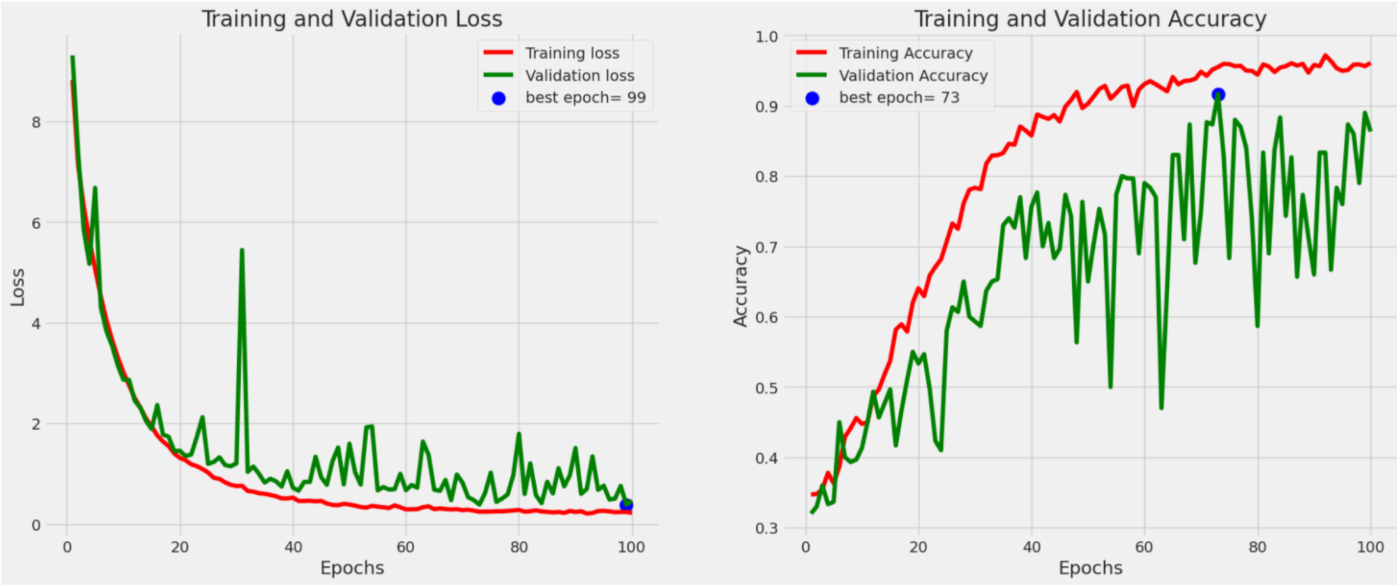

Figure S70. Confusion Matrix for Actual and Predicted U1-PP values by RESNET50

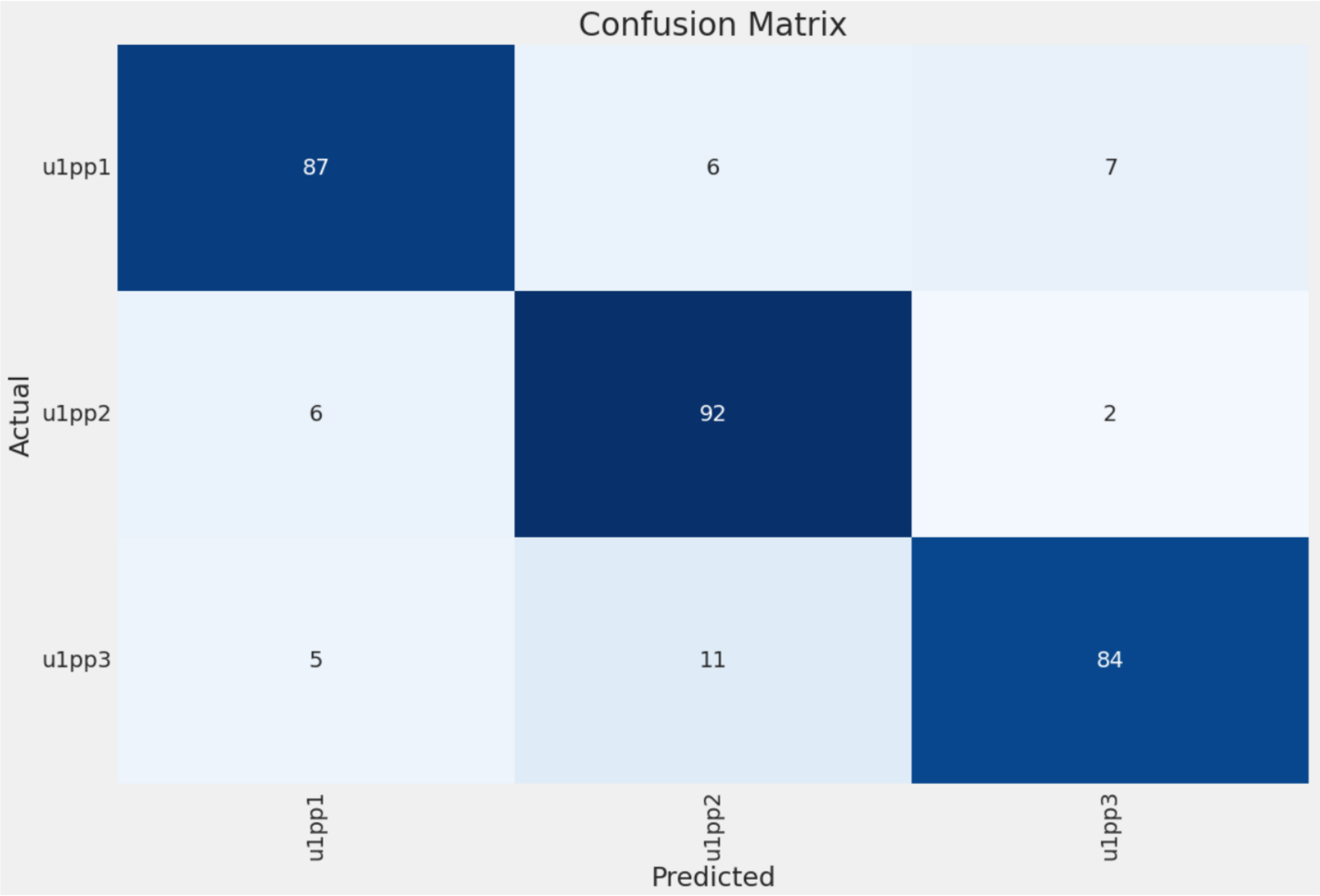

Table S38. Classification Report for U1-PP by RESNET50

----

	precision	recall	f1-score	support
u1pp1	0.8878	0.8700	0.8788	100
u1pp2	0.8440	0.9200	0.8804	100
u1pp3	0.9032	0.8400	0.8705	100

accuracy			0.8767	300
macro avg	0.8783	0.8767	0.8765	300
weighted avg	0.8783	0.8767	0.8765	300

Figure S71. Training and Validation Loss and Training and Accuracy Graphs for EFFICIENTNET V2

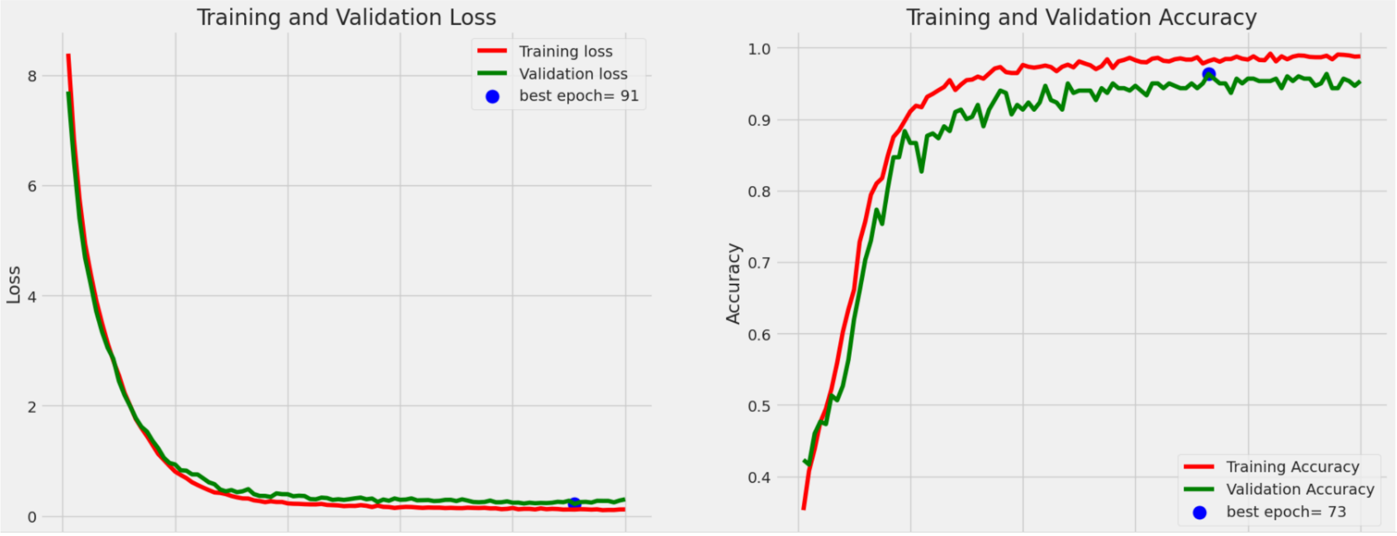

Figure S72. Confusion Matrix for Actual and Predicted U1-PP values by EFFICIENTNET V2

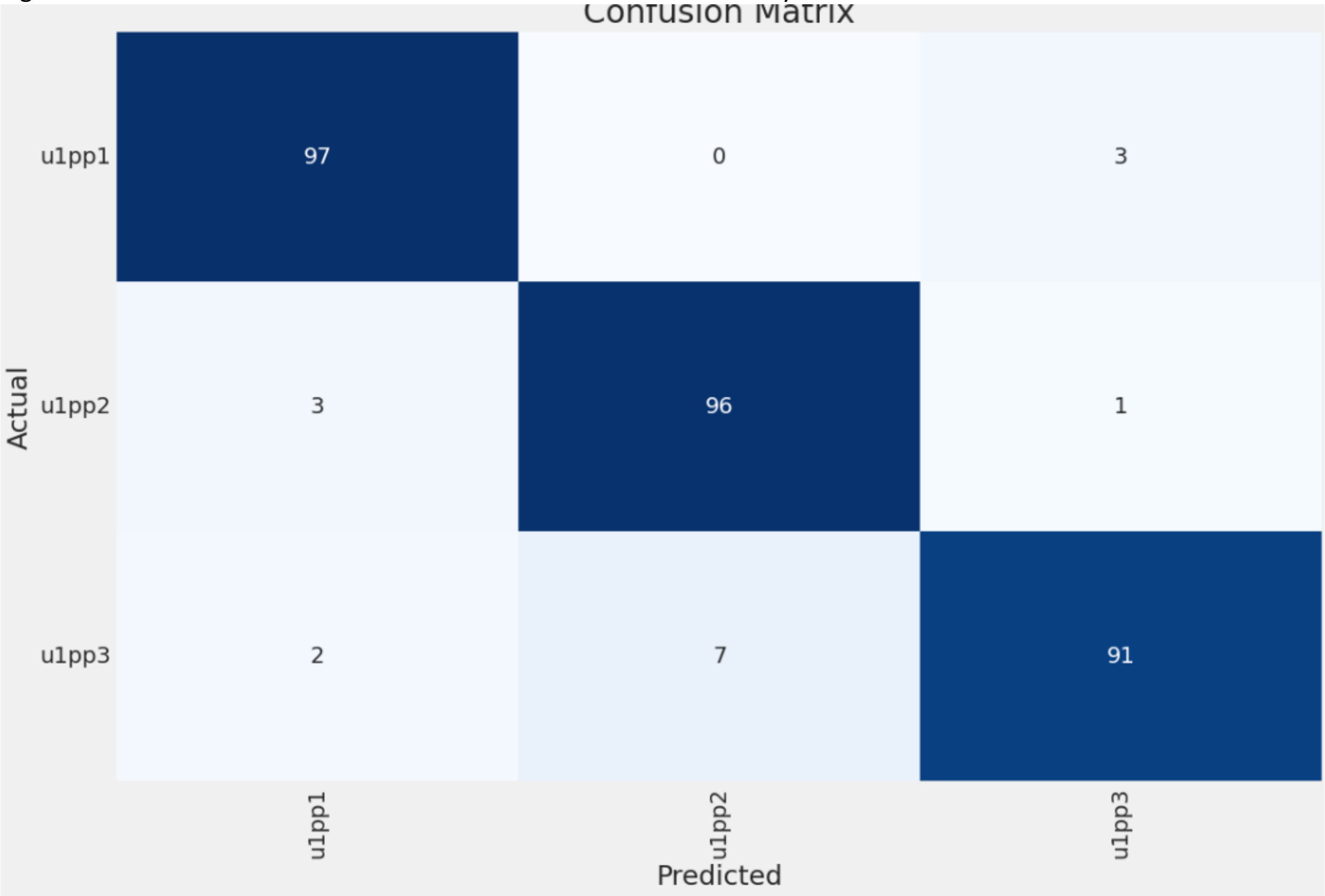

Table S39. Classification Report for U1-PP by EFFICIENTNET V2

	precision	recall	f1-score	support
u1pp1	0.9510	0.9700	0.9604	100
u1pp2	0.9320	0.9600	0.9458	100
u1pp3	0.9579	0.9100	0.9333	100
accuracy			0.9467	300
macro avg	0.9470	0.9467	0.9465	300
weighted avg	0.9470	0.9467	0.9465	300

Table S40. Summary table for accuracy values of WITS Classification

WITS	
MOBILENET V2	94.67
INCEPTION V3	96.67
DENSENET 121	97.00
DENSENET 169	96.00
DENSENET 201	98.00
EFFICIENTNET B0	98.33
XCEPTION	91.33
VGG16	88.33
VGG19	86.67 (GRAPH)
NASNETMOBILE	83.67
RESNET101	93.67 (GRAPH)
RESNET152	93.67 (GRAPH)
RESNET50	95.00 (GRAPH)
EFFICIENTNET V2	97.67

Figure S73. Training and Validation Loss and Training and Accuracy Graphs for MOBILENET V2

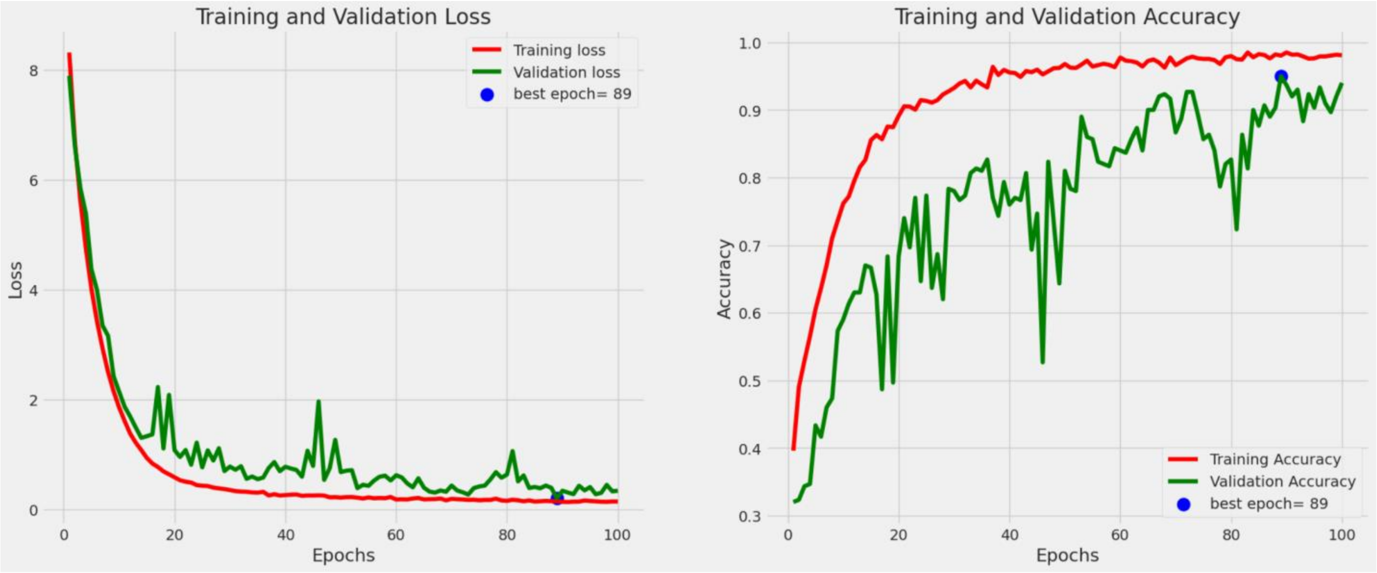

Figure S74. Confusion Matrix for Actual and Predicted WITS values by MOBILENET V2

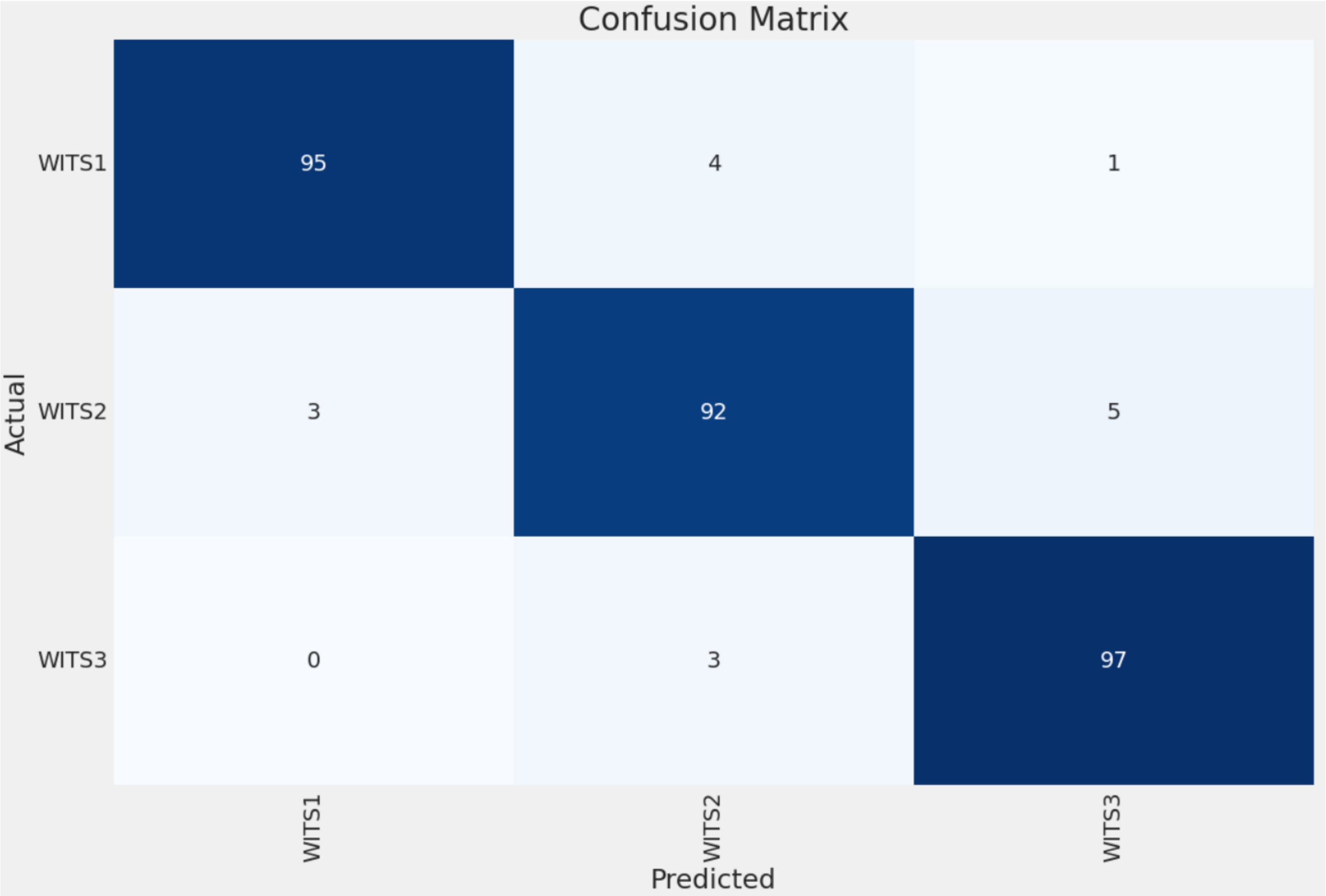

Table S41. Classification Report for WITS by MOBILENET V2

	precision	recall	f1-score	support
WITS1	0.9694	0.9500	0.9596	100
WITS2	0.9293	0.9200	0.9246	100
WITS3	0.9417	0.9700	0.9557	100
accuracy			0.9467	300
macro avg	0.9468	0.9467	0.9466	300
weighted avg	0.9468	0.9467	0.9466	300

Figure S75. Training and Validation Loss and Training and Accuracy Graphs for INCEPTION V3

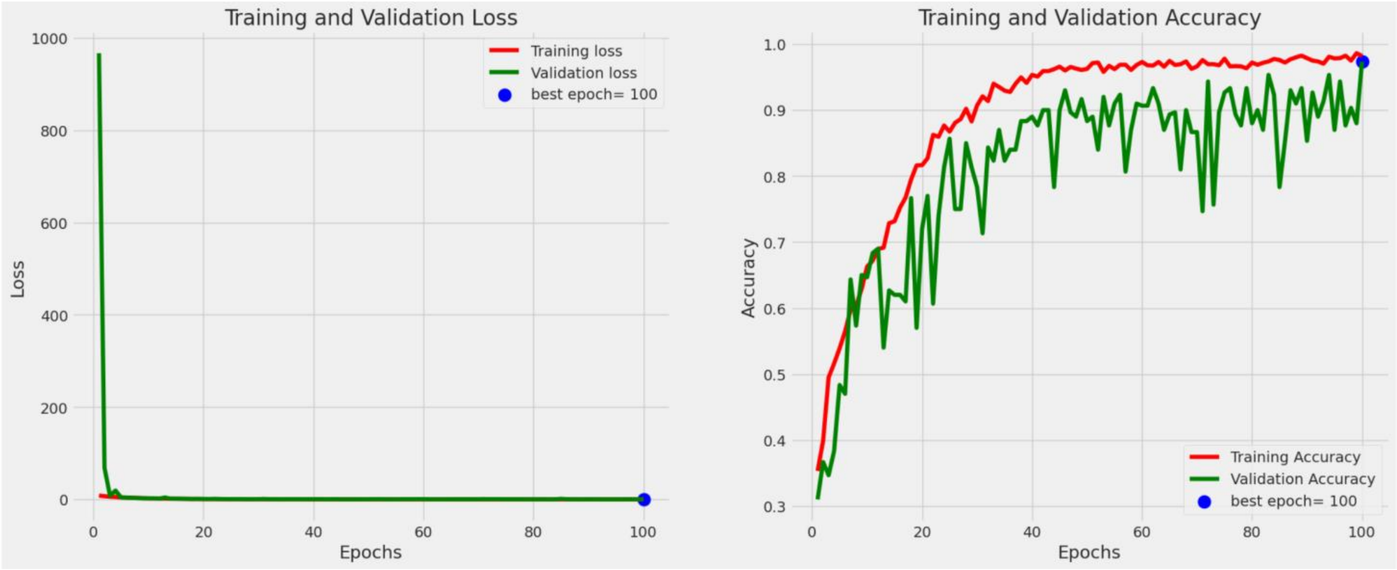

Figure S76. Confusion Matrix for Actual and Predicted WITS values by INCEPTION V3

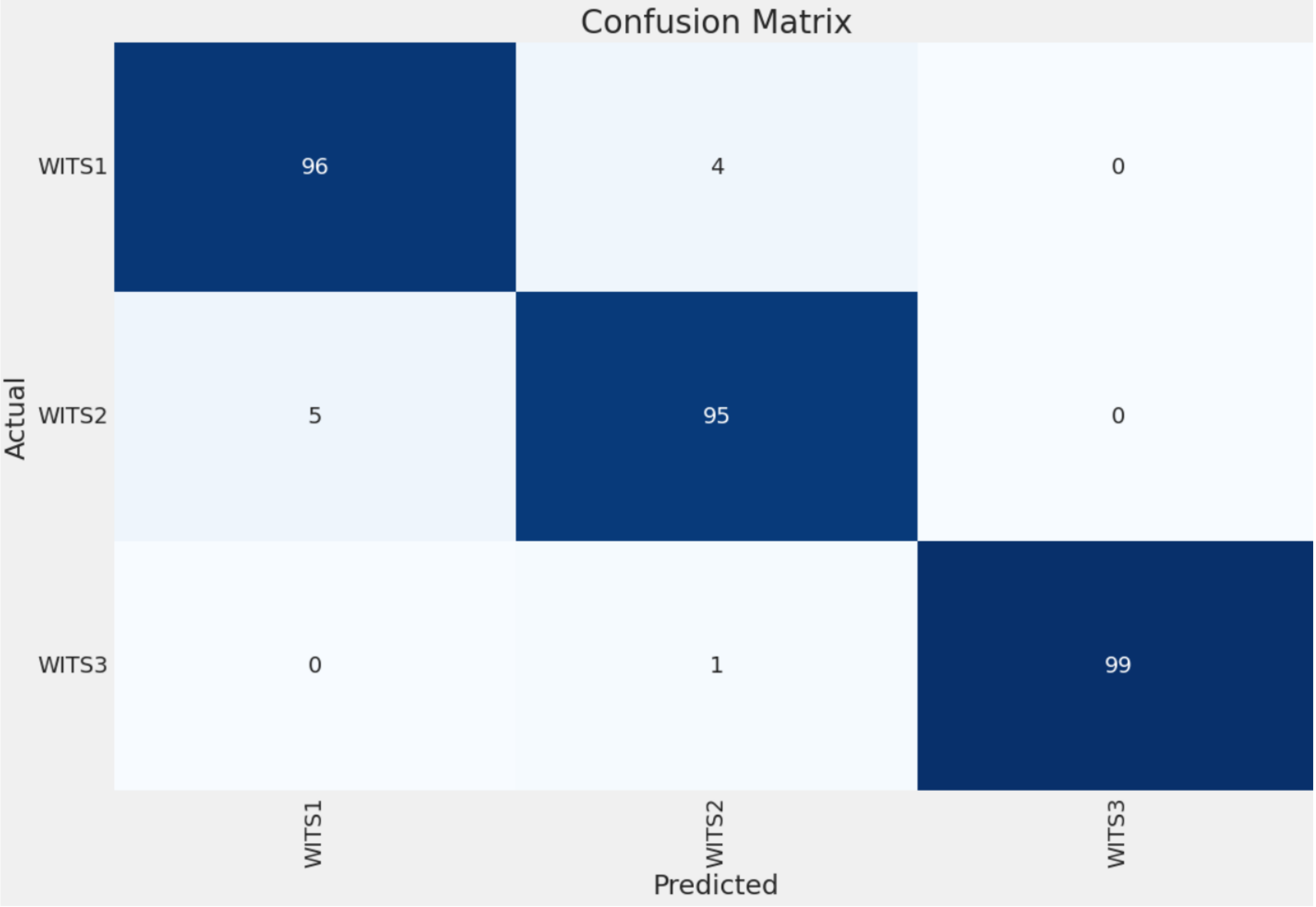

Table S42. Classification Report for WITS by INCEPTION V3

----

	precision	recall	f1-score	support
WITS1	0.9505	0.9600	0.9552	100
WITS2	0.9500	0.9500	0.9500	100
WITS3	1.0000	0.9900	0.9950	100

accuracy			0.9667	300
macro avg	0.9668	0.9667	0.9667	300
weighted avg	0.9668	0.9667	0.9667	300

Figure S77. Training and Validation Loss and Training and Accuracy Graphs for DENSENET 121

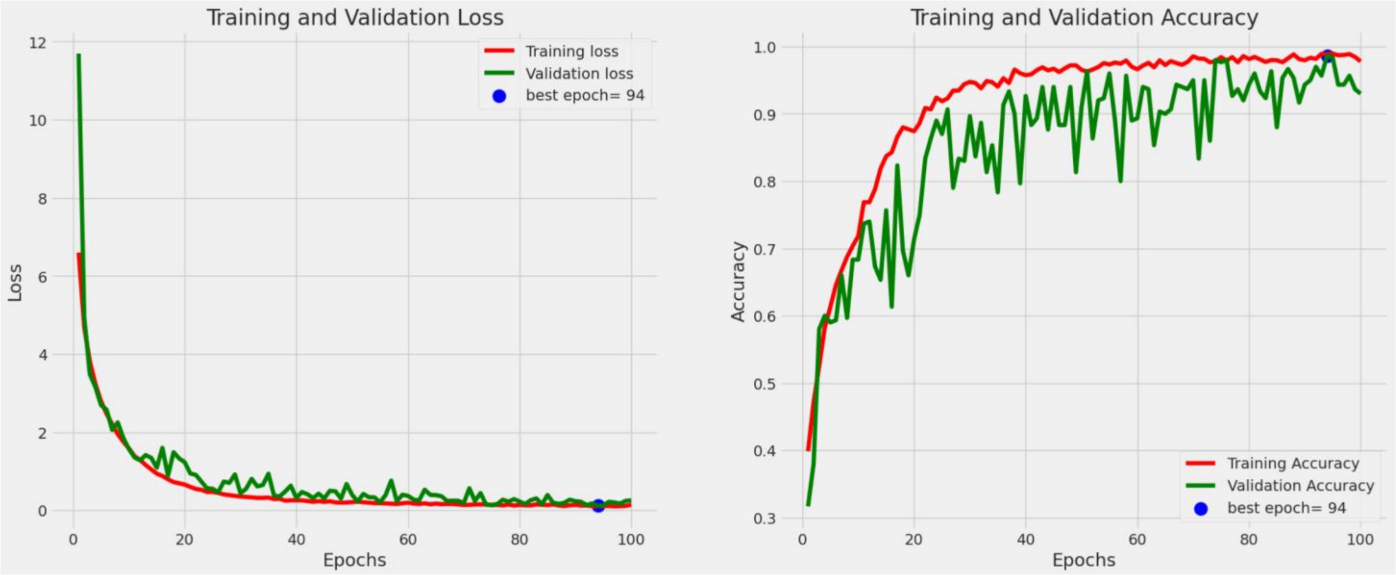

Figure S78. Confusion Matrix for Actual and Predicted WITS values by DENSENET 121

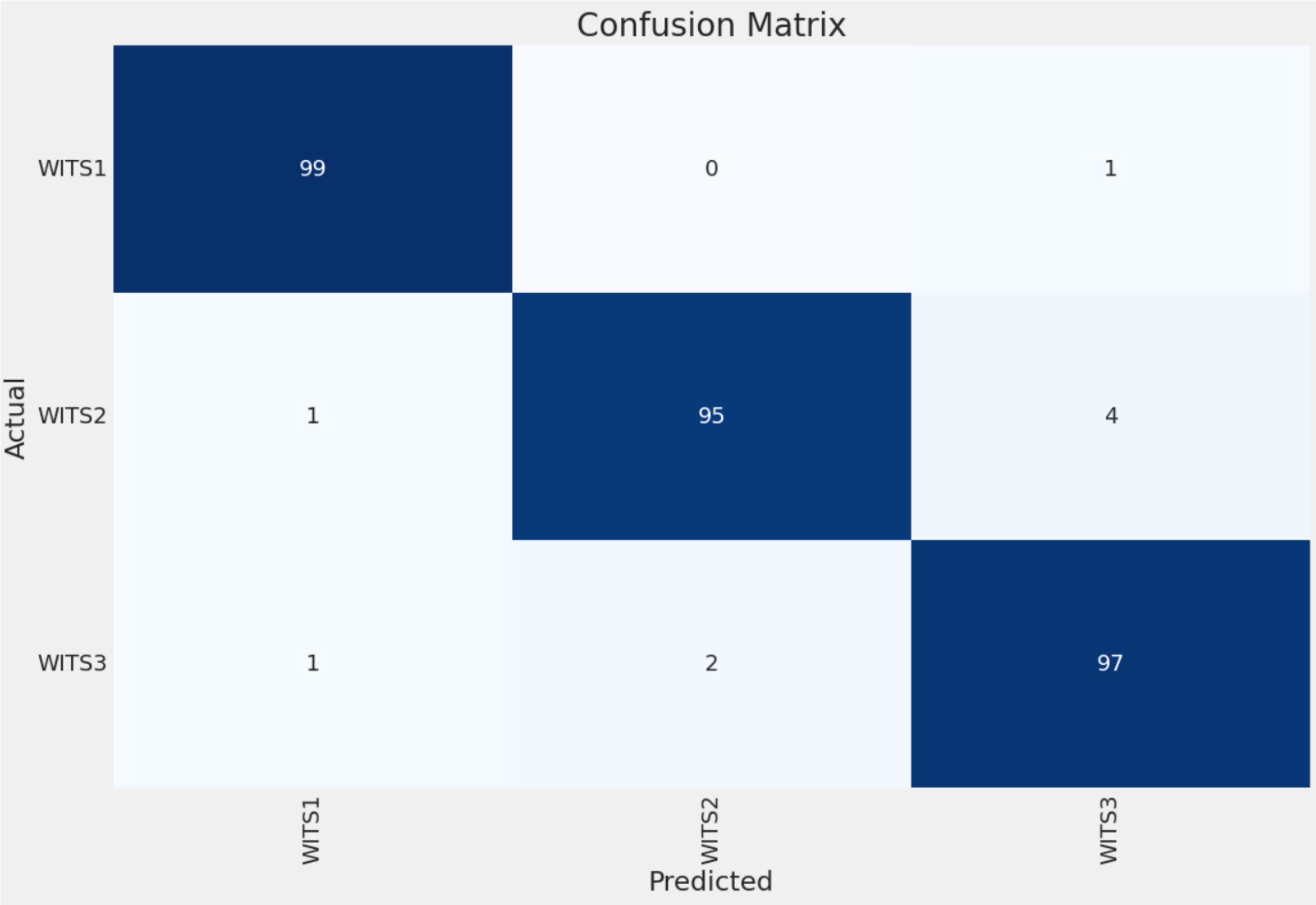

Table S43. Classification Report for WITS by DENSENET 121

	precision	recall	f1-score	support
WITS1	0.9802	0.9900	0.9851	100
WITS2	0.9794	0.9500	0.9645	100
WITS3	0.9510	0.9700	0.9604	100
accuracy			0.9700	300
macro avg	0.9702	0.9700	0.9700	300
weighted avg	0.9702	0.9700	0.9700	300

Figure S79. Training and Validation Loss and Training and Accuracy Graphs for DENSENET 169

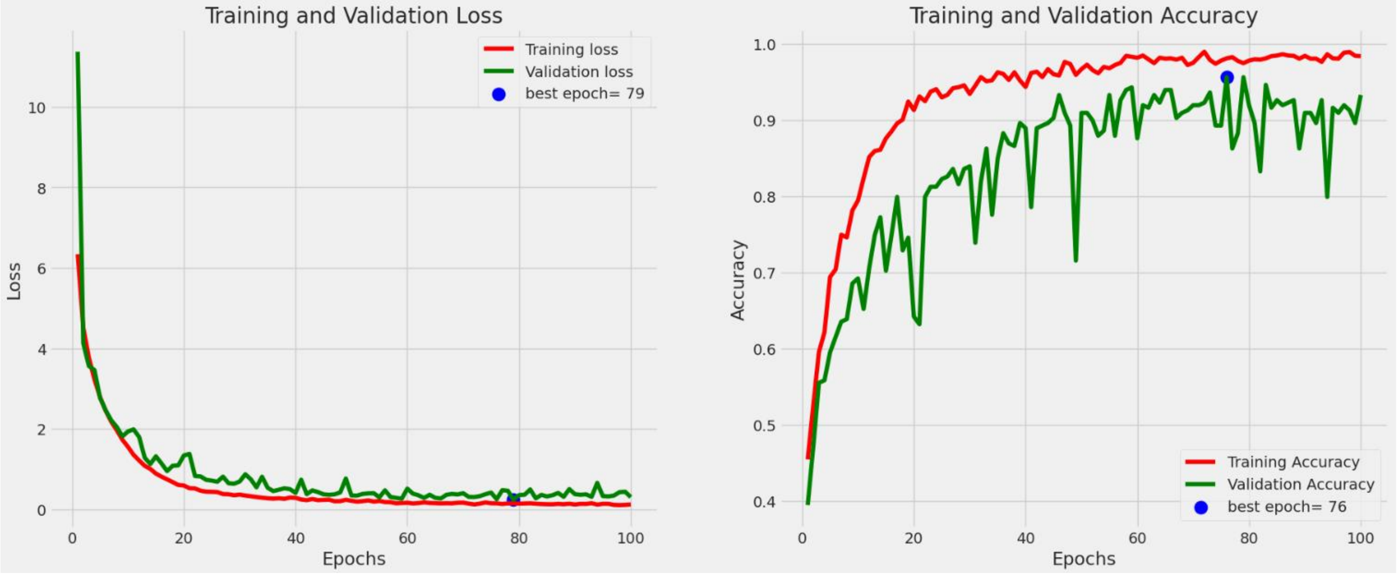

Figure S80. Confusion Matrix for Actual and Predicted WITS values by DENSENET 169

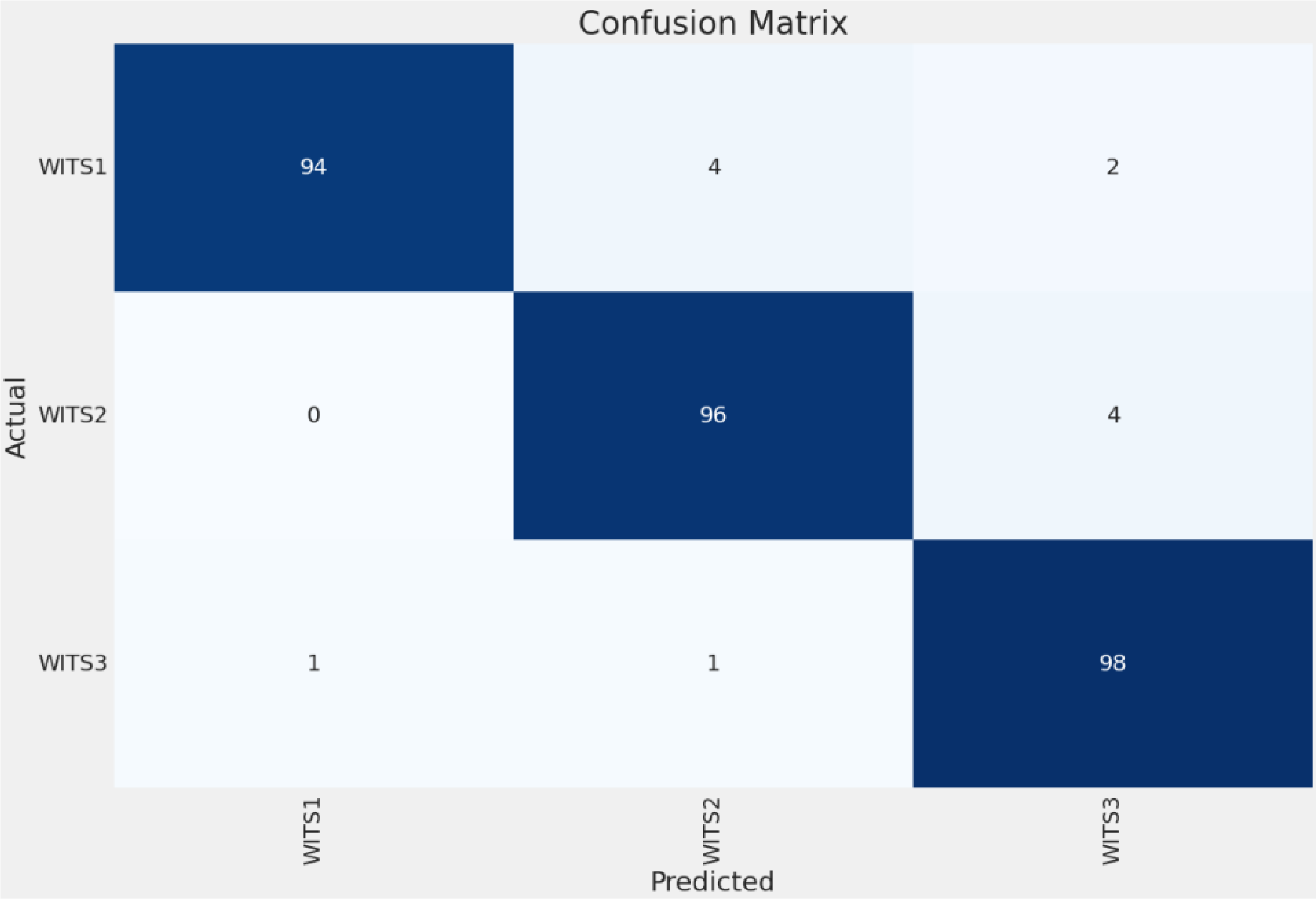

Table S44. Classification Report for WITS by DENSENET 169

----

	precision	recall	f1-score	support
WITS1	0.9895	0.9400	0.9641	100
WITS2	0.9505	0.9600	0.9552	100
WITS3	0.9423	0.9800	0.9608	100
accuracy			0.9600	300

macro avg	0.9608	0.9600	0.9600	300
weighted avg	0.9608	0.9600	0.9600	300

Figure S81. Training and Validation Loss and Training and Accuracy Graphs for DENSENET 201

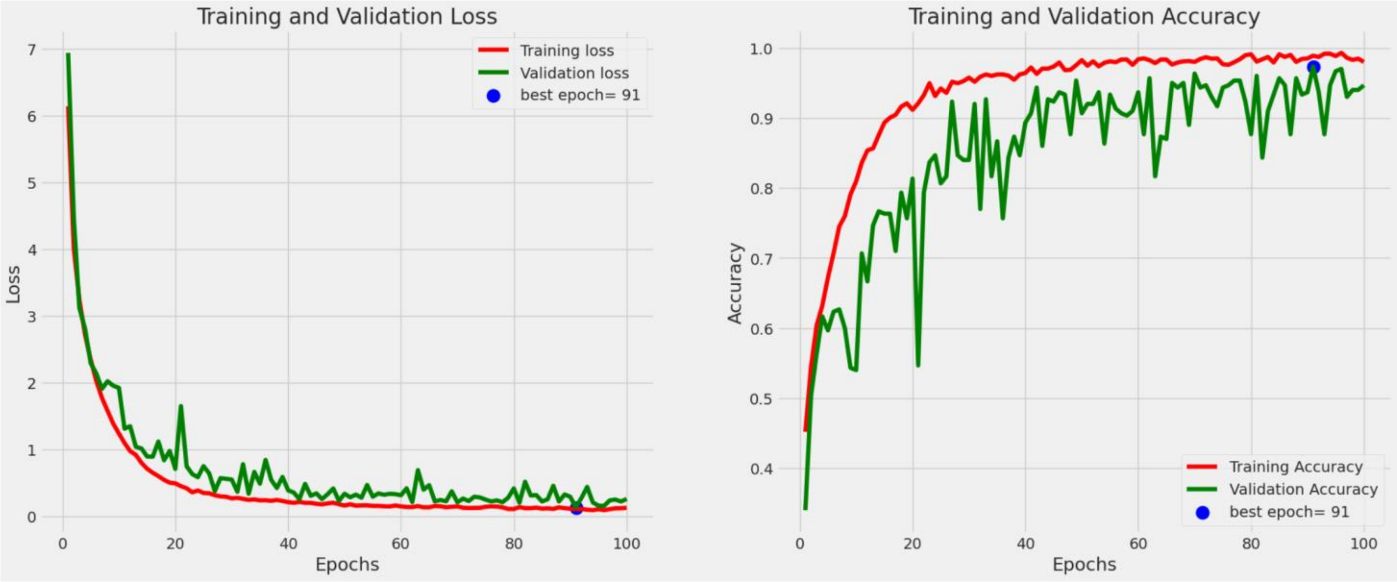

Figure S82. Confusion Matrix for Actual and Predicted WITS values by DENSENET 201

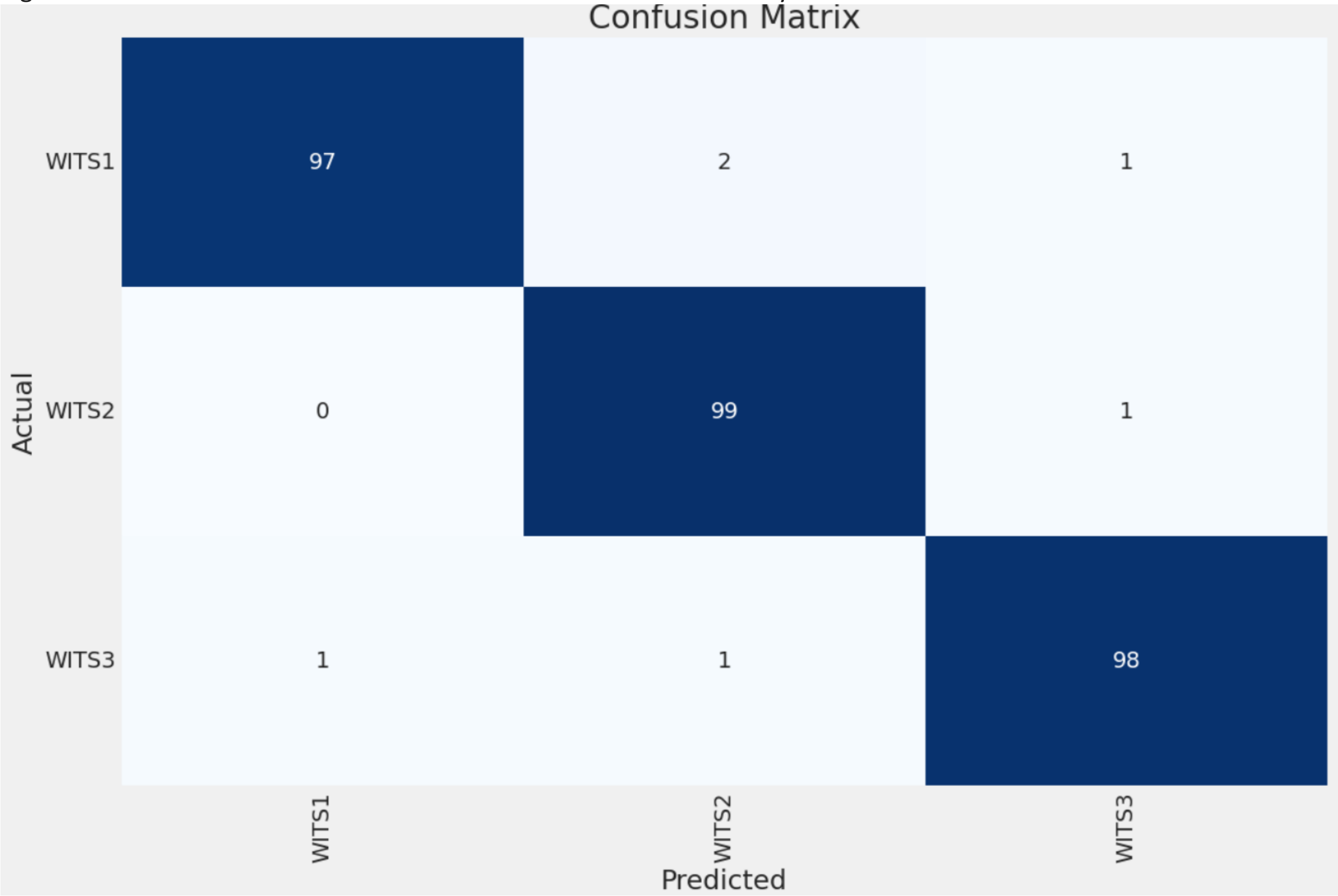

Table S45. Classification Report for WITS by DENSENET 201

	precision	recall	f1-score	support
WITS1	0.9898	0.9700	0.9798	100
WITS2	0.9706	0.9900	0.9802	100
WITS3	0.9800	0.9800	0.9800	100
accuracy			0.9800	300
macro avg	0.9801	0.9800	0.9800	300
weighted avg	0.9801	0.9800	0.9800	300

Figure S83. Training and Validation Loss and Training and Accuracy Graphs for EFFICIENTNET B0

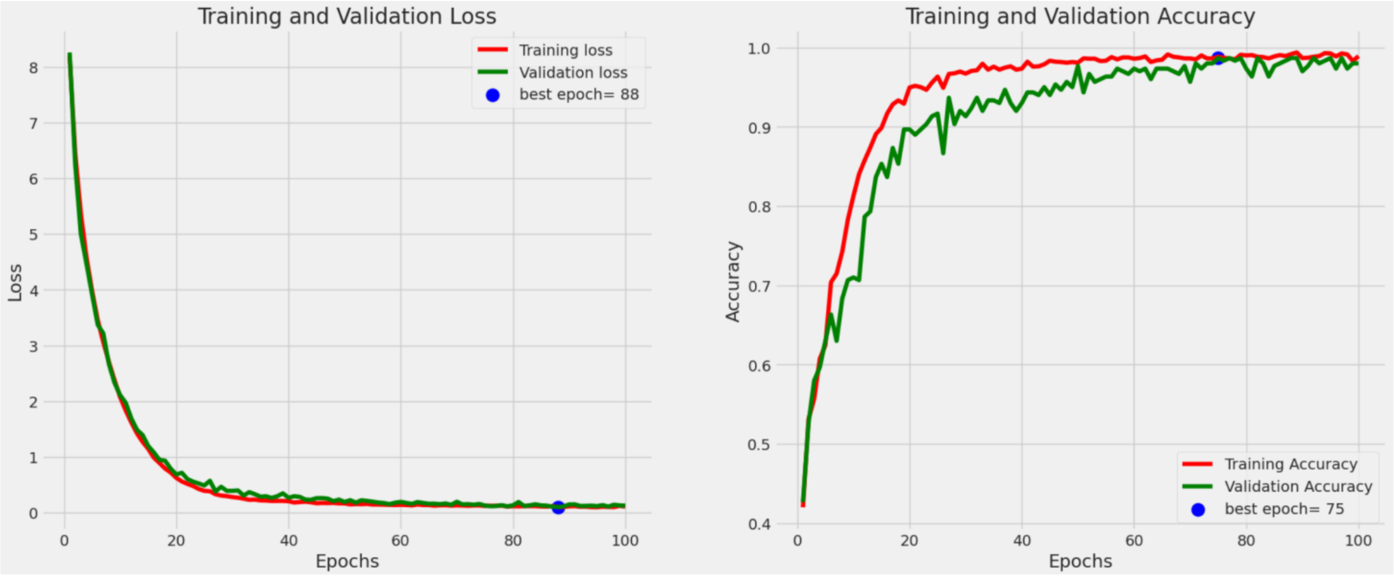

Figure S84. Confusion Matrix for Actual and Predicted WITS values by EFFICIENTNET B0

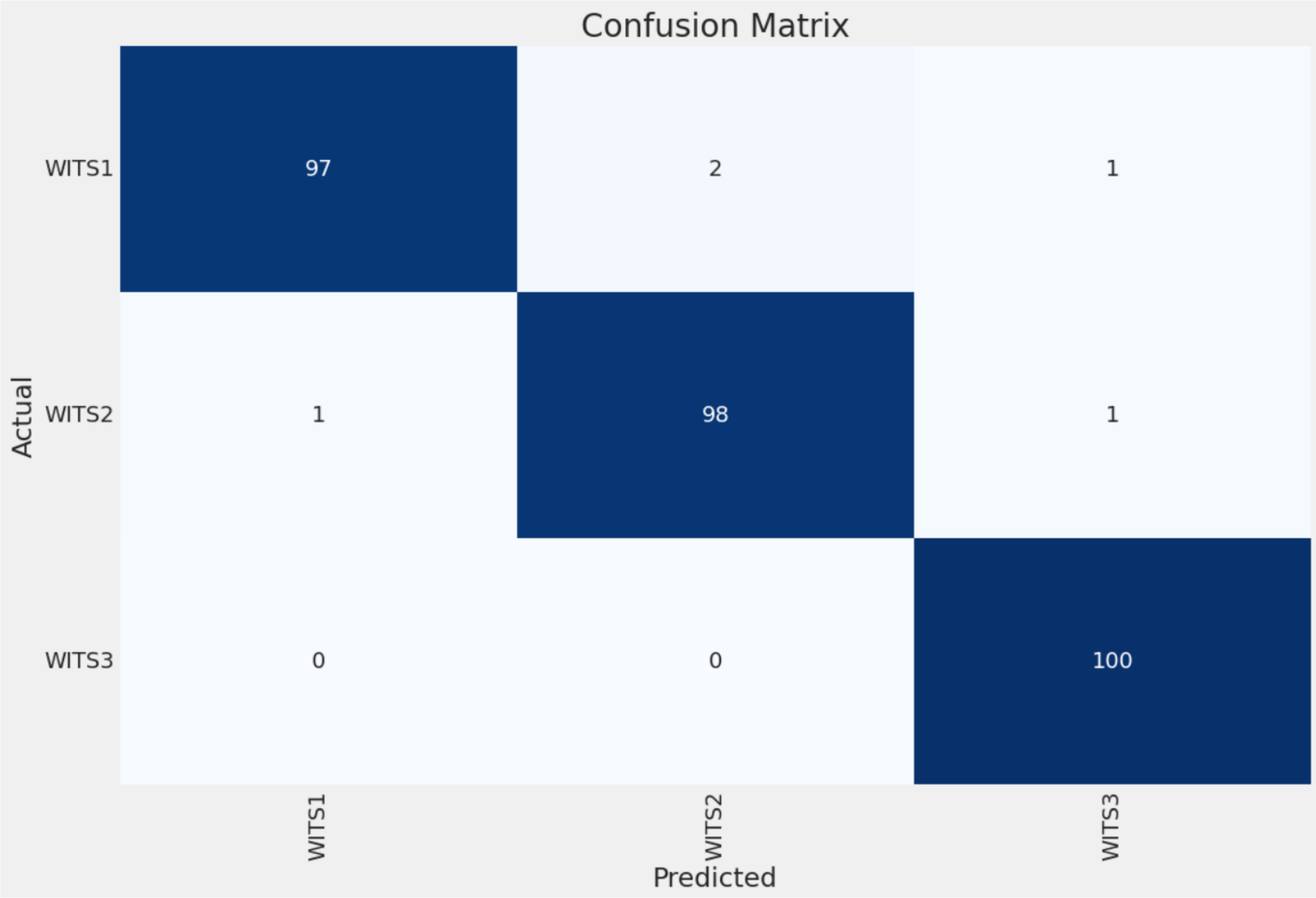

Table S46. Classification Report for WITS by EFFICIENTNET B0

----

	precision	recall	f1-score	support
WITS1	0.9898	0.9700	0.9798	100
WITS2	0.9800	0.9800	0.9800	100
WITS3	0.9804	1.0000	0.9901	100
accuracy			0.9833	300
macro avg	0.9834	0.9833	0.9833	300
weighted avg	0.9834	0.9833	0.9833	300

Figure S85. Training and Validation Loss and Training and Accuracy Graphs for XCEPTION

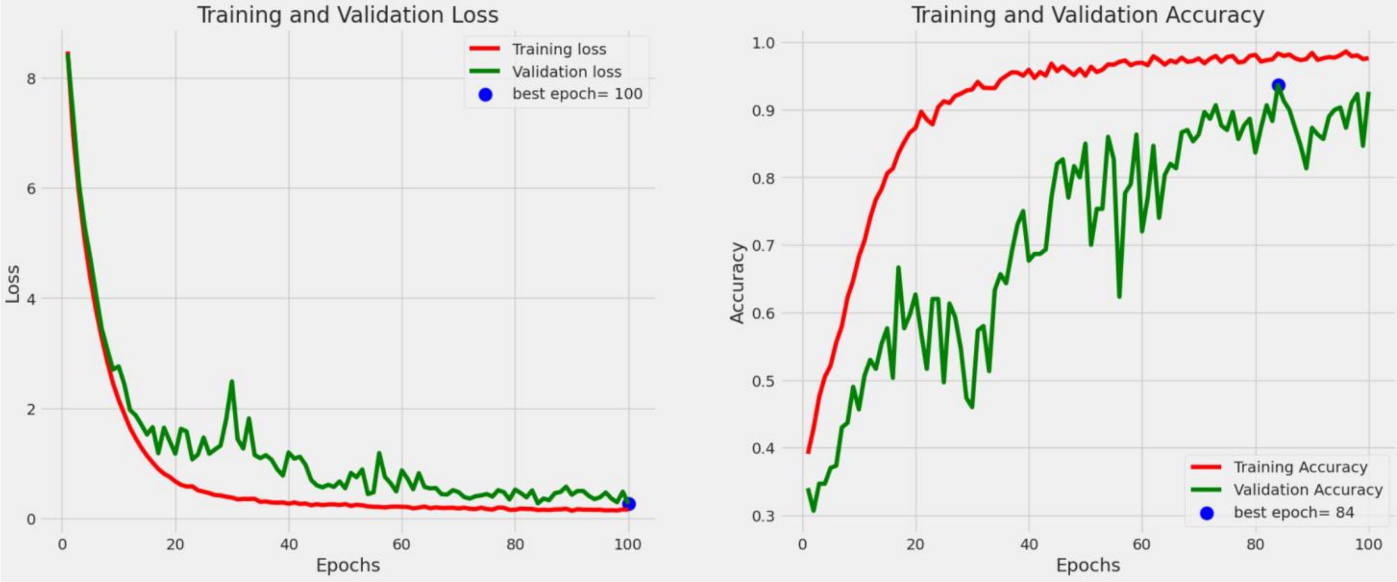

Figure S86. Confusion Matrix for Actual and Predicted WITS values by XCEPTION

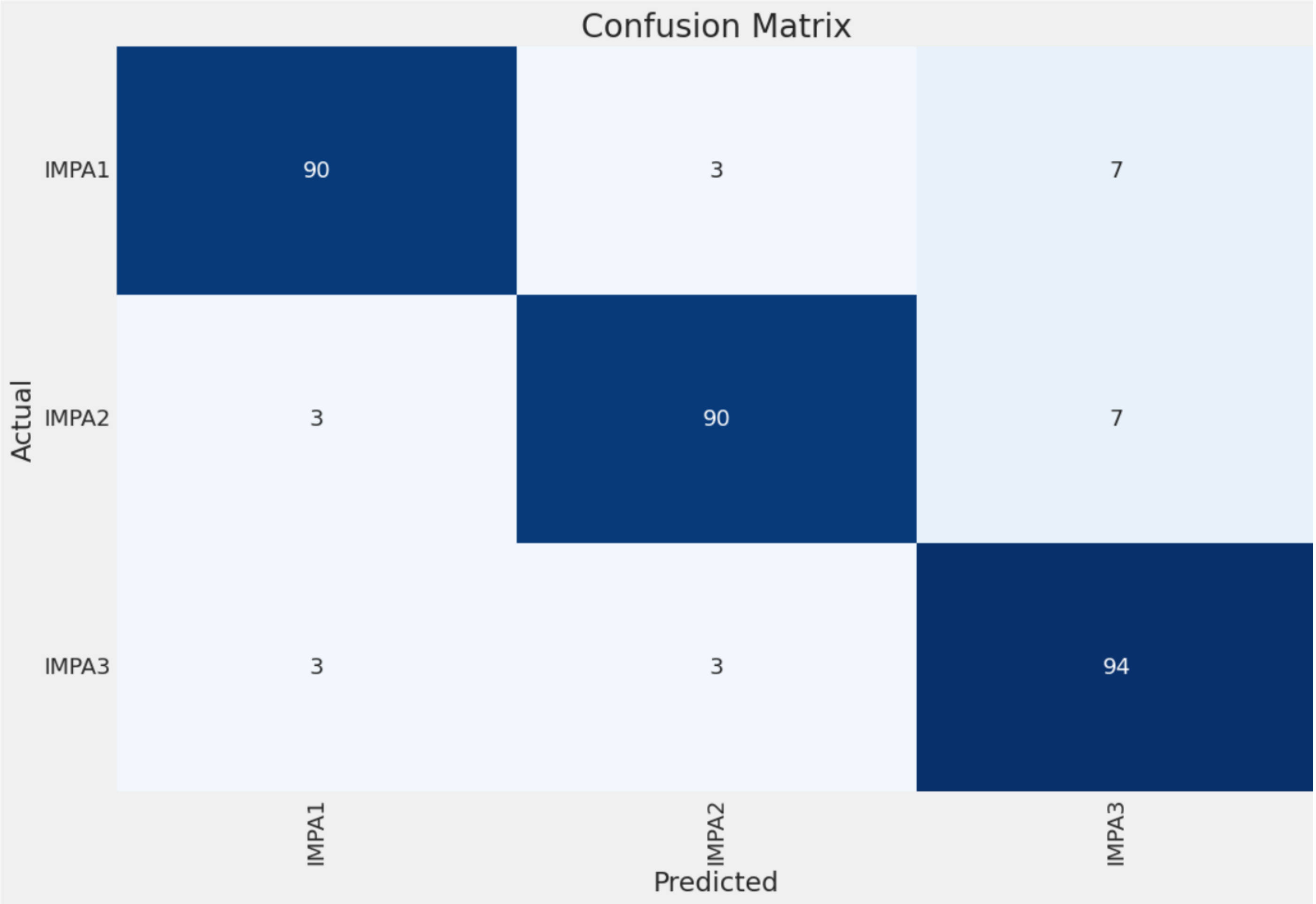

Table S47. Classification Report for WITS by XCEPTION

	precision	recall	f1-score	support
IMPA1	0.9375	0.9000	0.9184	100
IMPA2	0.9375	0.9000	0.9184	100
IMPA3	0.8704	0.9400	0.9038	100
accuracy			0.9133	300
macro avg	0.9151	0.9133	0.9135	300

```
weighted avg      0.9151     0.9133     0.9135       300
```

Figure S87. Training and Validation Loss and Training and Accuracy Graphs for VGG16

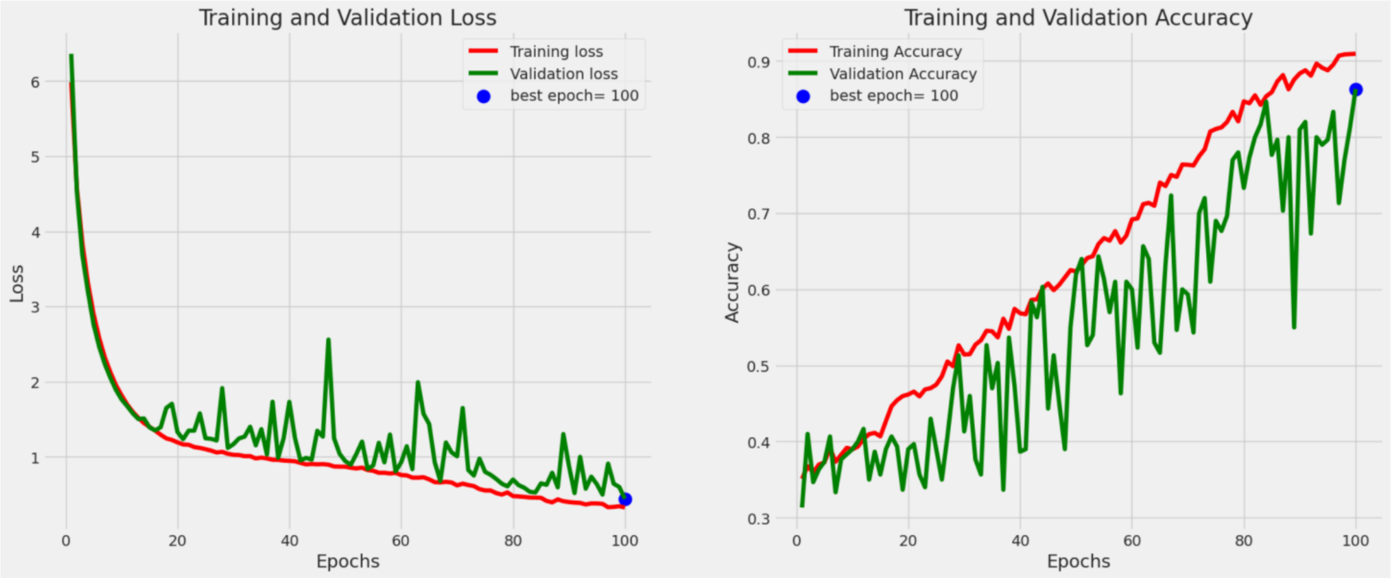

Figure S88. Confusion Matrix for Actual and Predicted WITS values by VGG16

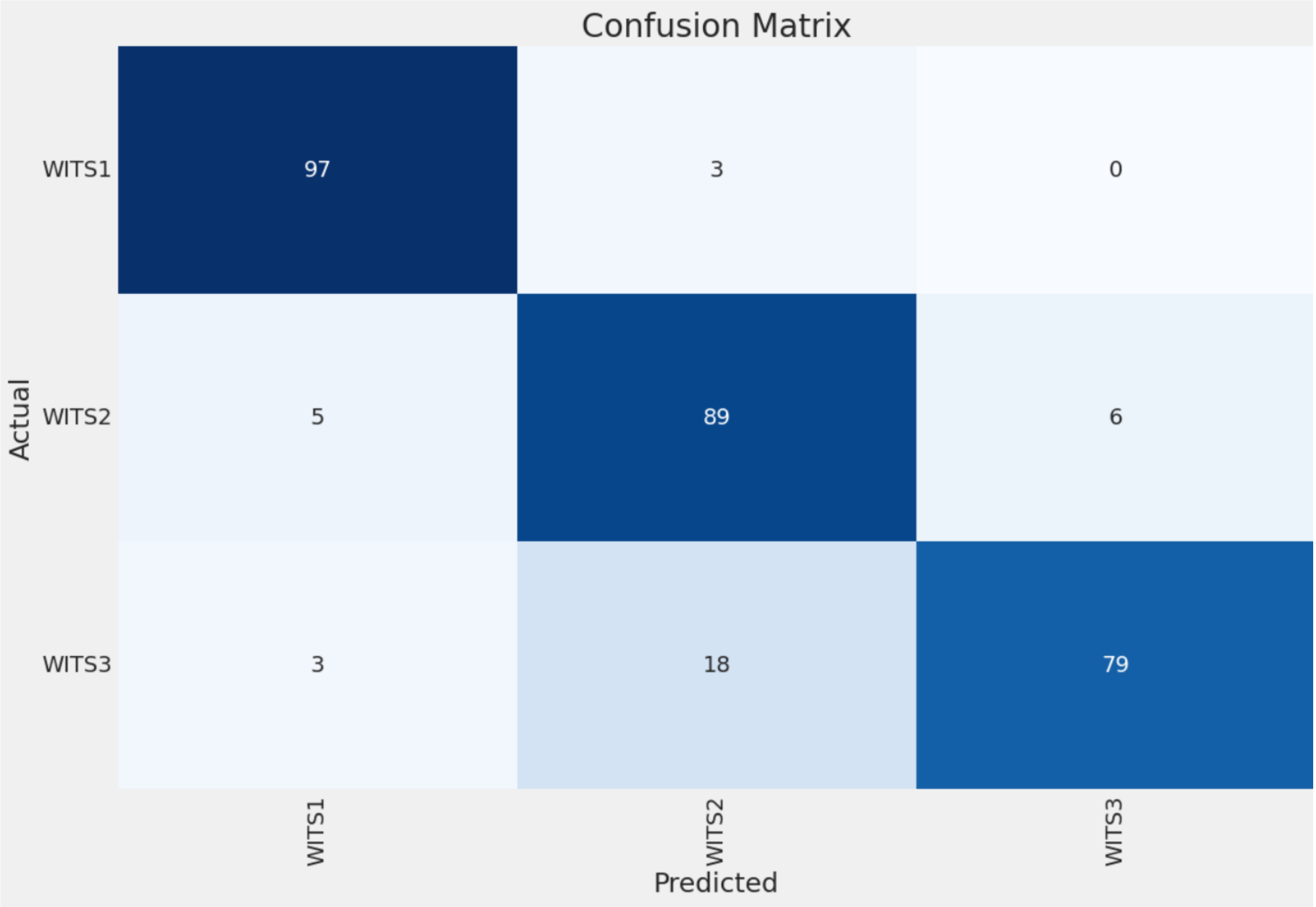

Table S48. Classification Report for WITS by VGG16

	precision	recall	f1-score	support
WITS1	0.9238	0.9700	0.9463	100
WITS2	0.8091	0.8900	0.8476	100
WITS3	0.9294	0.7900	0.8541	100
accuracy			0.8833	300
macro avg	0.8874	0.8833	0.8827	300
weighted avg	0.8874	0.8833	0.8827	300

Figure S89. Training and Validation Loss and Training and Accuracy Graphs for VGG19

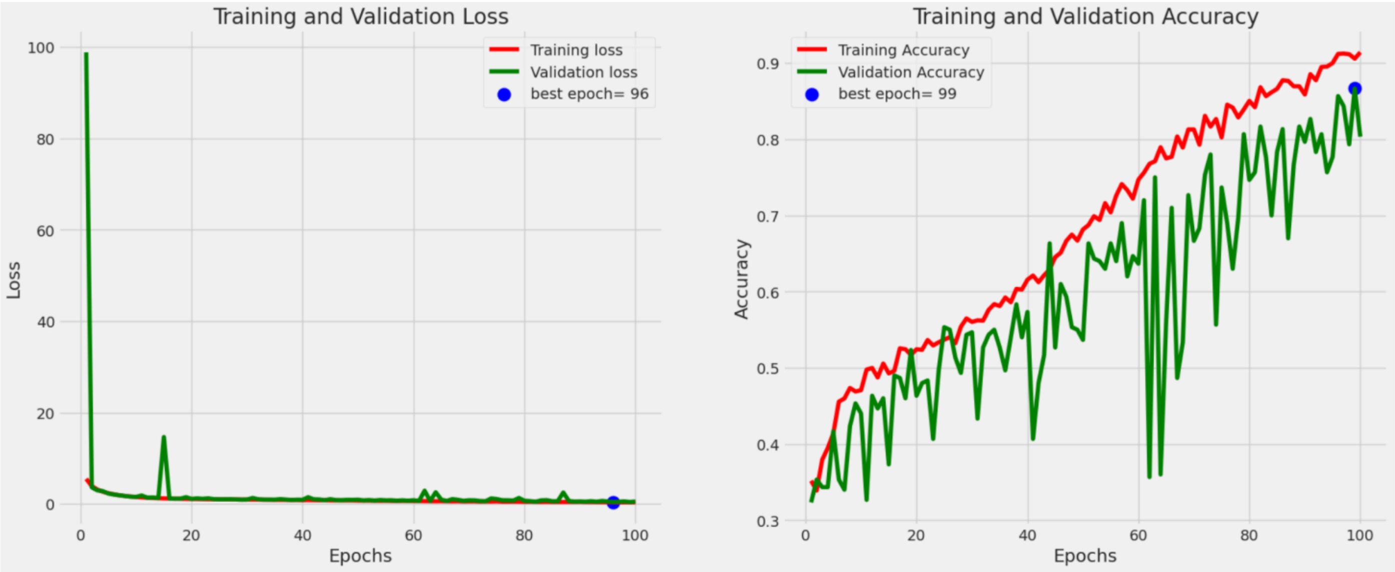

Figure S90. Confusion Matrix for Actual and Predicted WITS values by VGG19

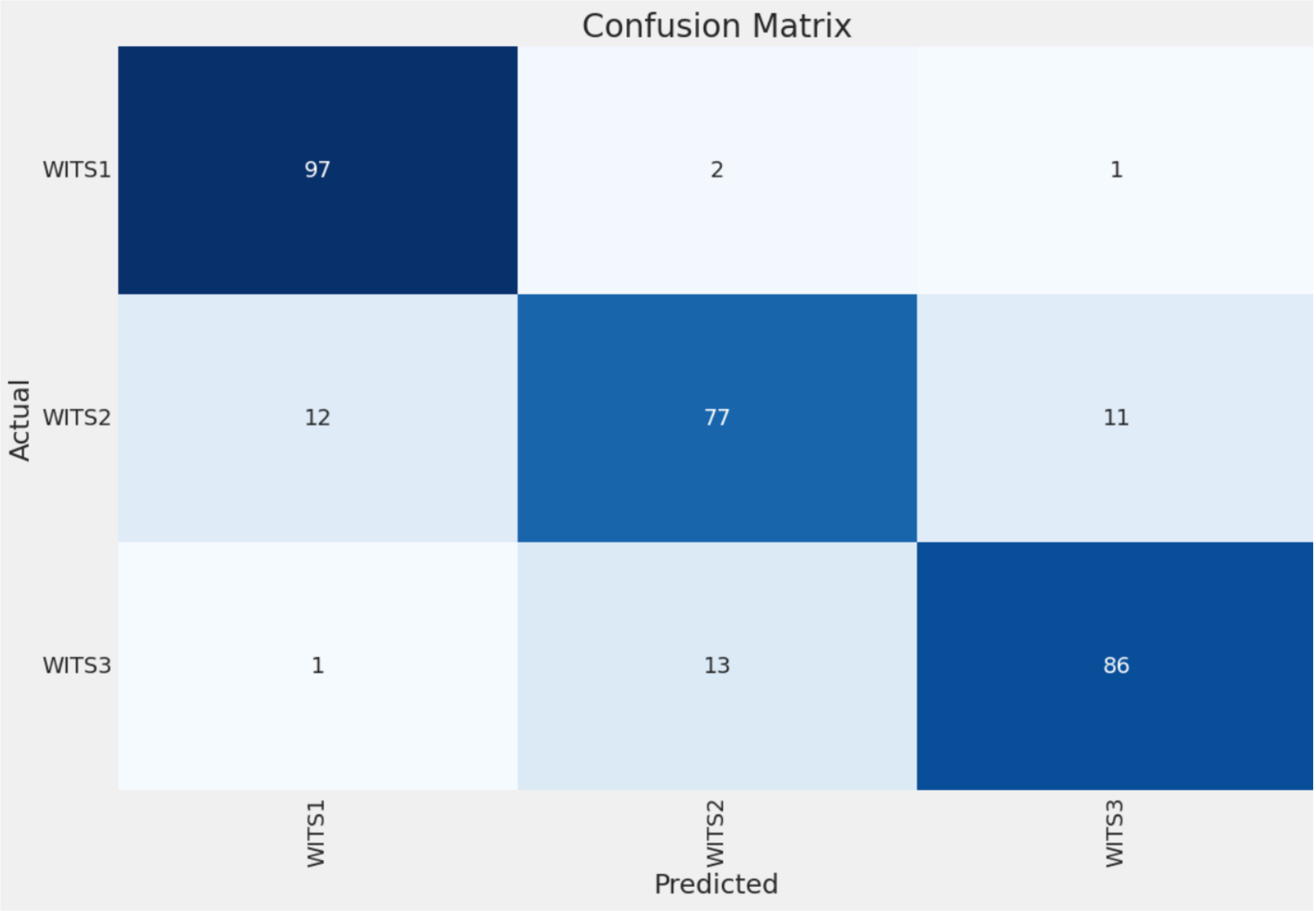

Table S49. Classification Report for WITS by VGG19

	precision	recall	f1-score	support
WITS1	0.8818	0.9700	0.9238	100
WITS2	0.8370	0.7700	0.8021	100
WITS3	0.8776	0.8600	0.8687	100

accuracy			0.8667	300
macro avg	0.8654	0.8667	0.8649	300
weighted avg	0.8654	0.8667	0.8649	300

Figure S91. Training and Validation Loss and Training and Accuracy Graphs for NASNETMOBILE

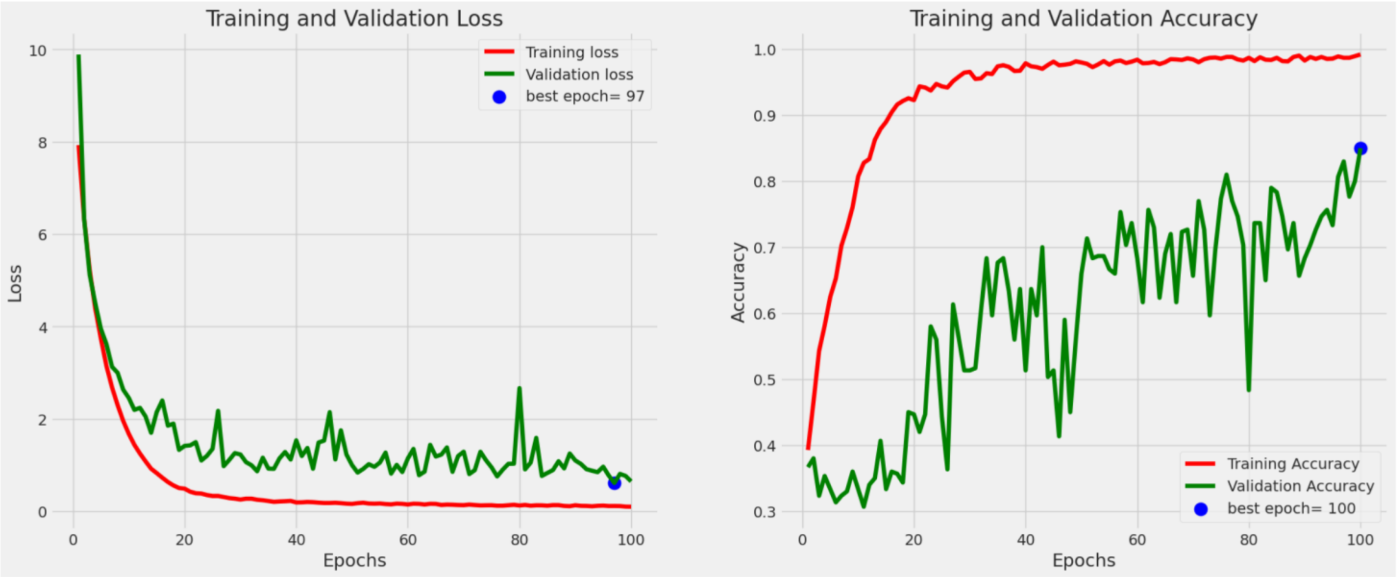

Figure S92. Confusion Matrix for Actual and Predicted WITS values by NASNETMOBILE

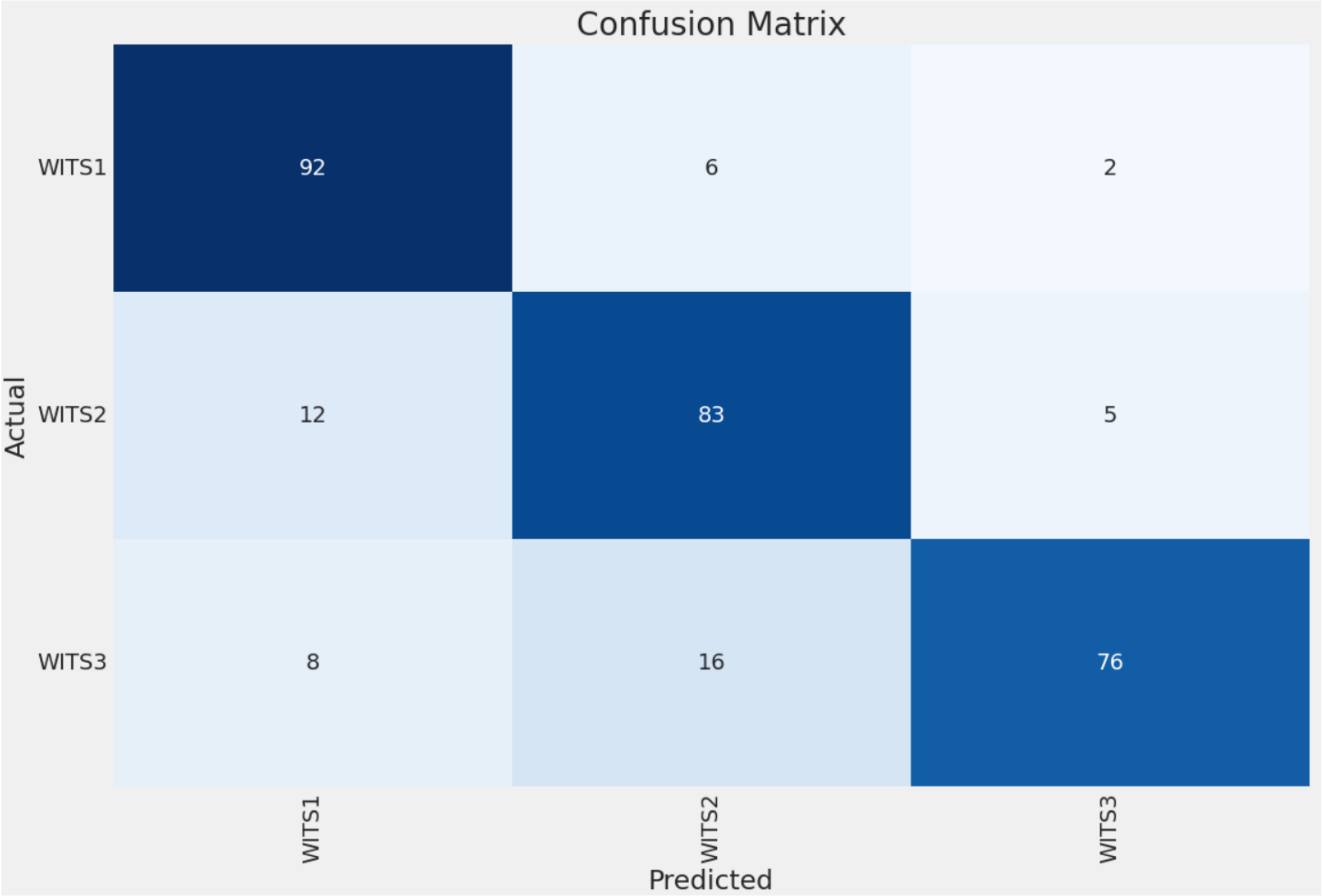

Table S50. Classification Report for WITS by NASNETMOBILE

----

	precision	recall	f1-score	support
WITS1	0.8214	0.9200	0.8679	100
WITS2	0.7905	0.8300	0.8098	100
WITS3	0.9157	0.7600	0.8306	100
accuracy			0.8367	300
macro avg	0.8425	0.8367	0.8361	300
weighted avg	0.8425	0.8367	0.8361	300

Figure S93. Training and Validation Loss and Training and Accuracy Graphs for RESNET101

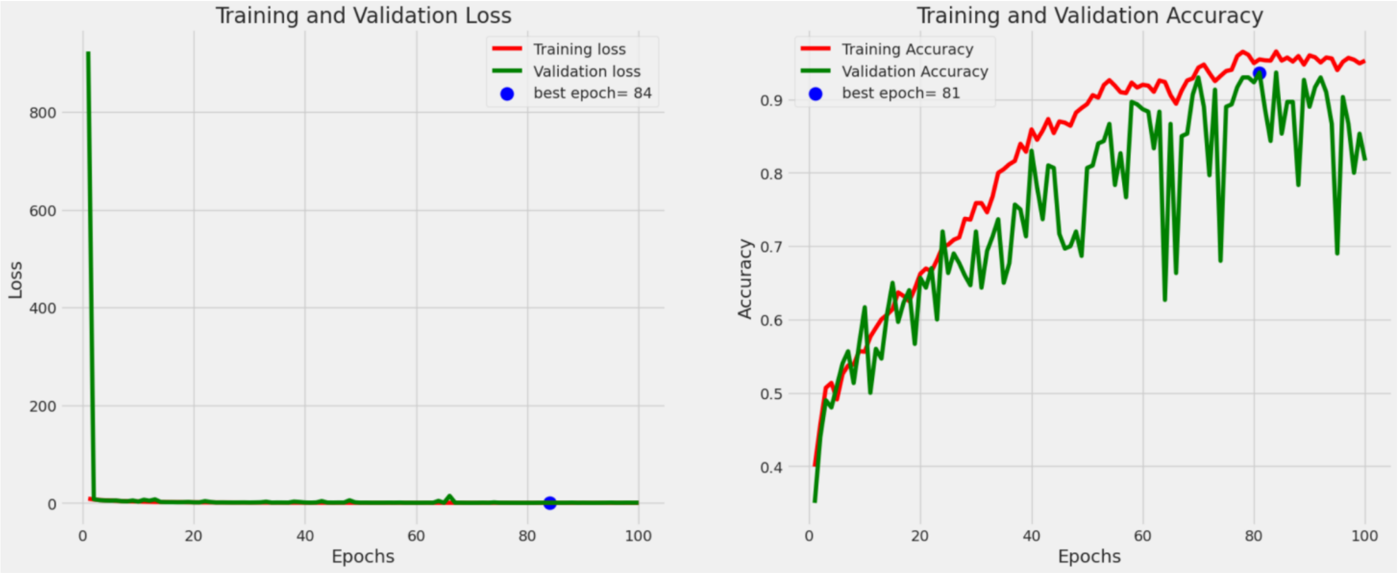

Figure S94. Confusion Matrix for Actual and Predicted WITS values by RESNET101

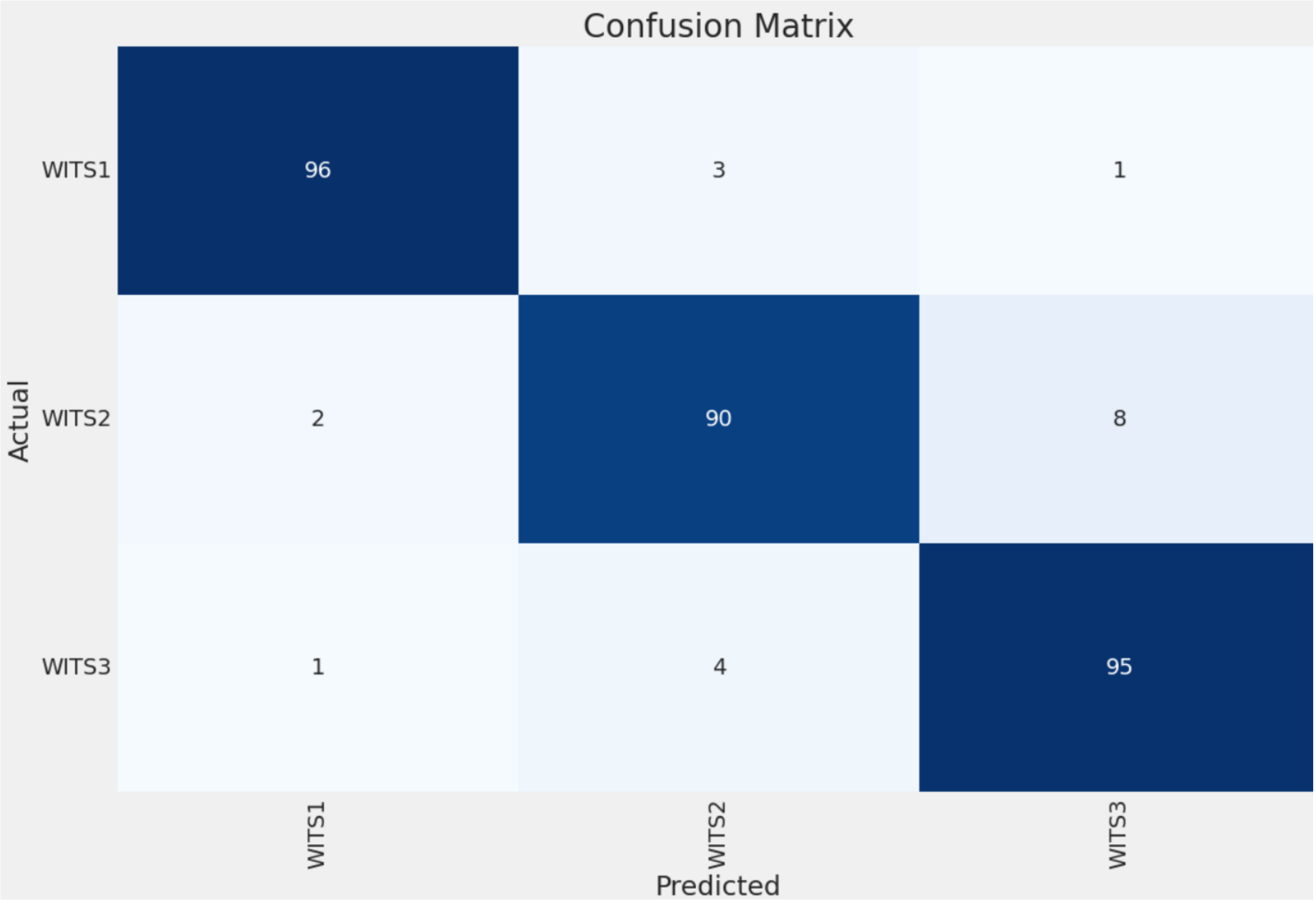

Table S51. Classification Report for WITS by RESNET101

	precision	recall	f1-score	support
WITS1	0.9697	0.9600	0.9648	100
WITS2	0.9278	0.9000	0.9137	100
WITS3	0.9135	0.9500	0.9314	100
accuracy			0.9367	300
macro avg	0.9370	0.9367	0.9366	300
weighted avg	0.9370	0.9367	0.9366	300

Figure S95. Training and Validation Loss and Training and Accuracy Graphs for RESNET152

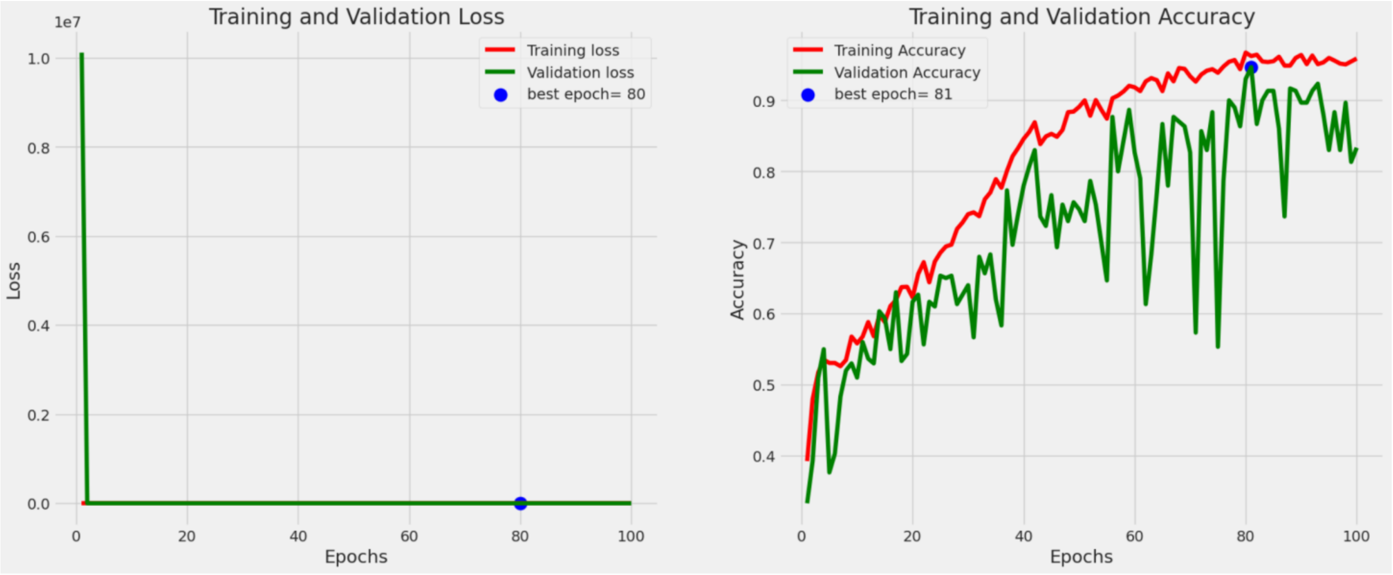

Figure S96. Confusion Matrix for Actual and Predicted WITS values by RESNET152

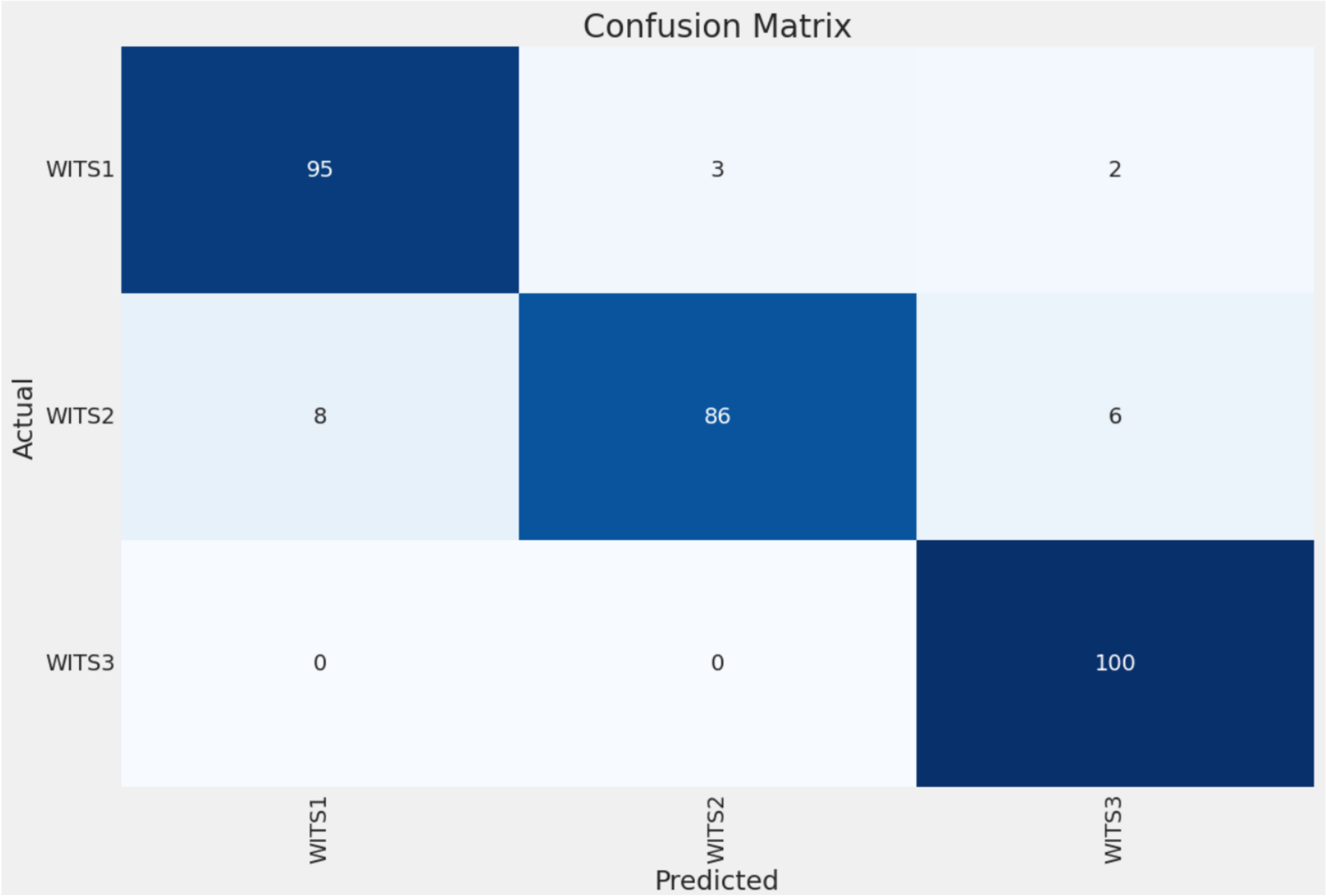

Table S52. Classification Report for WITS by RESNET152

	precision	recall	f1-score	support
WITS1	0.9223	0.9500	0.9360	100
WITS2	0.9663	0.8600	0.9101	100
WITS3	0.9259	1.0000	0.9615	100

accuracy			0.9367	300
macro avg	0.9382	0.9367	0.9359	300
weighted avg	0.9382	0.9367	0.9359	300

Figure S97. Training and Validation Loss and Training and Accuracy Graphs for RESNET50

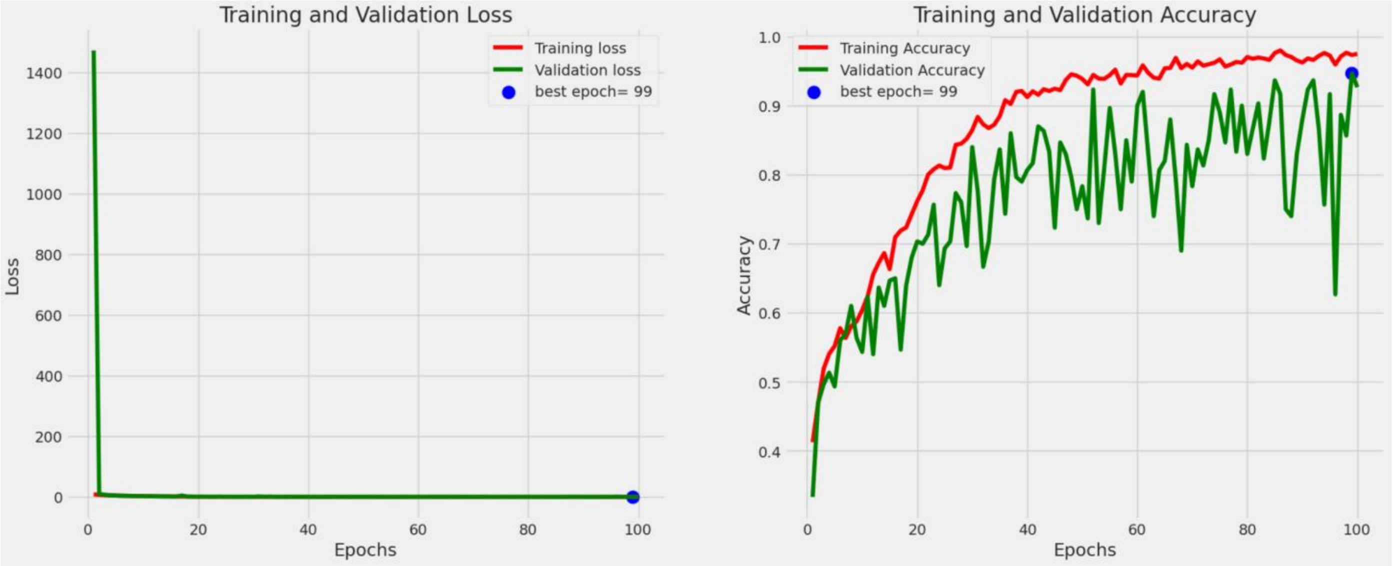

Figure S98. Confusion Matrix for Actual and Predicted WITS values by RESNET50

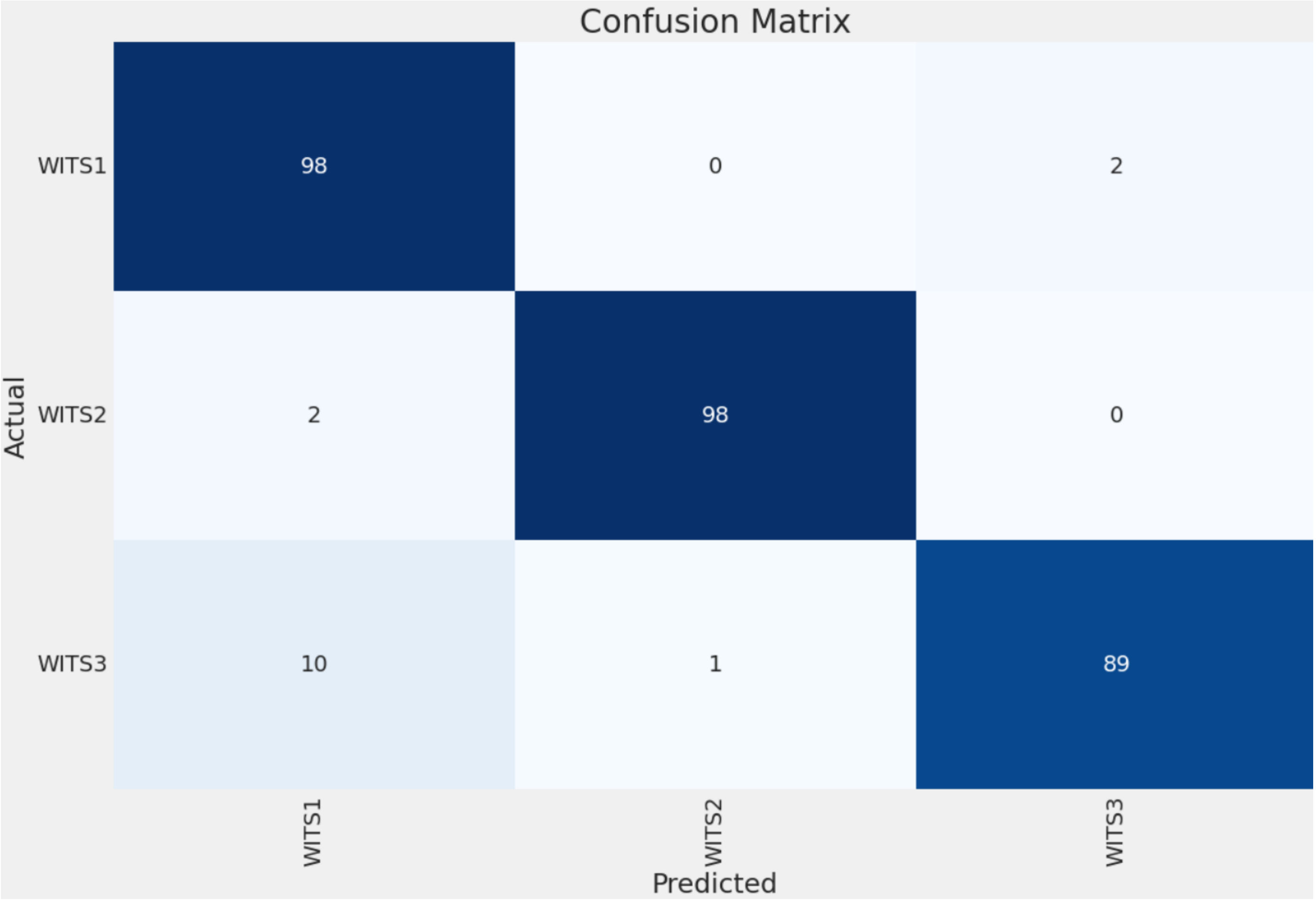

Table S53. Classification Report for WITS by RESNET50

----

	precision	recall	f1-score	support
WITS1	0.8909	0.9800	0.9333	100
WITS2	0.9899	0.9800	0.9849	100
WITS3	0.9780	0.8900	0.9319	100
accuracy			0.9500	300
macro avg	0.9529	0.9500	0.9501	300
weighted avg	0.9529	0.9500	0.9501	300

Figure S99. Training and Validation Loss and Training and Accuracy Graphs for EFFICIENTNET V2

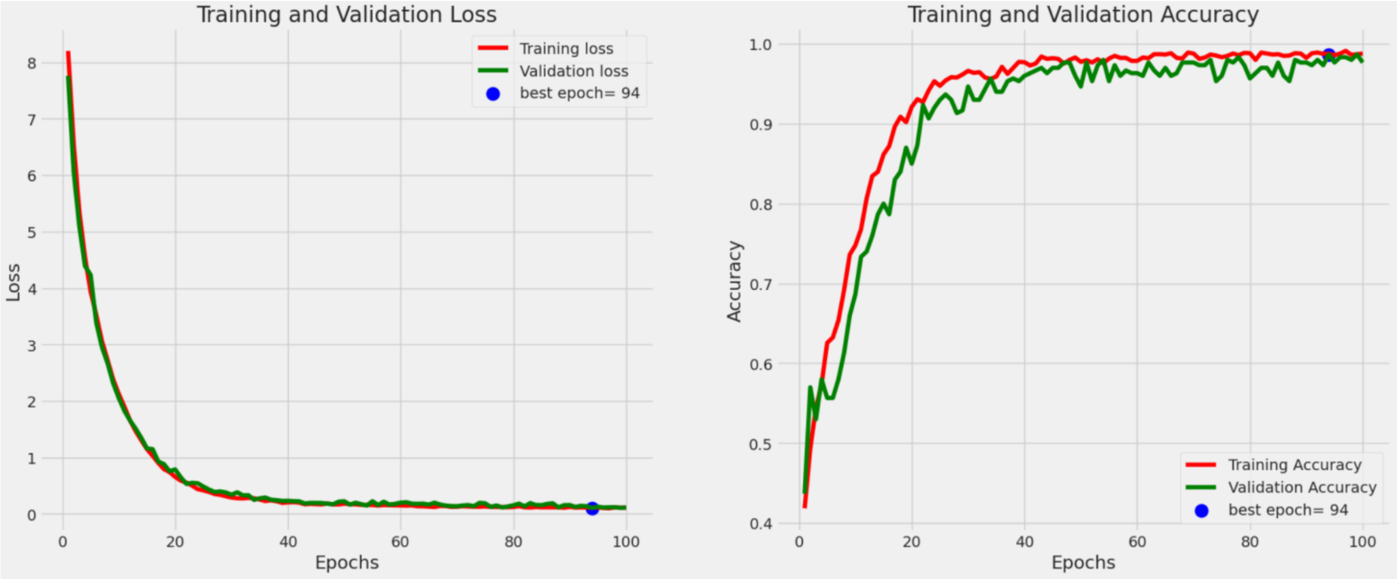

Figure S100. Confusion Matrix for Actual and Predicted WITS values by EFFICIENTNET V2

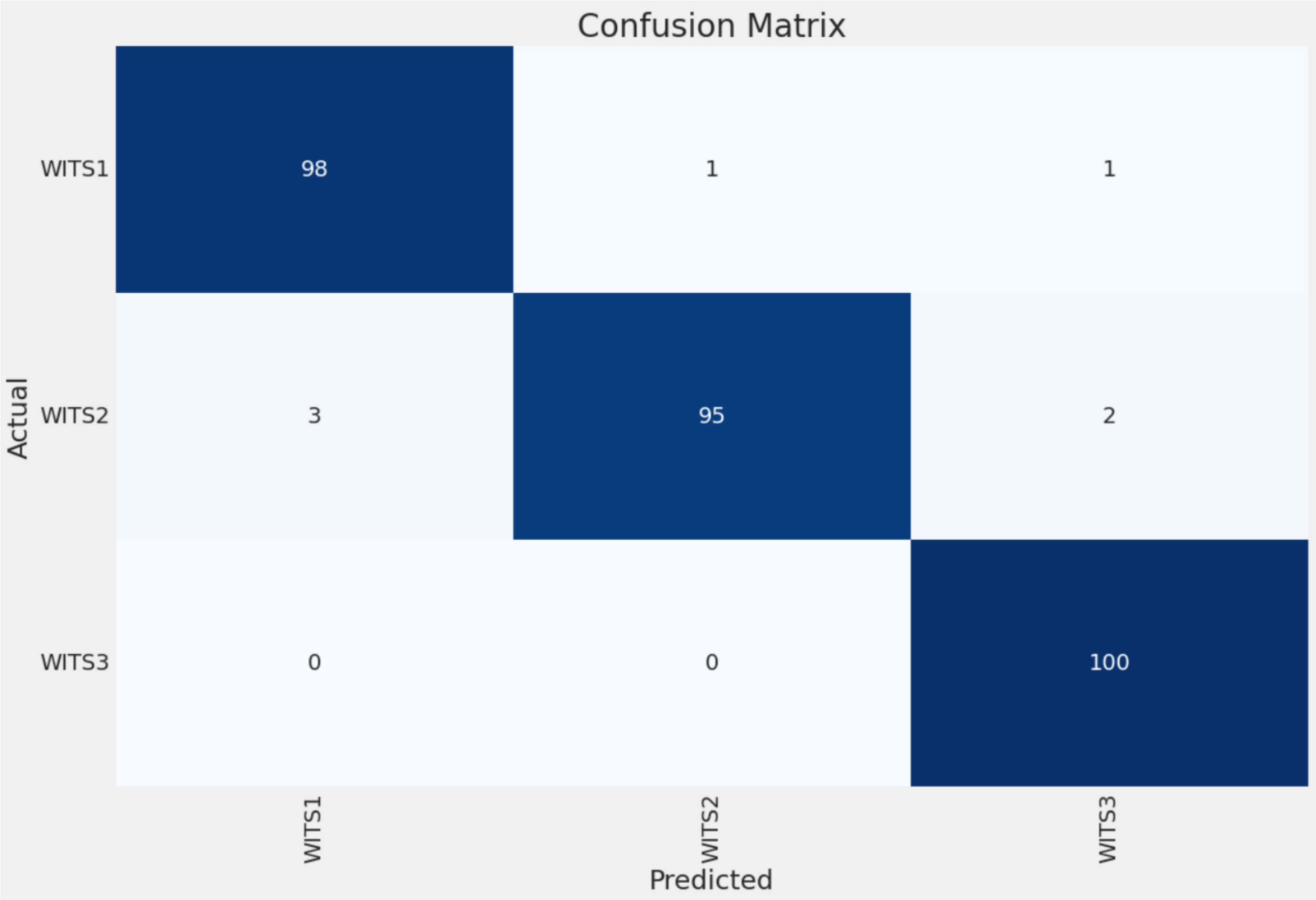

Table S54. Classification Report for WITS by EFFICIENTNET V2

----

	precision	recall	f1-score	support
WITS1	0.9703	0.9800	0.9751	100
WITS2	0.9896	0.9500	0.9694	100
WITS3	0.9709	1.0000	0.9852	100

accuracy			0.9767	300
macro avg	0.9769	0.9767	0.9766	300
weighted avg	0.9769	0.9767	0.9766	300
